# A flexible and scalable synthesis of 4′-thionucleosides†

**DOI:** 10.1039/d4sc05679e

**Published:** 2024-11-27

**Authors:** Callum Lucas, Ethan Fung, Matthew Nodwell, Steven Silverman, Bara Singh, Louis-Charles Campeau, Robert Britton

**Affiliations:** a Department of Chemistry, Simon Fraser University Burnaby British Columbia V5A 1S6 Canada rbritton@sfu.ca; b Department of Process Research & Development, Merck & Co., Inc. Rahway NJ 07065 USA

## Abstract

4′-Thionucleosides (thNAs) are synthetic nucleoside analogues that have attracted attention as leads for drug discovery in oncology and virology. Here we report a *de novo* thNA synthesis that relies on a scalable α-fluorination and aldol reaction of α-heteroaryl acetaldehydes followed by a streamlined process involving carbonyl reduction, mesylate formation and a double displacement reaction using NaSH. We demonstrate the multigram preparation of 4′-thio-5-methyluridine and highlight the production of purine and pyrimidine thNAs as well as C2′-modified thNAs.

## Abstract 2

A short, *de novo* synthesis of purine- and pyrimidine-substituted 4′-thionucleosides is reported. The scalability of this process as well as its utility for accessing C2′-modified thionucleosides is demonstrated.

## Graphical abstract

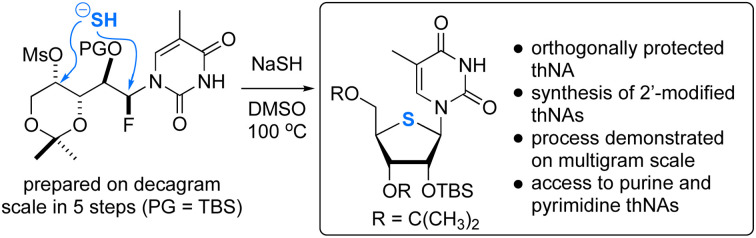

## Introduction

**Fig. 1:**
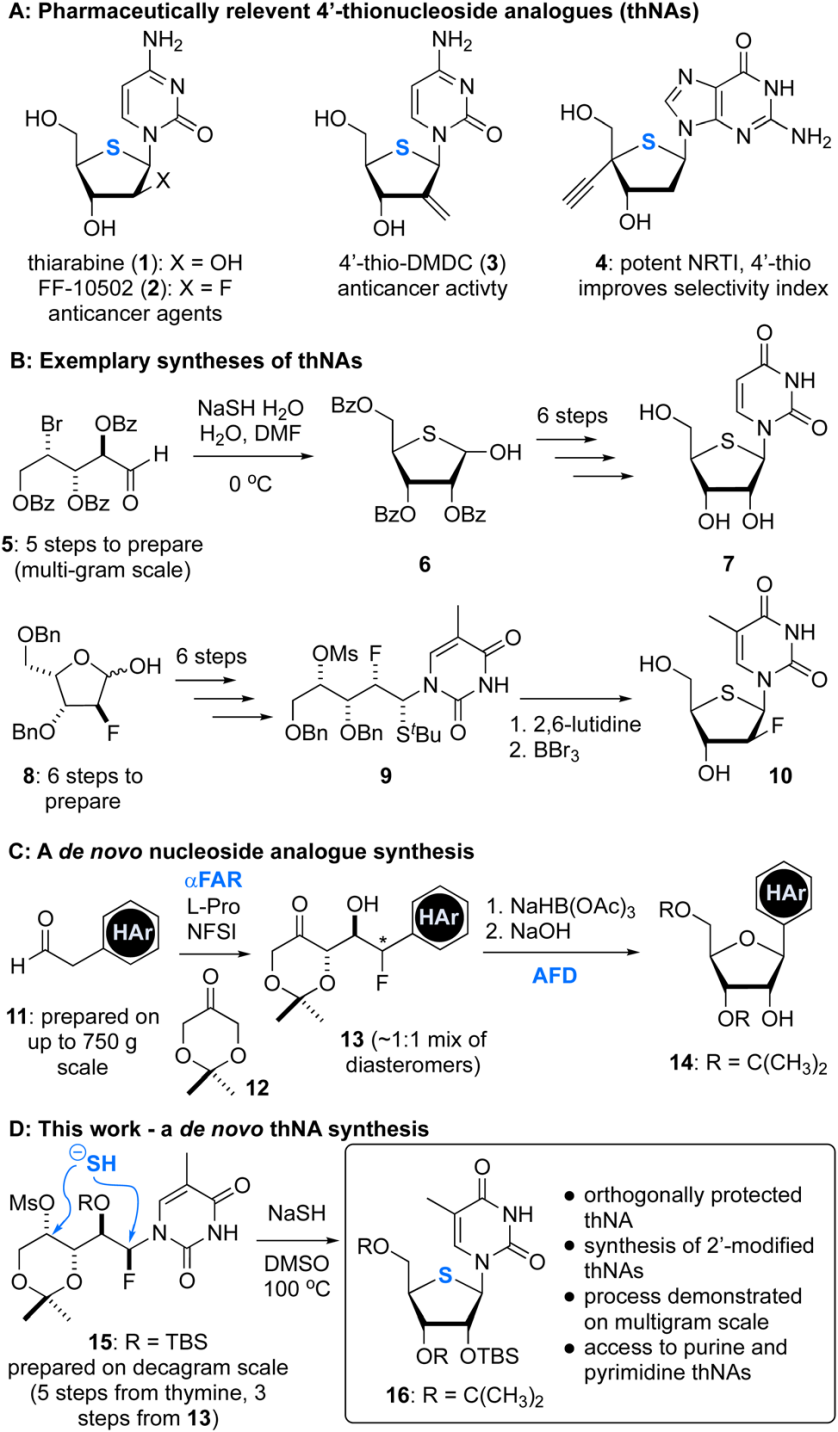
4′-Thionucleoside analogues (thNAs) and strategies used to prepare these compounds. (A) Examples of pharmaceutically relevnt 4′-thionucleosides. (B) Syntheses of 4′-thionucleosides. (C) A one-pot organocatalytic α-fluorination and aldol reaction (αFAR) and its application to nucleoside synthesis. (D) Synthesis of 4′-thionucleosides from αFAR products. Blue colouring is used to emphasize the endocyclic sulfur atom and two key reactions: (i) αFAR, and (ii) annulative fluoride displacement (AFD).

For several decades nucleoside analogues (NAs) have served as a prolific source of antiviral and anticancer therapeutics, and account for more than half of all approved antiviral drugs.^1^ However, the pressure of new pathogens and emergence of drug resistance has highlighted the need for continued exploration of NA-relevant chemical space to identify compounds with novel mechanisms of action or enhanced resistance-combating properties.^2^ One promising class of NAs that have been studied since the 1960s^3^ are 4′-thio NAs (thNAs), where the ring oxygen is replaced with a sulfur atom. This single modification can have a profound impact on biological activity, including pharmacokinetic and pharmacodynamic properties.^1,4^ Further, due to the increased stability of the C–N anomeric bond, thNAs are generally more resistant to hydrolysis.^5^ For example, thiarabine (4′-thioaraC (1), Fig. 1),^4^ a thNA of the sponge metabolite cytarabine, was developed to treat hematological malignancies and solid tumors. Here, replacement of the endocyclic oxygen with sulfur resulted in an improved once daily oral dosing regimen compared to cytarabine, which requires twice-daily intravenous administration.^6^ The structurally related 2′-deoxyfluoro thNA FF-10502 (2)^7^ is an anticancer agent with improved potency over the related NA gemcitabine. Additionally, 4′thio-DMDC (3)^8^ and the C4′ alkyne thNA 4 ^9^ have demonstrated potent anticancer and anti-HIV activities, respectively. In particular, the C4′-alkyne containing thNA 4 is a nucleoside/nucleotide reverse transcriptase inhibitor (NRTI) that also demonstrated an excellent selectivity index.^9^ The use of thNAs in oligonucleotide sequences is also of importance, and processes to access 4′-thio locked nucleic acids (LNAs)^10^ or to carry out nucleobase diversification using biocatalysis^11^ have advanced efforts in this area.

A common approach to thNAs involves production of a protected 4-thioribose (*e.g.*, 6), which can be achieved in as little as 6 steps.^12^ For example, Miller has shown that 6 can be accessed from the ribose-derived bromo aldehyde 5 on multigram scale by bromide displacement using NaSH.^12^ This synthesis supported production of 4′-thiouridine (7),^12^ a precursor to thiarabine (1). An important contribution by Guindon^13^ demonstrated that thNAs can also be constructed using an acyclic approach where the nucleobase is attached prior to cyclization. For example, the ribose-derived ^*t*^Bu thioether 9 was cyclized under basic conditions to form the 2′-fluoro thNA 10.^13^

Our groups have previously reported^14^ a straightforward synthesis of NAs 14 that relies on two key steps: (i) a one-pot α-fluorination and aldol reaction (αFAR), and (ii) an annulative fluoride displacement (AFD) reaction (Fig. 1C). Considering the ease of access to ketofluorohydrins of general structure 13, and precedent for the formation of thioribose analogues *via* displacement strategies (*e.g.*, Fig. 1B), we sought to extend our NA synthesis platform to the preparation of thNAs. Importantly, this approach would afford orthogonally protected thNAs and should support the synthesis of C2′-modified thNAs (*e.g.*, 1 and 2). Here, we report the development of this process, its application to the synthesis of purine and pyrimidine thNAs and a 4′-seleno NA, and the multigram-scale synthesis of 5-methyl 4′-thiouridine.

## Results and discussion

**Scheme 1:**
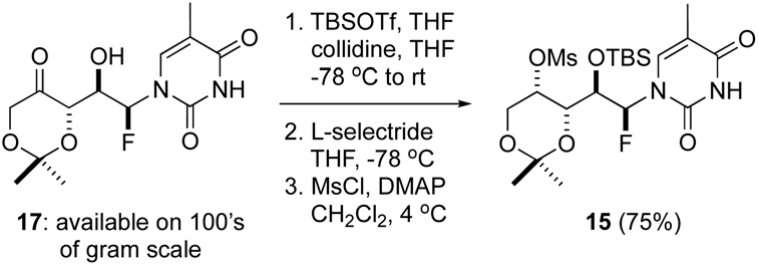
Synthesis of the fluoromesylate 15.

Our initial efforts focused on the use of thymine derivative 17, which was prepared on 100 g scale and is a stable solid that can be stored for months without notable degradation.^14^ We have shown that the direct reduction of the ketone function in 17 using Me_4_N·BH(OAc)_3_ affords 1,3-*syn* diols with high levels of diastereoselectivity.^14^ Application to thNA synthesis would require 1,3-*anti* selectivity in the reduction step owing to the planned invertive cyclization process (*i.e.*, S_N_2 reaction at C4′). We found this could be readily achieved by first protecting the secondary alcohol function as a TBS ether and subsequently reducing the carbonyl function with l-selectride.^15^ With the mono-TBS protected 1,3-*anti* diol in hand, several activation strategies were examined and ultimately mesylation proved to be optimal. Thus, the fluoro mesylate 15 could be reliably prepared in 3 steps from 17 in 75% overall yield following this straightforward process (Scheme 1).

We next explored the reaction of fluoromesylate 15 with various sulfur nucleophiles^15^ with an aim to effect a one-pot double displacement and gain direct access to thNA 16 or generate a masked thiolate group and intercept intermediates related to those described by Guindon^13^ (*e.g.*, 9, Fig. 1). Surprisingly, common thiol nucleophiles, including benzyl mercaptan (18), potassium thioacetate (19), NaSH (20) and Na_2_S·9H_2_O (21), did not react with mesylate 15 in DMF, even at 90 °C (Table 1, entries 1–4). Further heating of these reactions led to hydrolysis of the nucleobase and degradation. However, we were pleased to find that using freshly recrystallized Na_2_S·9H_2_O, the desired double displacement occurred readily at 90 °C, giving the thNA 16 in 50% yield (entry 5). Further optimization ultimately identified DMSO as the optimal solvent for this reaction and we found additionally that in DMSO, NaSH was an efficient sulfur nucleophile that reproducibly gave the thNA 16 in ∼60% yield. Thus, following this straightforward sequence, the αFAR product 17 could be converted into thNA 16 in four steps with an overall yield of ∼50%. Importantly, owing to the orthogonal protection of the secondary alcohol functions in 16, this route should support the synthesis of thNA functionalized at C2′ (see below).

**Table 1:** A double displacement reaction to access thNA 16

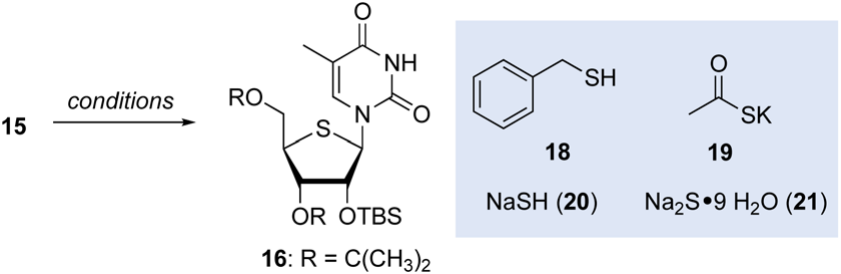				
Entry	Thiol	Solvent	Temp.	Yield (%)
1	18	DMF	90 °C	0
2	19	DMF	90 °C	0
3	20	DMF	90 °C	0
4	21	DMF	90 °C	0
5	21^a^	DMF	90 °C	50
6	20	DMSO	100 °C	61

a Freshly recrystalized 21 (Na_2_S).

**Fig. 2:**
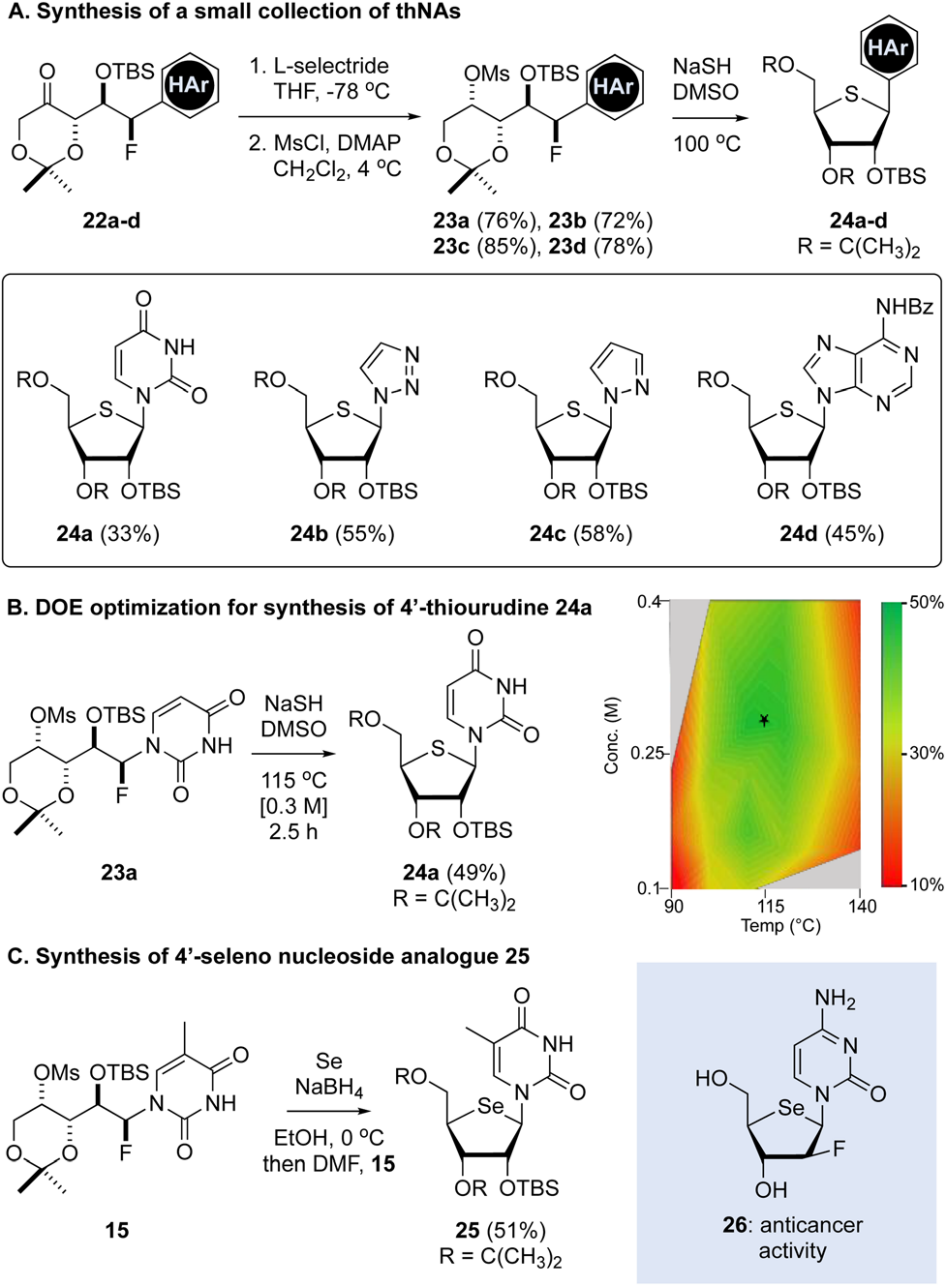
Synthesis of a purine, pyrimidine and other thNAs. (A) Examples of pyrimidine, purine and other C4′-thNAs produced from αFAR products. (B) Optimization of reaction temperature and concentration for the production of 24a. (C) Synthesis of the 4′-seleno nucleoside analogue 25.

Having established a route to the thNA 16, we next evaluated the scope of this thNA synthesis starting with the readily available TBS-protected fluorohydrins 22a–d (Fig. 2). The fluorohydrins can be prepared in 2 or 3 steps from the commercial nucleobase/heterocycle,^14,16^ though in the case of uracil- and triazole-containing fluorohydrins these were produced as inseparable mixtures of *syn*- and *anti*-fluorhydrins as described.^12^ These diastereomers were separable following TBS protection and mesylate formation (see ESI†). We further demonstrated that this process was compatible with pyrazole and benzoyl-protected adenosine, each of which gave the corresponding thNAs 24a–d in good overall yield. This reaction sequence was further optimized for execution with minimal chromatography and this sequence of steps could be executed as a through process with little impact on the overall yield. Due to challenges in accessing the corresponding cytosine and guanine aldol products (*e.g.*, 22, HAr = cytosine or guanine), synthesis of the corresponding thNAs was not explored.

Unfortunately, reaction of the uracil containing fluoro mesylate 23a with NaSH led to substantial cleavage of the uracil function and degradation, with uracil being released at a similar rate as thNA 24a formation. Thus, using the standard reaction conditions (Table 1, entry 6), the 4′-thiouridine 24a was produced in 33% yield. In an effort to improve on this result, we conducted Design of Experiment (DOE) optimization,^17^ focusing on the relationship between temperature, concentration, and time (Fig. 2B). Here, we found a correlation between concentration and time, with a maximum yield of 47% at a concentration of ∼0.3 M after 3 hours at 115 °C. Using these optimized conditions, the 4′-thiouridine 24a could be produced in 4 steps and ∼40% overall yield from the TBS-protected keto fluoride 22a. To demonstrate the versatility of this approach, we also reacted the thymine derivative 15 with NaSeH, generated in EtOH by the reduction of Se with NaBH_4_. As highlighted in Fig. 2C, this reaction gave the 4′-selenonucleoside analogue 25, which is an analogue of the known 4′-selenouridine.^18^ Notably, selenonucleosides (*e.g.*, 26 ^19^) have also attracted attention as anticancer agents.

**Fig. 3:**
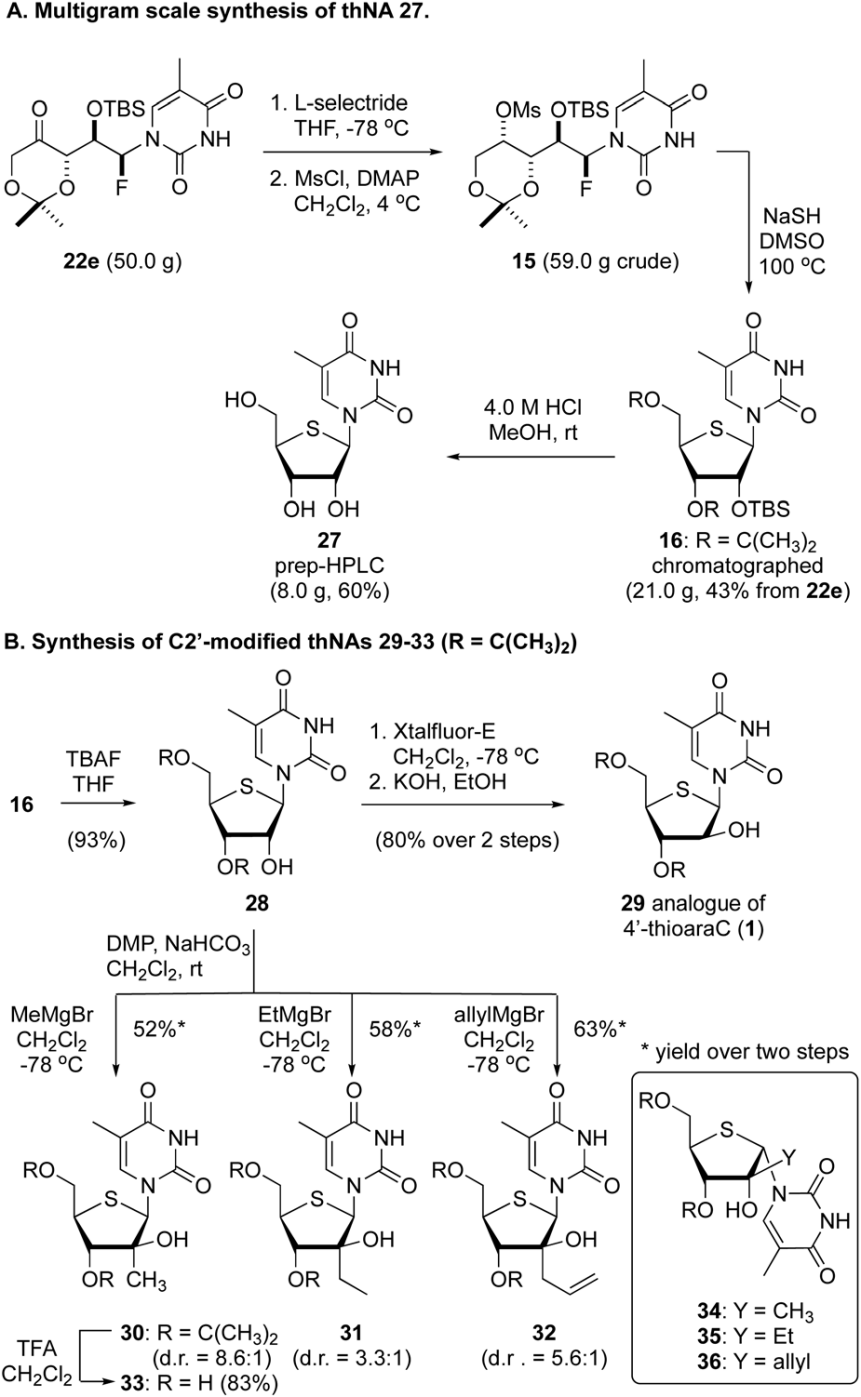
Large scale synthesis of thNA 27 and synthesis of C2′-modified thNAs 29–36. (A) A multigram scale synthesis of the thNAs 16 and 27. (B) Synthesis of C2′-modified thNAs.

To assess the scalability of this thNA synthesis, we additionally executed the process starting with 50.0 g of the protected αFAR product 22e (Fig. 3a). Without additional optimization we found that the sequence of reduction and mesylation proceeded in good overall yield, affording 59.0 g of the mesylate 15. From here, reaction with NaSH in DMSO at 100 °C gave 21.0 g of the thNA 16, which was purified by flash column chromatography. Removal of the silyl and acetonide protecting groups by treatment with 4 M HCl in MeOH then afforded 8.0 g of 4′-thio-5-methyluridine 27.^20^

Finally, considering that this process affords orthogonally protected thNAs (*e.g.*, 16), we investigated the selective C2′-functionalization of thymine thNA 16. As highlighted in Fig. 3B, removal of the TBS protecting group afforded the 2′-OH thNA 28 in excellent yield. From here, a 2-step sequence involving formation of the anhydro thNA and hydrolysis gave the arabino-configured thNA 29 in good overall yield. Notably, the corresponding triol (*i.e.*, deprotected) has demonstrated activity as low as 0.77 μg mL^−1^ against HSV-1.^21^ Additionally, despite concerns regarding anomerization of 2′-keto thNAs,^22^ we found that oxidation of compound 28 using Dess Martin periodinane buffered with NaHCO_3_ in CH_2_Cl_2_ gave clean conversion to the corresponding 2′-keto derivative. The use of NaHCO_3_ in this reaction proved critical, and several other standard oxidation conditions failed to provide the 2′-ketone in any reasonable yield. This latter material proved to be unstable on all stationary phases used for chromatographic purifications and thus the crude material was reacted directly with Grignard reagents to afford a small collection of previously unreported C2′-modified thNAs 30–36. In all cases, the arabino-configured stereoisomer was the major product, and the minor product was that derived from epimerization at C1′ prior to reaction with the Grignard reagent (*e.g.*, 34–36, see inset). Similar results have been reported by Matsuda.^22^ The use of more hindered Grignard reagents (*e.g.*, ^c^PrMgBr or ^i^PrMgBr) resulted in larger amounts of C1′-epimerization. Removal of the acetonide protecting group from 30 using TFA gave the corresponding triol 33 in excellent yield. Notably, Liotta has recently reported related, ribose-configured C2′-modified thNAs.^23^

## Conclusions

In summary, we report a streamlined process for the synthesis of various thNAs that exploits the ready availability of ketofluorohydrin aldol products. Importantly, the resulting thNAs are orthogonally protected, which enables synthesis of C2′-modified thNAs. This overall 7-step sequence was also demonstrated on multi-gram scale in the preparation of 5-methyl-4′-thiouridine (27) suggesting potential utility for larger scale, process research efforts. Importantly, the demonstration that purine and pyrimidine thNAs, 4′-seleno NAs, and C2′-modified thNAs can be readily prepared following straightforward strategies suggests that this new approach should inspire and support medicinal chemistry efforts in this area.

## Data availability

The experimental procedures, characterization data and ^1^H and ^13^C NMR spectroscopic data generated in this study are provided in the ESI.†

## Author contributions

R. B., C. L. and M. N. designed the study, R. B., C. L. and M. N. developed the synthetic plans and C. L., E. F., M. N. and B. S. optimized and executed the synthesis of all new compounds. S. S. and L.-C. C. supervised the large (multi-gram) scale reactions. R. B. and C. L. prepared the manuscript text. All authors contributed to the ESI.†

## Conflicts of interest

There are no conflicts to declare.

## Supplementary Material

SC-OLF-D4SC05679E-s001
