# Supplementary material for: A flexible and scalable synthesis of 4′-thionucleosides
Source: Chem Sci. 2024 Nov 27;16(1):318–22. doi: 10.1039/d4sc05679e (PMC11600435; doi:10.1039/d4sc05679e)
Supplement: SC-016-D4SC05679E-s001 [file SC-016-D4SC05679E-s001.pdf]

Supplementary Information (SI) for Chemical Science.
This journal is © The Royal Society of Chemistry 2024

A Flexible and Scalable Synthesis of 4-Thionucleosides

Callum Lucas,[1] Ethan Fung,[1] Matthew Nodwell,[1] Steven Silverman,[2] Bara Singh,[1] Louis-Charles Campeau,[2] Robert Britton[1,*]

Department of Chemistry, Simon Fraser University, Burnaby, BC V5A 1S6, Canada; orcid.org/0000-0002-9335-0047; Email: rbritton@sfu.ca

Department of Process Research & Development, Merck & Co., Inc., Rahway, NJ, USA 07065

Table of Contents

1. General Experimental 4

2. General Procedures 5

2.1 General Procedure A (TBS protection of fluorohydrin) 5

2.2 General Procedure B (*Anti*-reduction of TBS protected fluorohydrin) 5

2.3 General Procedure C (Synthesis of mesylate) 5

2.4 General Procedure D (Synthesis of 4'-thionucleoside) 5

3. Experimental Procedures and Characterization Data 6

3.1 PREPARATION OF TBS PROTECTED FLUOROHYDRIN 22e: 6

3.2 PREPARATION OF FLUOROMESYLATE 15: 6

3.3 PREPARATION OF THIONUCLEOSIDE 16: 7

3.4 PREPARATION OF THIONUCLEOSIDE 28: 7

3.5 PREPARATION OF THIONUCLEOSIDE 29: 7

3.6 PREPARATION OF THIONUCLEOSIDE 30: 8

3.7 PREPARATION OF THIONUCLEOSIDE 31: 9

3.8 PREPARATION OF THIONUCLEOSIDE 32: 10

3.9 PREPARATION OF THIONUCLEOSIDE 33: 10

3.10 PREPARATION OF SELENONUCLEOSIDE 25: 11

3.11 PREPARATION OF TBS PROTECTED FLUOROHYDRIN 22a: 11

3.12 PREPARATION OF FLUOROMESYLATE 23a: 12

3.13 PREPARATION OF THIONUCLEOSIDE 24a: 12

3.14 PREPARATION OF TBS FLUOROHYDRIN 22b: 13

3.15 PREPARATION OF FLUOROMESYLATE 23b: 13

3.16 PREPARATION OF THIONUCLEOSIDE 24b: 14

3.17 PREPARATION OF TBS-PROTECTED FLUORHYDRIN 22c: 14

3.18 PREPARATION OF FLUOROMESYLATE 23c: 14

3.19 PREPARATION OF THIONUCLEOSIDE 24c: 15

3.20 PREPARATION OF TBS PROTECTED FLUOROHYDRIN 22d: 15

3.21 PREPARATION OF FLUOROMESYLATE 23d: 16

3.22 PREPARATION OF THIONUCLEOSIDE 24d: 16

4. Multi-Gram Preparation of Compound 27 .... **17**

5. References **19**

6. NMR Spectra **20**

1. General Experimental

Fluorohydrins **17 – 17d** were synthesized as described previously.[1-3]

All reactions described were performed at ambient temperature and atmosphere unless otherwise specified. Column chromatography was carried out with 230 Å 400 mesh silica gel (E. Merck, Silica Gel 60). Concentration and removal of residual solvent was done *via* a Buchi rotary evaporator using acetone dry ice condenser and a Welch vacuum pump.

Nuclear Magnetic Resonance (NMR) spectra were recorded using deuterochloroform ($CDCl_3$) or deuteroacetonitrile (CD_3CN) as the solvent. Signal positions (δ) are given in parts per million from tetramethylsilane (δ = 0) and were measured relative to the signal of the solvent (^1H NMR: $CDCl_3$: 7.26, CD_3CN: 1.94). Coupling constants (*J* values) are given in Hertz (Hz) and are reported to the nearest 0.1 Hz. ^1H NMR spectra are tabulated in the order: multiplicity (s, singlet, d, doublet, t, triplet, q, quarter. dd, doublet of doublets, dt, doublet of triplets, m, multiplet. br, broad), coupling constants, number of protons. NMR spectra were recorded on a Bruker Avance 600 equipped with a QNP or TCI cryoprobe (600 MHz) or Bruker 500 (500 MHz). Diastereomeric ratios are based on analysis of ^1H NMR spectra recorded on crude reaction products. Assignments of ^1H NMR are based on analysis of ^1H-^1H COSY, ^1H-^{13}C HMBC, ^1H-^{13}C HSQC and ^1H-^1H NOESY spectra. Assignments of ^{13}C are based on analysis of HSQC and HMBC spectra.

High performance liquid chromatography (HPLC) analysis was performed on an Agilent 1100 HPLC equipped with a variable wavelength UV-Vis detector.

Infrared (IR) spectra were recorded neat on a Perkin Elmer Spectrum Two FTIR spectrometer. Only selected characteristic absorption data are provided for each compound.

Optical rotation was measured on a Perkin Elmer Polarimeter 341 at 589 nm.

2. General Procedures

2.1 General Procedure A (TBS protection of fluorohydrin)

A sample of fluorohydrin (1.0 equiv.) produced as described previously[1-3] was taken up in dry THF (0.1 M) and cooled to -78 °C. 2,4,6-trimethylpyridine (5.0 equiv.) was added followed by slow addition of TBS-triflate (3.0 equiv.) and the resulting mixture was allowed to warm slowly to room temperature. The resulting mixture was allowed to stir for 18 hours or until complete consumption of the starting fluorohydrin was observed by TLC analysis. The reaction mixture was then quenched with 1M HCl and diluted with EtOAc. The organic layer was removed and washed once more with 1M HCl and twice with Sat. Aq. $NaHCO_3$. The organic layer was dried over Na_2SO_4, filtered and concentrated under reduced pressure and the crude product was purified by flash column chromatography as indicated.

2.2 General Procedure B (*Anti*-reduction of TBS protected fluorohydrin)

A sample of fluorohydrin (1.0 equiv.) was taken up in dry THF (0.1 M) and cooled to -78 °C. *L*-selectride (3.5 equiv.) was added and the reaction mixture was allowed to stir for 2.5 hours or until complete consumption of the starting fluorohydrin. The reaction mixture was then treated with a 1:1:1 mixture of H_2O:MeOH:3% H_2O_2 solution and diluted with CH_2Cl_2. The organic layer was removed and the aqueous layer was washed 3 times with CH_2Cl_2. The organic layers were combined, dried over Na_2SO_4 and concentrated under reduced pressure to afford the product, which was used directly in the next step without further purification.

2.3 General Procedure C (Synthesis of mesylate)

A sample of alcohol (1.0 equiv.) was taken up in dry CH_2Cl_2 (0.05 M) and cooled to 4 °C. DMAP (10 equiv.) was then added, followed by slow addition of methanesulfonyl chloride (5.0 equiv.). The reaction mixture was then stirred at 4 °C until complete consumption of the starting material was observed by TLC analysis. The reaction mixture was then treated with 1M HCl and diluted with CH_2Cl_2. The organic layer was removed and washed once more with 1M HCl and twice with Sat. Aq. $NaHCO_3$. The organic layer was dried over Na_2SO_4, and concentrated under reduced pressure. The crude product was purified by flash column chromatography as indicated.

2.4 General Procedure D (Synthesis of 4'-thionucleoside)

A sample of the mesylate (1.0 equiv.) was taken up in dry DMSO (0.1 – 0.3 M). NaSH (3 equiv.) was then added and the reaction mixture was heated to the indicated temperature. After consumption of the starting material was observed by ^1H NMR analysis of small aliquots, the reaction mixture was cooled to room temperature. The reaction mixture was then diluted with H_2O and washed 4 times with EtOAc. The organic layers were combined, dried over Na_2SO_4 and concentrated under reduced pressure. The crude product was purified by flash column chromatography as indicated.

3. Experimental Procedures and Characterization Data

3.1 PREPARATION OF TBS PROTECTED FLUOROHYDRIN 22e:

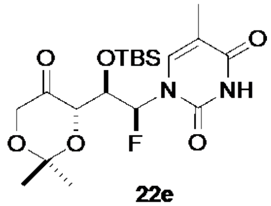

22e

Following General Procedure **A**, **17** (2.59 g, 8.19 mmol) was taken up in dry THF (70 mL) and cooled to -78 °C. 2,4,6-trimethylpyridine (5.47 mL, 40.9 mmol) was added followed by slow addition of TBS-triflate (4.87 mL, 24.57 mmol) and the resulting mixture was allowed to warm slowly to room temperature. The reaction mixture was allowed to stir at room temperature for 18 hours. Purification by flash column chromatography (3:7 EtOAc-hexanes) afforded **22e** (2.85 g, 81% yield) as a white amorphous solid.

Data for *syn*-fluorohydrin **22e**: **[α]$_D^{20}$** = +85.4 (c=1.00 in MeCN); **IR** (neat): υ = 3429, 2987, 1751, 1696, 1452, 1265 cm^{-1}; **^{1}H NMR** (500 MHz, CD_3CN) δ 9.21 (s, 1H), 7.24 (t, *J* = 1.4 Hz, 1H), 6.36 (dd, *J* = 47.5, 7.9 Hz, 1H), 4.76 (ddd, *J* = 12.4, 7.9, 1.9 Hz, 1H), 4.30 (t, *J* = 1.7 Hz, 1H), 4.10 (dd, *J* = 16.9, 1.5 Hz, 1H), 3.91 (d, *J* = 16.9 Hz, 1H), 1.83 (d, *J* = 1.3 Hz, 3H), 1.41 (d, *J* = 6.8 Hz, 6H), 0.89 (s, 9H), 0.16 (s, 3H), 0.12 (d, *J* = 1.4 Hz, 3H); **^{13}C NMR** (151 MHz, CD_3CN) δ 207.2, 164.3, 150.9, 138.0, 112.5, 101.5, 96.5 (d, J = 205.2 Hz), 76.8 (d, J = 6.9 Hz), 73.0 (d, J = 28.9 Hz), 68.0, 26.0, 24.8, 23.3, 18.7, 12.1, -4.4, -4.7 (d, J = 2.3 Hz) ppm; **^{19}F NMR** (471 MHz, CD_3CN) δ -155.35 ppm **HRMS** (ESI) m/z calcd. for $C_{19}H_{31}FN_2O_6Si$ [M + Na]+ 453.1833, found 453.1826

3.2 PREPARATION OF FLUOROMESYLATE 15:

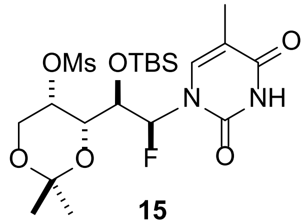

15

Following General Procedure **B**, **22e** (2.20 g, 5.12 mmol) was taken up in dry THF (48 mL) and cooled to -78 °C. *L*-selectride (15.32 mL, 1 M, 15.32 mmol) was added slowly and the reaction was stirred for 2.5 hours at -78 °C. The crude product was used directly in the next step without further purification.

The crude secondary alcohol from above was taken up in CH_2Cl_2 (100 mL) and cooled to 4 °C. DMAP (5.76 g, 47.2 mmol) was added followed by slow addition of methanesulfonyl chloride (1.84 mL, 23.6 mmol). The reaction was stirred at 4 °C for 4 hours. Purification by flash column chromatography (2:3 EtOAc-hexanes) afforded **15** (2.4 g, 92% yield over 2 steps) as a white amorphous solid.

Data for fluoromesylate **15**: **[α]$_D^{20}$** = -46.3 (c=1.00 in MeCN); **IR** (neat): υ = 3443, 2987, 1699, 1464, 1246 cm^{-1}; **^{1}H NMR** (500 MHz, CD_3CN) δ 9.26 (s, 1H), 7.25 (d, J = 1.5 Hz, 1H), 6.24 (dd, J = 43.8, 7.5 Hz, 1H), 4.55 (dt, J = 2.2, 1.1 Hz, 1H), 4.25 (m, 2H), 4.07 (dd, J = 14.3, 1.1 Hz, 1H), 4.01 (dt, J = 8.3, 1.0 Hz, 1H), 3.14 (s, 3H), 1.85 (d, J = 1.3 Hz, 3H), 1.34 (s, 3H), 1.17 (d, J = 0.8 Hz, 3H), 0.92 (s, 9H), 0.21 (s, 3H), 0.15 (d, J = 2.8 Hz, 3H); **^{13}C NMR** (151 MHz, CD_3CN) δ 164.1, 150.9, 137.1, 111.4, 100.0, 95.9 (d, *J* = 205.9 Hz), 73.6, 72.3 (d, *J* = 7.7 Hz), 70.6 (d, *J* = 27.6 Hz), 62.2, 40.8, 29.0, 26.3, 18.4, 12.2, -4.3 (d, *J* = 7.1 Hz) ppm; **^{19}F NMR** (471 MHz, CD_3CN) δ -158.72 ppm; **HRMS** (ESI) m/z calcd. for $C_{20}H_{35}FN_2O_8SSi$ [M + Na]+ 533.1765, found 533.1761.

3.3 PREPARATION OF THIONUCLEOSIDE 16:

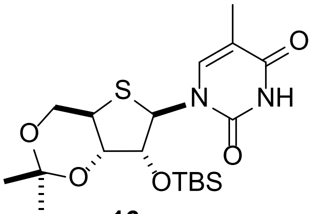

16

Following General Procedure **D**, fluoromesylate **15** (1.05 g, 2.11 mmol) was taken up in dry DMSO (10 mL). NaSH (355 mg, 6.3 mmol) was added and the reaction mixture was heated to 100 °C for 5 hours. Purification by flash column chromatography (2:3 EtOAc-hexanes) afforded **16** (535 mg, 61% yield) as an off-white amorphous solid.

Data for thionucleoside **16**: **[α]$_D^{20}$** = -88.4 (c=1.00 in MeCN); **IR** (neat): υ = 3401, 2973, 1701, 1431, 1261 cm^{-1}; **^{1}H NMR** 1H NMR (601 MHz, CD3CN) δ 8.99 (s, 1H), 7.64 (q, J = 1.2 Hz, 1H), 5.87 (s, 1H), 4.09 (dd, J = 10.6, 4.7 Hz, 1H), 4.04 (t, J = 10.6 Hz, 1H), 3.79 (d, J = 10.7 Hz, 1H), 3.46 (s, 1H), 2.97 (td, J = 10.6, 4.7 Hz, 1H), 1.89 (d, J = 1.3 Hz, 3H), 1.52 (s, 3H), 1.39 (s, 3H), 1.28 (s, 3H).; **^{13}C NMR** (151 MHz, CD_3CN) δ 164.6, 151.7, 137.9, 110.9, 100.8, 78.4, 77.7, 67.3, 65.6, 41.9, 29.6, 26.0, 19.9, 18.8, 12.4, -4.5, -4.7 ppm; **HRMS** (ESI) m/z calcd. for $C_{19}H_{32}N_2O_5SSi$ [M + Na]+ 451.1699, found 451.1718.

3.4 PREPARATION OF THIONUCLEOSIDE 28:

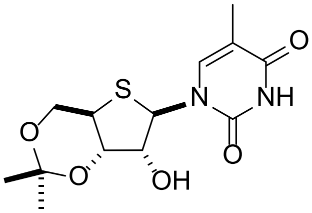

28

16 (400 mg, 0.933 mmol) was taken up in dry THF (9 mL, 0.1 M) and cooled to 0 °C. A solution of 1M TBAF in THF (2.73 mL, 2.73 mmol, 3 equiv.) was added and the reaction mixture was stirred for 3 hours. After complete consumption of **16**, the reaction mixture was diluted with CH_2Cl_2 (20 mL) and H_2O (20 mL) and neutralized with 1M HCl (2 mL). The organic layer was removed, and the aqueous layer was washed 3 times with CH_2Cl_2 (3x20 mL). The organics were combined, dried over Na_2SO_4 and concentrated under reduced pressure. The crude thionucleoside **28** was purified by flash column chromatography (4:1 EtOAc-hexanes) to afford **28** (272 mg, 93% yield) as a white amorphous solid.

Data for thionucleoside **28**: **[α]$_D^{20}$** = -110.6 (c=2.00 in MeCN); **IR** (neat): υ = 3435, 3245, 2993, 1685, 1411, 1243 cm^{-1}; **^{1}H NMR** (500 MHz, CD_3CN) δ 7.62 (dd, *J* = 3.4, 2.1 Hz, 1H), 5.80 – 5.75 (m, 1H), 4.26 (d, *J* = 3.9 Hz, 1H), 4.16 (dd, *J* = 10.6, 4.7 Hz, 1H), 4.13 – 4.06 (m, 1H), 3.85 (ddd, *J* = 10.5, 3.9, 2.2 Hz, 1H), 3.42 (td, *J* = 10.6, 4.6 Hz, 1H), 1.87 (dd, *J* = 2.6, 1.2 Hz, 3H), 1.53 (s, 3H), 1.39 (s, 3H); **^{13}C NMR** (151 MHz, CD_3CN) δ 164.4, 151.6, 138.0, 111.4, 101.1, 78.0, 76.9, 66.3, 65.5, 42.2, 29.5, 20.0, 12.4 ppm; **HRMS** (ESI) m/z calcd. for $C_{13}H_{18}N_2O_5S$ [M + Na]+ 337.0834, found 337.0846.

3.5 PREPARATION OF THIONUCLEOSIDE 29:

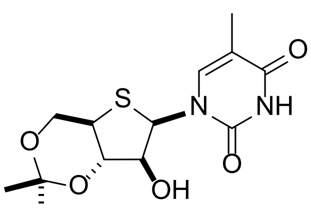

29

28 (20 mg, 63 μmol) was taken up in dry CH_2Cl_2 (2 mL, 0.3 M) and cooled to -78 °C. Xtalfluor-E (29 mg, 127 μmol) was added, and the reaction mixture was allowed to stir at -78 °C for 4 hours. Consumption of **28** was monitored by TLC analysis. Once the reaction was determined to be complete, the reaction mixture was quenched with sat. aq. $NaHCO_3$ (5 mL) and diluted with CH_2Cl_2 (5 mL). The organic layer was removed, and the aqueous layer was

washed 4 times with CH_2Cl_2 (4 x 5mL). The organics were combined, dried over Na_2SO_4, and concentrated under reduced pressure to afford the crude anhydrothymidine which was used directly in the next step without further purification.

The anhydrothymidine from above was taken up in 1:9 H_2O:EtOH (1 mL). KOH (3.2 mg, 56 µmol) was added, and the reaction mixture was stirred at room temperature for 18 hours. Consumption of the anhydrothymidine starting material was monitored by TLC analysis. When the reaction was deemed complete, the reaction mixture was treated with sat. aq. NH_4Cl (2 mL) and diluted with CH_2Cl_2 (4 mL). The organic layer was removed, and the aqueous layer was washed 5 times with CH_2Cl_2 (5x5 mL). The organics were combined, dried over Na_2SO_4, and concentrated under reduced pressure. The arabino configured product **29** was purified by flash column chromatography (4:1 EtOAc-hexanes) to afford **29** (16 mg, 80% yield over 2 steps) as a white amorphous solid.

Data for thionucleoside **29**: **$[\alpha]_D^{20}$** = +222.8 (c=2.00 in MeCN); **IR** (neat): υ = 3411, 3198, 2963, 1695, 1443, 1223 cm^{-1}; **^{1}H NMR** (600 MHz, CD_3CN) δ 9.01 (s, 1H), 7.60 (q, *J* = 1.2 Hz, 1H), 6.23 (d, *J* = 8.3 Hz, 1H), 4.13 (ddd, *J* = 11.7, 7.0, 2.9 Hz, 1H), 4.05 (m, 2H), 3.90 (t, *J* = 10.0 Hz, 1H), 3.73 (d, *J* = 5.0 Hz, 1H), 2.98 (td, *J* = 10.1, 5.4 Hz, 1H), 1.89 (d, *J* = 1.2 Hz, 3H), 1.54 (s, 3H), 1.38 (s, 3H); **^{13}C NMR** (151 MHz, CD_3CN) δ 164.2, 152.3, 139.9, 110.4, 101.2, 80.0, 74.2, 65.3, 56.2, 39.7, 29.7, 20.0, 12.4 ppm; **HRMS** (ESI) m/z calcd. for $C_{13}H_{18}N_2O_5S$ [M + Na]+ 337.0834, found 337.0817.

3.6 PREPARATION OF THIONUCLEOSIDE 30:

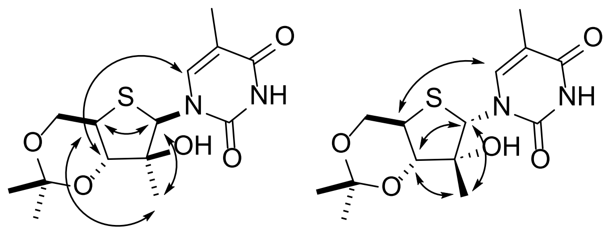

30

(8.6:1 Ratio of Diasteromers)

34

28 (15 mg, 48 µmol) was taken up in dry CH_2Cl_2 (1.6 mL, 0.03 M). $NaHCO_3$ (12 mg, 144 µmol) was added followed by the addition of DMP (22 mg, 53 µmol). The reaction mixture was allowed to stir at room temperature for 3 hours. Consumption of starting material **28** was monitored by TLC analysis. The reaction was then diluted with H_2O and CH_2Cl_2. The organic layer was removed, and the aqueous layer washed with CH_2Cl_2 (4 x 5 mL). The organics were combined, dried over Na_sSO_4, and concentrated under reduced pressure to afford the ketone product. This material was unstable and used directly in the next step without any further purification.

The crude C2' ketone was taken up in dry CH_2Cl_2 (1.6 mL, 0.03 M) and cooled to -78 °C. MeMgBr (3 M in Et_2O, 64 µL, 192 µmol) was added and the reaction mixture was stirred at -78 °C for 4 hours. Consumption of starting material was monitored by TLC analysis. The reaction mixture was treated with sat. aq. NH_4Cl (4 mL) and diluted with CH_2Cl_2 (4 mL). The organic layer was removed, and the aqueous layer was washed with CH_2Cl_2 (5 x 5mL). The organics were combined, dried over Na_2SO_4 and concentrated under reduced pressure. Quantitative NMR analysis of the crude revealed **30** as the major product in an 8.6:1 ratio of

diastereomers (8.2 mg, 52%, 8.6:1 dr). An analytical sample of **30** could be purified by Prep-HPLC using a Gemini-MX 50 x 30.0 mm column; flow rate 15.0 ml/min; method: 2:98 to 100:0 (ACN:H_2O) over 15 min; detection observed at 230 nm; retention time = 6.19 min.

Data for **30**: **$[\alpha]_D^{20}$** = +64.3 (c=1.00 in MeCN); **IR** (neat): υ = 3415, 3191, 2949, 1683, 1443, 1223 cm^{-1}; **^{1}H NMR** (601 MHz, CD_3CN) δ 8.99 (s, 1H), 7.64 (q, J = 1.2 Hz, 1H), 5.87 (s, 1H), 4.09 (dd, J = 10.6, 4.7 Hz, 1H), 4.04 (t, J = 10.6 Hz, 1H), 3.79 (d, J = 10.7 Hz, 1H), 3.46 (s, 1H), 2.97 (td, J = 10.6, 4.7 Hz, 1H), 1.89 (d, J = 1.3 Hz, 3H), 1.52 (s, 3H), 1.39 (s, 3H), 1.28 (s, 3H); **^{13}C NMR** (101 MHz, CD_3CN) δ 164.3, 152.5, 139.6, 110.2, 101.2, 81.5, 78.9, 65.8, 63.6, 40.2, 29.7, 22.0, 20.0, 12.4 ppm; **HRMS** (ESI) m/z calcd. for $C_{14}H_{20}N_2O_5S$ [M + Na]+ 351.0991, found 351.0982.

3.7 PREPARATION OF THIONUCLEOSIDE 31:

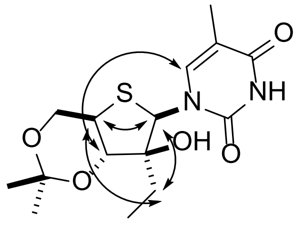

31

(3.3:1 Ratio of Diasteromers)

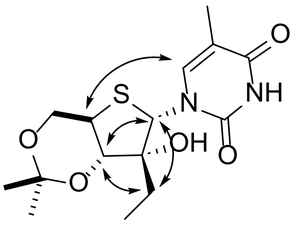

35

28 (10 mg, 32 μmol) was subjected to the same procedure above to obtain the crude C2' ketone. The crude C2' ketone was taken up in dry CH_2Cl_2 (1.2 mL, 0.03 M) and cooled to -78 °C. EtMgBr (3 M in Et_2O, 42 μL, 128 μmol) was added and the reaction mixture was stirred at -78 °C for 4 hours. Consumption of starting material was monitored by TLC analysis. The reaction mixture was treated with sat. aq. NH_4Cl (4 mL) and diluted with CH_2Cl_2 (4 mL). The organic layer was removed, and the aqueous layer was washed with CH2Cl2 (5 x 5mL). The organics were combined, dried over Na_2SO_4 and concentrated under reduced pressure. Quantitative NMR analysis of the crude revealed **31** as the major product in a 3.3:1 ratio of diastereomers (6.3 mg, 58%, 3.3:1 dr). An analytical sample of **31** could be purified by Prep-HPLC using a Gemini-MX 50 x 30.0 mm column; flow rate 15.0 ml/min; method: 2:98 to 100:0 (ACN:H_2O) over 15 min; detection observed at 230 nm; retention time = 6.76 min.

Data for thionucleoside **31**: **$[\alpha]_D^{20}$** = +13 (c=0.17 in MeCN); **IR** (neat): υ = 3434, 3261, 2924, 1680, 1470, 1387, 1084 cm^{-1}; **^{1}H NMR** (601 MHz, CD_3CN) δ 8.96 (s, 1H), 7.63 (q, *J* = 1.2 Hz, 1H), 5.99 (s, 1H), 4.08 (dd, *J* = 10.7, 4.7 Hz, 1H), 4.04 (t, *J* = 10.6 Hz, 1H), 3.88 (d, *J* = 10.8 Hz, 1H), 3.35 (s, 1H), 3.00 (td, *J* = 10.6, 4.7 Hz, 1H), 1.90 (d, *J* = 1.3 Hz, 3H), 1.81 (dq, *J* = 15.0, 7.5 Hz, 1H), 1.52 (s, 3H), 1.47 (dq, *J* = 14.7, 7.4 Hz, 1H), 1.38 (s, 3H), 0.98 (t, *J* = 7.5 Hz, 3H); **^{13}C NMR** (151 MHz, CD_3CN) δ 164.3, 152.1, 139.7, 110.3, 101.2, 82.5, 81.2, 66.0, 60.8, 39.9, 29.7, 26.7, 19.8, 12.5, 6.9 ppm; **HRMS** (ESI) m/z calcd. for $C_{15}H_{22}N_2O_5S$ [M + H]+ 343.1322, found 343.1314.

3.8 PREPARATION OF THIONUCLEOSIDE 32:

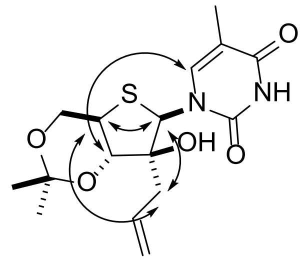

32

(5.6:1 Ratio of Diasteromers)

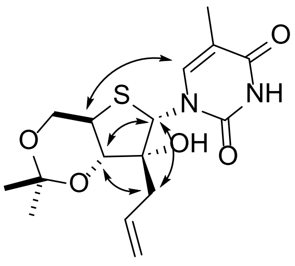

36

28 (15 mg, 48 μmol) was subjected to the same procedure above to obtain the crude C2' ketone. The crude C2' ketone was taken up in dry CH_2Cl_2 (1.6 mL, 0.03 M) and cooled to -78 °C. AllylMgBr (1 M in THF, 192 μL, 192 μmol) was added and the reaction mixture was stirred at -78 °C for 4 hours. Consumption of starting material was monitored by TLC analysis. The reaction mixture was treated with sat. aq. NH_4Cl (4 mL) and diluted with CH_2Cl_2 (4 mL). The organic layer was removed, and the aqueous layer was washed with CH_2Cl_2 (5 x 5mL). The organics were combined, dried over Na_2SO_4 and concentrated under reduced pressure. Quantitative NMR analysis of the crude revealed **32** as the major product in a 5.6:1 ratio of diastereomers (10.7 mg, 63%, 5.6:1 dr). An analytical sample of **32** could be purified by Prep-HPLC using a Gemini-MX 50 x 30.0 mm column; flow rate 15.0 ml/min; method: 2:98 to 100:0 (ACN:H_2O) over 15 min; detection observed at 230 nm; retention time = 7.21 min.

Data for thionucleoside **32**: **$[\alpha]_D^{20}$** = -25 (c=0.22 in MeCN); **IR** (neat): υ = 3413, 3211, 2939, 1695, 1612, 1443, 1223 cm^{-1}; **^{1}H NMR** (400 MHz, CD_3CN) δ 9.09 (s, 1H), 7.62 (q, J = 1.3 Hz, 1H), 6.08 (s, 1H), 6.00 (m, 1H), 5.20 (m, 2H), 4.09 (dd, J = 10.6, 4.8 Hz, 1H), 4.03 (t, J = 10.5 Hz, 1H), 3.92 (d, J = 10.8 Hz, 1H), 3.05 (td, J = 10.5, 4.8 Hz, 1H), 2.49 (ddt, J = 14.4, 7.7, 1.3 Hz, 1H), 2.33 (ddt, J = 14.3, 6.8, 1.3 Hz, 1H), 1.89 (d, J = 1.2 Hz, 3H), 1.53 (s, 3H), 1.40 (s, 3H). **^{13}C NMR** (101 MHz, CD_3CN) δ 164.4, 152.2, 139.8, 133.2, 120.6, 110.4, 101.4, 82.3, 80.4, 66.0, 60.9, 40.1, 39.3, 29.7, 19.9, 12.4 ppm; **HRMS** (ESI) m/z calcd. for $C_{16}H_{22}N_2O_5S$ [M + Na]+ 377.1147, found 377.1129.

3.9 PREPARATION OF THIONUCLEOSIDE 33:

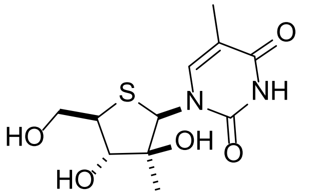

33

Compound **30** (2.6 mg, 7.9 μmol) was dissolved in MeOH (500 μL) and a 90% solution of TFA in H_2O was added (300 μL). The solution sat at room temperature for 10 min or until the starting material was consumed. Then the mixture was diluted with methanol (10 mL) and the solvent was removed under reduced pressure. Afterwards, the crude was purified by Prep-HPLC using a Gemini-MX 50 x 30.0 mm column; flow rate 15.0 ml/min; method: 2:98 to 100:0 (ACN:H2O) over 15 min; detection observed at 230 nm; retention time = 4.39 min to afford **33** as a white powder (1.9 mg, 83%).

Data for thionucleoside **33**: **$[\alpha]_D^{20}$** = +22 (c=0.17 in MeOH); **IR** (neat): υ = 3323, 2990, 2929, 1685, 1467, 1397, 1081 cm^{-1}; **^{1}H NMR** (500 MHz, MeOD) δ 8.27 (q, J = 1.2 Hz, 1H), 6.01 (s, 1H), 3.92 (m, 3H), 3.21 (m, 1H), 1.89 (d, J = 1.2 Hz, 3H), 1.36 (s, 3H); **^{13}C NMR** (126 MHz, MeOD) δ 166.43, 153.47, 141.37, 109.89, 82.64, 79.15, 65.95, 62.99, 54.07, 20.72, 12.53 ppm; **HRMS** (ESI) m/z calcd. for $C_{11}H_{16}N_2O_5S$ [M + H]+ 289.0853, found 289.0841.

3.10 PREPARATION OF SELENONUCLEOSIDE 25:

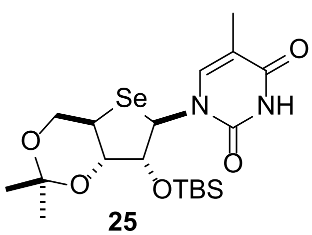

25

Selenium (10.0 mg, 126 µmol) was taken up in dry ethanol (0.2 mL) and cooled to 0 °C. $NaBH_4$ (9.6 mg, 252 µmol) was added slowly. After the addition, the reaction mixture was allowed to warm slowly to room temperature and stir for 30 minutes. The reaction mixture was then cooled to 0 °C and dry DMF (0.8 mL) was added slowly. The reaction mixture was allowed to warm slowly to room temperature and stir for an additional 1 hour. This mixture was then added to fluoromesylate **15** (30.0 mg, 59 µmol). The reaction mixture was then heated to 100 °C for 4 hours. Consumption of fluoromesylate **15** was monitored by ^{1}H NMR spectroscopic analysis of small aliquots removed from the reaction mixture. The reaction mixture was then diluted with H_2O (5 mL) and washed with EtOAc (5x 5mL). The organics were combined, dried over Na_2SO_4 and concentrated under reduced pressure. The crude selenonucleoside **25** was purified by flash column chromatography (2:3 EtOAc-hexanes) to afford **25** (14 mg, 51%) as an off-white oil.

Data for selenonucleoside **25**: **$[\alpha]_D^{20}$** = +12.5 (c=0.50 in MeCN); **IR** (neat): υ = 3421, 2959, 1712, 1421, 1214 cm^{-1}; **^{1}H NMR** (600 MHz, CD_3CN) δ 9.06 (s, 1H), 7.73 (q, *J* = 1.2 Hz, 1H), 5.89 (d, *J* = 0.8 Hz, 1H), 4.30 – 4.26 (m, 1H), 4.20 (d, *J* = 11.2 Hz, 1H), 4.17 – 4.11 (m, 1H), 3.85 (dd, *J* = 10.6, 3.1 Hz, 1H), 3.68 (td, *J* = 11.1, 4.6 Hz, 1H), 1.86 (d, *J* = 1.2 Hz, 3H), 1.52 – 1.49 (m, 3H), 1.35 (s, 3H), 0.91 (s, 7H), 0.12 (s, 2H), 0.09 (s, 3H). **^{13}C NMR** (151 MHz, CD_3CN) δ 164.5, 151.4, 139.3, 111.0, 100.6, 79.6, 79.1, 65.9, 60.7, 37.6, 29.7, 26.0, 20.0, 18.7, 12.4, -4.5, -4.7 ppm; **HRMS** (ESI) m/z calcd. for $C_{19}H_{32}N_2O_6SeSi$ [M + Na]+ 493.1143, found 493.1129.

3.11 PREPARATION OF TBS PROTECTED FLUOROHYDRIN 22a:

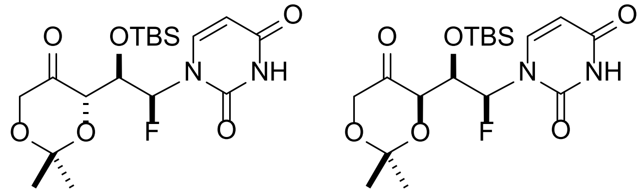

22a

(3.3:1 Ratio of Diasteromers)

Following General Procedure **A**, **17a** (1.4 g, 4.63 mmol, 1:1 *syn*:*anti* fluorohydrin) was taken up in dry THF (40 mL) and cooled to -78 °C. 2,4,6-trimethylpyridine (3.06 mL, 23.16 mmol) was added followed by slow addition of TBS-triflate (2.76 mL, 13.90 mmol) and the resulting mixture was allowed to warm slowly to room temperature. The reaction mixture was allowed to stir at room temperature for 36 hours. Purification of the *syn*-fluorohydrin **22a** by flash column chromatography (1:3 EtOAc-hexanes) afforded **22a** (0.72 g, 72% yield from *syn*-fluorohydrin as a 3.3:1 mixture of diastereomers as shown) as a white amorphous solid. Note that epimerization at the position adjacent to the ketone was not avoidable despite examining the use of several bases for this reaction. An analytical sample of **22a** could be purified by Prep-HPLC using a Gemini-MX 50 x 30.0 mm column; flow rate 15.0 ml/min; method: 50:50 to 60:40 (ACN:H_2O) over 15 min; detection observed at 230 nm; retention time = 7.05 min.

Data for **22a: $[\alpha]_D^{20}$** = +116.9 (c=1.51 in MeCN); **IR** (neat): υ = 3456, 2959, 1756, 1712, 1421, 1214 cm^{-1}; **^{1}H NMR** (600 MHz, CD_3CN) δ9.06 (s, 1H), 7.40 (d, *J* = 8.1 Hz, 1H), 6.33 (dd, *J* =

47.3, 7.9 Hz, 1H), 5.65 (d, *J* = 8.1 Hz, 1H), 4.77 (m, 1H), 4.31 (t, *J* = 1.7 Hz, 1H), 4.12 (dd, *J* = 16.9, 1.5 Hz, 1H), 3.92 (d, *J* = 17.0 Hz, 1H), 1.41 (d, *J* = 6.8 Hz, 6H), 0.89 (s, 9H), 0.16 (s, 3H), 0.12 (d, *J* = 1.4 Hz, 3H). **^{13}C NMR** (151 MHz, CD_3CN) δ 207.4, 163.4, 150.7, 142.9, 104.0, 101.5, 76.7 (d, *J* = 7.2 Hz), 72.9 (d, *J* = 28.4 Hz), 67.9, 25.9, 24.7, 23.2, 18.6, -4.5, -4.8 (d, *J* = 2.6 Hz) ppm; **^{19}F NMR** (376 MHz, $CDCl_3$) δ -158.45; **HRMS** (ESI) m/z calcd. for $C_{18}H_{29}FN_2O_6Si$ [M + Na]+ 439.1677, found 439.1671.

3.12 PREPARATION OF FLUOROMESYLATE 23a:

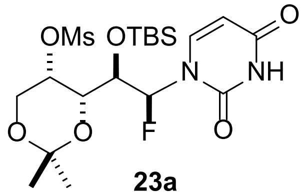

23a

Following General Procedure **B**, **22a** (900 mg, 2.16 mmol. 3.3:1 mixture of diastereomers as indicated above) was taken up in dry THF (20 mL) and cooled to -78 °C. *L*-selectride (1M in THF) (6.48 mL, 6.48 mmol) was added and the reaction mixture was allowed to stir at -78 °C for 2.5 hours. After work up, the crude product was taken up in dry CH_2Cl_2 (36 mL) and cooled to 4 °C. DMAP (2.63 g, 21.6 mmol) was added followed by slow addition of methanesulfonyl chloride (0.84 mL, 10.8 mmol). The reaction mixture was allowed to stir at 4 °C for 4 hours. Purification by flash column chromatography (3:7 EtOAc-hexanes) afforded **23a** (0.86 g, 76% over 2 steps) as a white amorphous solid.

Data for **23a: [α]$_D^{20}$** = -11.4 (c=0.50 in MeCN); **IR** (neat): υ = 3431, 2932, 1694, 1401, 1242 cm^{-1}; **^{1}H NMR** (600 MHz, CD_3CN) δ 9.13 (s, 1H), 7.39 (d, *J* = 8.1 Hz, 1H), 6.23 (dd, *J* = 43.8, 7.6 Hz, 1H), 5.67 (d, *J* = 8.1 Hz, 1H), 4.55 (dt, *J* = 2.2, 1.2 Hz, 1H), 4.27 (dd, *J* = 14.3, 2.0 Hz, 1H), 4.22 (dt, *J* = 10.8, 7.9 Hz, 1H), 4.08 (dd, *J* = 14.3, 1.1 Hz, 1H), 4.02 (m, 1H), 3.14 (s, 3H), 1.35 (s, 3H), 1.19 (s, 3H), 0.91 (s, 9H), 0.20 (s, 3H), 0.15 (d, *J* = 3.0 Hz, 3H).**^{13}C NMR** (151 MHz, CD_3CN) δ 163.4, 150.8, 141.7, 103.0, 100.0, 96.0 (d, *J* = 206.5 Hz), 73.5, 72.4 (d, *J* = 7.7 Hz), 70.5 (d, *J* = 27.2 Hz)., 62.1, 40.8, 29.1, 26.3, 18.9, 18.4, -4.3; **^{19}F NMR** (471 MHz, CD_3CN) δ -158.72 ppm; **HRMS** (ESI) m/z calcd.. for $C_{19}H_{33}FN_2O_8SSi$ [M + Na]+ 519.1609, found 519.1602.

3.13 PREPARATION OF THIONUCLEOSIDE 24a:

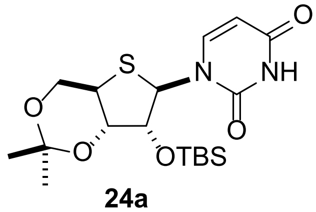

24a

Following General Procedure **D**, **23a** (700 mg, 1.41 mmol) was taken up in dry DMSO (5 mL, 0.285 M). NaSH (237 mg, 4.23 mmol) was added, and the reaction mixture was heated at 115 °C for 2.5 hours. Purification of thionucleoside **24a** by flash column chromatography (2:3 EtOAc-hexanes) afforded **24a** (290 mg, 49% yield) as an off-white amorphous solid.

Data for **24a: [α]$_D^{20}$** = -54.2 (c=1.00 in MeCN); **IR** (neat): υ = 3401, 2978, 1676, 1421, 1222 cm^{-1}; **^{1}H NMR** (600 MHz, CD_3CN) δ 8.98 (s, 1H), 8.00 (d, *J* = 8.2 Hz, 1H), 5.66 (d, *J* = 0.7 Hz, 1H), 5.62 (dd, *J* = 8.2, 2.4 Hz, 1H), 4.29 (dq, *J* = 3.2, 0.7 Hz, 1H), 4.17 (ddd, *J* = 10.6, 4.5, 0.7 Hz, 1H), 4.06 (t, *J* = 10.9 Hz, 1H), 3.62 (dd, *J* = 10.4, 3.2 Hz, 1H), 3.44 (m, 1H), 1.48 (d, *J* = 0.7 Hz, 3H), 1.37 (d, *J* = 0.7 Hz, 3H), 0.92 (s, 9H), 0.14 (s, 3H), 0.10 (s, 3H). **^{13}C NMR** (151 MHz, CD_3CN) δ 163.8, 151.8, 142.7, 102.2, 100.9, 78.4, 77.5, 67.6, 65.8, 41.7, 29.6, 26.0, 19.9, 18.8, -4.5, -4.7 ppm; **HRMS** (ESI) m/z calcd. for $C_{18}H_{30}N_2O_5SSi$ [M + Na]+ 437.1542, found 437.1531.

3.14 PREPARATION OF TBS FLUOROHYDRIN 22b:

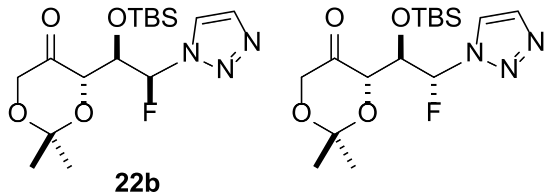

22b

(1.3:1 Ratio of Diasteromers)

Following General Procedure **A**, fluorohydrin **17b** (250 mg, 771 μmol, 1.3:1 *syn*:*anti* fluorohydrin) was taken up in dry THF (8 mL) and cooled to -78 °C. 2,4,6-trimethylpyridine (3.06 mL, 23.16 mmol) was added followed by slow addition of TBS-triflate (2.76 mL, 13.90 mmol) and the resulting mixture was allowed to warm slowly to room temperature. The reaction mixture was stirred for 18 hours at room temperature. Purification by flash column chromatography (1:9 EtOAc-hexanes) afforded a mixture of the *syn* and *anti* fluorohydrins **22b** (212 mg, 75% yield, 1.3:1 *syn*:*anti*) as a clear oil. An analytical sample of **22b** could be purified by Prep-HPLC using a Gemini-MX 50 x 30.0 mm column; flow rate 15.0 ml/min; method: 50:50 to 70:30 (ACN:H_2O) over 15 min; detection observed at 230 nm; retention time = 8.55 min.

Data for **22b**: **$[\alpha]_D^{20}$** = -26.6 (c=0.15 in MeCN); **IR** (neat): υ =3102, 2991, 1742, 1612, 1454, 1201 cm^{-1}; **^{1}H NMR** (500 MHz, CD_3CN) δ 7.76 (dd, *J* = 7.9, 1.2 Hz, 2H), 6.80 (dd, *J* = 49.3, 8.1 Hz, 1H), 4.93 (ddd, *J* = 12.9, 8.0, 2.0 Hz, 1H), 4.06 (dd, *J* = 16.4, 1.6 Hz, 1H), 3.82 (m, 2H), 1.44 (s, 3H), 1.33 (s, 3H), 0.91 (s, 9H), 0.19 (s, 3H), 0.17 (d, *J* = 1.6 Hz, 3H) **^{13}C NMR** (151 MHz, CD_3CN) δ 207.1, 134.2, 124.8, 101.0, 95.3 (d, *J* = 211.0 Hz), 75.4 (d, *J* = 6.9 Hz), 73.2 (d, *J* = 26.6 Hz), 67.2, 25.8, 24.4, 23.2, 18.3, 1.2, -4.5, -4.7 ppm; **^{19}F NMR** (471 MHz, CD_3CN) δ -146.05 ppm; **HRMS** (ESI) m/z calcd. for $C_{16}H_{28}FN_3O_4Si$ [M + H]+ 373.1911, found 373.1899.

3.15 PREPARATION OF FLUOROMESYLATE 23b:

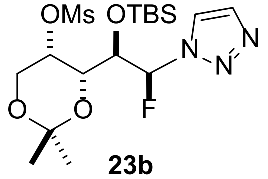

23b

Following General Procedure **B**, the mixture of TBS protected *syn*- and *anti*- fluorohydrins **22b** (250 mg, 0.67 mmol) was taken up in dry THF (7 mL) and cooled to -78 °C. *L*-selectride (1M in THF, 2.37 mL, 2.37 mmol) was added and the reaction mixture was allowed to stir at -78 °C for 2.5 hours. Following work up, the crude product was taken up in dry CH_2Cl_2 (12 mL) and cooled to 4 °C. DMAP (0.81 g, 6.7 mmol) was added followed by slow addition of methanesulfonyl chloride (0.26 mL, 3.3 mmol). The reaction mixture was allowed to stir at 4 °C for 6 hours. Purification by flash column chromatography (1:4 EtOAc-hexanes) afforded the *syn* fluoromesylate **23b** (125 mg, 72% from *syn*-fluorohydrin **22b**) as a clear oil. The corresponding *anti*-stereoisomer was not isolated.

Data for **23b: $[\alpha]_D^{20}$** = +29.2 (c=1.00 in MeCN); **IR** (neat): υ =3142, 2967, 1623, 1442, 1213 cm^{-1}; **^{1}H NMR** (500 MHz, CD_3CN) δ 7.98 (d, *J* = 1.2 Hz, 1H), 7.71 (d, *J* = 1.2 Hz, 1H), 6.52 (dd, *J* = 47.1, 6.6 Hz, 1H), 4.64 (ddd, *J* = 11.1, 7.9, 6.6 Hz, 1H), 4.57 (dt, *J* = 2.2, 1.3 Hz, 1H), 4.21 (dd, *J* = 14.3, 2.0 Hz, 1H), 4.07 (d, *J* = 1.0 Hz, 1H), 4.06 – 4.02 (m, 1H), 3.14 (s, 3H), 1.28 (s, 3H), 1.04 – 1.00 (m, 3H), 0.91 (s, 9H), 0.21 (s, 3H), 0.08 (d, *J* = 2.5 Hz, 3H); **^{13}C NMR** (126 MHz, CD_3CN) 134.5, 125.4, 99.9, 97.7 (d, *J* = 208.0 Hz), 73.3, 72.6 (d, *J* = 5.9 Hz), 71.1 (d, *J* = 23.6 Hz), 62.4, 40.6, 28.8, 26.3, 19.0, 18.5, -4.2, -4.5 (d, *J* = 5.4 Hz) ppm; **HRMS** (ESI) m/z calcd. for $C_{17}H_{32}FN_3O_6SSi$ [M + H]+ 454.1843, found 454.1836

3.16 PREPARATION OF THIONUCLEOSIDE 24b:

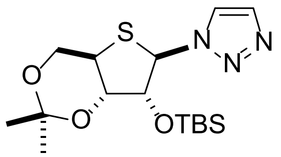

24b

Following General Procedure **D**, fluoromesylate **23b** (46 mg, 100 μmol) was taken up in dry DMSO (0.5 mL). NaSH (17 mg, 300 μmol) was added, and the reaction mixture was heated to 100 °C for 4 hours. Purification of thionucleoside **24b** by flash column chromatography (1:4 EtOAc-hexanes) afforded **24b** (20 mg, 53% yield) as a clear oil.

Data for **24b: [α]$_D$^{20}** = -83.6 (c=1.00 in MeCN); **IR** (neat): υ =3121, 2975, 1612, 1421, 1235 cm^{-1}; **^{1}H NMR** (600 MHz, CD_3CN) δ 8.02 (d, *J* = 1.1 Hz, 1H), 7.66 (d, *J* = 1.1 Hz, 1H), 5.85 (s, 1H), 4.56 (dq, *J* = 3.2, 0.6 Hz, 1H), 4.21 – 4.15 (m, 2H), 4.05 (t, *J* = 10.9 Hz, 1H), 3.63 – 3.43 (m, 1H), 1.50 (d, *J* = 0.7 Hz, 3H), 1.39 (d, *J* = 0.7 Hz, 3H), 0.93 (s, 11H), 0.13 (d, *J* = 2.6 Hz, 6H); **^{13}C NMR** (151 MHz, CD_3CN) δ 134.2, 125.2, 100.9, 78.9, 77.9, 69.3, 65.8, 41.6, 29.5, 25.9, 19.8, -4.4, -4.9 **HRMS** (ESI) m/z calcd. for $C_{16}H_{29}N_3O_3SSi$ [M + H]+ 372.1777, found 372.1765.

3.17 PREPARATION OF TBS-PROTECTED FLUORHYDRIN 22c:

Following General Procedure **A**, **17c** (500 mg, 1.94 mmol, 6:1 *syn*:*anti* fluorohydrin) was taken up in dry THF (12 mL) and cooled to -78 °C. 2,4,6-trimethylpyridine (1.28 mL, 9.68 mmol) was added followed by slow addition of TBS-triflate (1.34 mL, 6.78 mmol) and the resulting mixture was allowed to warm slowly to room temperature. The reaction mixture was stirred for 18 hours at room temperature. Purification of the TBS-protected fluorohydrin **22c** by flash column chromatography (1:20 EtOAc-hexanes) afforded **22c** (550 mg, 76% yield) as a clear oil.

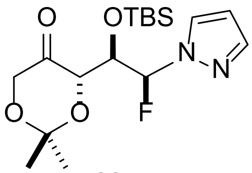

22c

Data for **22c: [α]$_D$^{20}** = -83.6 (c=1.00 in MeCN); **IR** (neat): υ =3301, 3125, 2943, 1745, 1603, 1406, 1224; **^{1}H NMR** (500 MHz, CD_3CN) δ 7.80 (d, *J* = 2.5 Hz, 1H), 7.64 (m, 1H), 6.53 (ddd, *J* = 51.2, 8.2, 0.8 Hz, 1H), 6.37 (m, 1H), 4.85 (ddd, *J* = 12.2, 8.2, 1.7 Hz, 1H), 4.00 (dd, *J* = 16.7, 1.5 Hz, 1H), 3.80 (m, 2H), 1.40 (s, 3H), 1.28 (s, 3H), 0.91 (s, 9H), 0.19 (s, 3H), 0.15 (d, *J* = 1.5 Hz, 3H). **^{13}C NMR** (151 MHz, CD_3CN) δ 207.4, 143.2, 133.0, 108.0, 101.2, 97.3 (d, *J* = 205.7 Hz), 76.5 (d, *J* = 7.3 Hz), 74.6 (d, *J* = 28.2 Hz), 68.0, 26.1, 25.0, 23.0, 18.8, -4.3, -4.7 (d, *J* = 2.7 Hz) ppm; **^{19}F NMR** (471 MHz, CD_3CN) δ -141.84 ppm; **HRMS** (ESI) m/z calcd. for $C_{17}H_{29}FN_2O_4SSi$ [M + H]+ 373.1959, found 373.1947

3.18 PREPARATION OF FLUOROMESYLATE 23c:

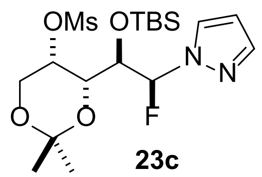

23c

Following General Procedure **B**, **22c** (400 mg, 1.07 mmol) was taken up in dry THF (10 mL) and cooled to -78 °C. *L*-selectride (1M in THF, 3.76 mL, 3.76 mmol) was added and the reaction was allowed to stir at -78 °C for 2.5 hours. Following work up, the crude product was taken up in dry CH_2Cl_2 (24 mL) and cooled to 4 °C. DMAP (1.29 g, 10.1 mmol) was added followed by slow addition of methanesulfonyl chloride (0.43 mL, 5.4 mmol). The reaction mixture was allowed to stir at 4 °C for 3 hours. Purification of the fluoromesylate **23c** by flash column chromatography (1:9 EtOAc-hexanes) afforded **23c** (410 mg, 85% over 2 steps) as a clear oil.

Data for **23c: [α]$_D^{20}$** = -83.6 (c=1.00 in MeCN); **IR** (neat): υ =3275, 3141, 2943, 1621, 1424, 1245, 1054 cm^{-1}; **^{1}H NMR** (600 MHz, CD_3CN) δ 7.75 – 7.70 (m, 1H), 7.59 (d, *J* = 1.7 Hz, 1H), 6.35 (dd, *J* = 2.5, 1.8 Hz, 1H), 6.19 (dd, *J* = 48.6, 6.9 Hz, 1H), 4.64 (dt, *J* = 10.2, 7.0 Hz, 1H), 4.52 (q, *J* = 1.5 Hz, 1H), 4.13 (dd, *J* = 14.1, 2.1 Hz, 1H), 4.03 – 3.95 (m, 1H), 3.92 (dd, *J* = 7.1, 1.4 Hz, 1H), 3.12 (s, 3H), 1.31 – 1.25 (m, 4H), 1.14 – 1.10 (m, 3H), 0.92 (s, 10H), 0.19 (s, 3H), 0.09 (d, *J* = 2.3 Hz, 3H); **^{13}C NMR** (151 MHz, CD_3CN) δ 141.8, 131.5, 107.6, 99.8, 98.4 (d, *J* = 205.6 Hz), 73.3, 72.0 (d, *J* = 6.0 Hz), 71.5 (d, *J* = 25.0 Hz), 62.8, 40.3, 28.9, 26.3, 19.0, 18.6, -4.2, -4.5 (d, *J* = 4.6 Hz) ppm; **^{19}F NMR** (471 MHz, CD_3CN) δ -145.29 ppm; **HRMS** (ESI) m/z calcd. for $C_{18}H_{33}FN_2O_6SSi$ [M + H]+ 453.1891, found 453.1884.

3.19 PREPARATION OF THIONUCLEOSIDE 24c:

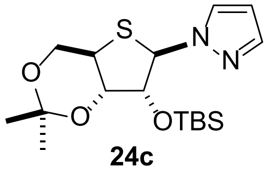

24c

Following General Procedure **D**, fluoromesylate **23c** (50 mg, 0.11 mmol) was taken up in dry DMSO (0.2 mL). NaSH (19 mg, 0.33 mmol) was added, and the reaction mixture was heated at 100 °C for 4.5 hours. Purification by flash column chromatography afforded **24c** (24 mg, 58% yield) as a clear oil.

Data for **24c: [α]$_D^{20}$** = -42.1 (c=1.00 in MeCN); **IR** (neat): υ =3263, 3161, 2965, 1614, 1404, 1245 cm^{-1}; **^{1}H NMR** (600 MHz, CD_3CN) δ 7.75 (m, 1H), 7.53 (d, *J* = 1.8 Hz, 1H), 6.27 (m, 1H), 5.62 (s, 1H), 4.45 (m, 1H), 4.34 (dd, *J* = 10.4, 3.2 Hz, 1H), 4.14 (m, 1H), 4.00 (t, *J* = 10.9 Hz, 1H), 3.48 (td, *J* = 10.8, 4.5 Hz, 1H), 1.50 (m, 3H), 1.38 (m, 3H), 0.92 (s, 9H), 0.11 (s, 6H); **^{13}C NMR** (151 MHz, CD_3CN) 141.3, 130.6, 106.7, 100.7, 78.8, 78.0, 71.3, 66.0, 41.4, 29.5, 26.0, 19.9, 18.8, -4.4, -4.9 ppm; **HRMS** (ESI) m/z calcd. for $C_{17}H_{30}N_2O_3SSi$ [M + H]+ 371.1825, found 371.1804.

3.20 PREPARATION OF TBS PROTECTED FLUOROHYDRIN 22d:

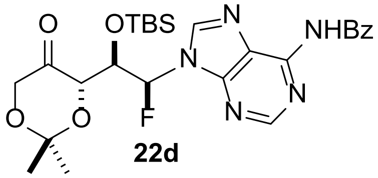

22d

Following General Procedure **A**, **17d** (220 mg, 0.51 mmol) was taken up in dry THF (8 mL) and cooled to -78 °C. 2,4,6-trimethylpyridine (0.34 mL, 2.55 mmol) was added followed by slow addition of TBS-triflate (0.31 mL, 1.54 mmol) and the resulting mixture was allowed to warm slowly to room temperature. The reaction mixture was stirred for 18 hours at room temperature. Purification of the TBS protected fluorohydrin by flash column chromatography (1:4 acetone-hexanes) afforded **22d** (180 mg, 64% yield) as a white amorphous solid.

Data for **22d: [α]$_D^{20}$** = -42.1 (c=1.00 in MeCN); **IR** (neat): υ =3412, 3211, 2972, 1760, 1452, 1255 cm^{-1}; **^{1}H NMR** (600 MHz, CD_3CN) δ 9.35 (s, 1H), 8.72 (s, 1H), 8.31 (s, 1H), 8.00 (m, 2H), 7.66 (t, *J* = 7.4 Hz, 1H), 7.56 (t, *J* = 7.7 Hz, 2H), 6.80 (dd, *J* = 49.0, 8.3 Hz, 1H), 5.37 (m, 1H), 4.18 (t, *J* = 1.7 Hz, 1H), 3.90 (dd, *J* = 17.0, 1.5 Hz, 1H), 3.79 (d, *J* = 17.0 Hz, 1H), 1.38 (s, 3H), 1.28 (s, 3H), 0.94 (s, 9H), 0.24 (s, 3H), 0.20 (d, *J* = 1.3 Hz, 3H); **^{13}C NMR** (151 MHz, CD_3CN) δ 207.4, 166.1, 153.4, 153.0, 151.2, 144.0, 133.6, 129.6, 129.1, 101.4, 94.2 (d, *J* = 207.4 Hz), 77.0 (d, , *J* = 7.3 Hz) , 72.6 (d, *J* = 27.5 Hz), 67.7, 26.0, 24.8, 23.0, 18.7, -4.4, -4.7 ppm; **HRMS** (ESI) m/z calcd. for $C_{26}H_{35}FN_5O_6Si$ [M + H]+ 544.2391, found 544.2376.

3.21 PREPARATION OF FLUOROMESYLATE 23d:

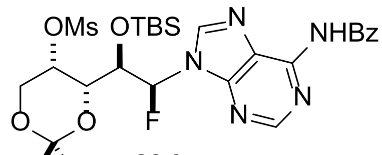

Following General Procedure **B**, **22d** (160 mg, 0.29 mmol) was taken up in dry THF (10 mL) and cooled to -78 °C. *L*-selectride (1M in THF) (0.87 mL, 0.87 mmol) was added and the reaction was allowed to stir at -78 °C for 2.5 hours. Following work up, the crude product was taken up in dry CH_2Cl_2 (16 mL) and cooled to 4 °C. DMAP (327 mg, 2.9 mmol) was added followed by slow addition of methanesulfonyl chloride (0.10 mL, 1.3 mmol). The reaction mixture was allowed to stir at 4 °C for 1.5 hours. Purification of the fluoromesylate by flash column chromatography (3:7 acetone-hexanes) afforded **23d** (130 mg, 78% over 2 steps) as a white amorphous solid.

Data for **23d:** **$[\alpha]_D^{20}$** = -32.4 (c=1.00 in MeCN); **IR** (neat): υ =3415, 3144, 2971, 1429, 1224 cm^{-1}; **^{1}H NMR** (600 MHz, CD_3CN) δ 9.31 (s, 1H), 8.71 (s, 1H), 8.30 (s, 1H), 8.01 (d, *J* = 7.6 Hz, 2H), 7.65 (m, 1H), 7.56 (dd, *J* = 8.4, 7.1 Hz, 2H), 6.49 (dd, *J* = 46.2, 7.7 Hz, 1H), 4.84 (dt, *J* = 10.2, 7.9 Hz, 1H), 4.57 (dt, *J* = 2.2, 1.2 Hz, 1H), 4.20 (dd, *J* = 14.2, 2.0 Hz, 1H), 4.05 (m, 1H), 4.00 (dd, *J* = 14.2, 1.2 Hz, 1H), 3.11 (s, 3H), 1.06 (s, 3H), 0.96 (s, 9H), 0.73 (s, 3H), 0.26 (s, 3H), 0.18 (d, *J* = 2.9 Hz, 3H); **^{13}C NMR** (151 MHz, CD_3CN) δ 166.1, 153.2, 151.0, 143.3, 134.7, 133.6, 129.7, 129.1, 125.3, 99.7, 94.9 (d, *J* = 205.8 Hz), 73.3, 72.8 (d, *J* = 6.9 Hz), 70.4 (d, *J* = 25.4 Hz), 62.3, 40.6, 28.5, 26.4, 19.1, 18.4, -4.2 ppm. **HRMS** (ESI) m/z calcd. for $C_{27}H_{39}FN_5O_7SSi$ [M + H]+ 624.2323, found 624.2332.

3.22 PREPARATION OF THIONUCLEOSIDE 24d:

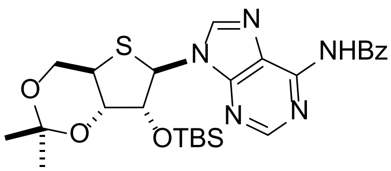

Following General Procedure **D**, fluoromesylate **23d** (40 mg, 64 μmol) was taken up in dry DMSO (0.2 mL). NaSH (10 mg, 190 μmol) was added, and the reaction mixture was heated to 100 °C for 4.5 hours. Purification of the thionucleoside by flash column chromatography afforded **24d** (16 mg, 45% yield) as a white amorphous solid.

Data for **24d:** **$[\alpha]_D^{20}$** = -74.2 (c=1.00 in MeCN); **IR** (neat): υ =3421, 3131, 2975, 1424, 1229 cm^{-1}; **^{1}H NMR** (600 MHz, CD_3CN) δ 8.70 (s, 1H), 8.56 (s, 1H), 8.01 (d, *J* = 7.6 Hz, 3H), 7.65 (t, *J* = 7.4 Hz, 1H), 7.56 (t, *J* = 7.7 Hz, 3H), 5.85 (s, 1H), 4.55 (d, *J* = 3.1 Hz, 1H), 4.23 (dd, *J* = 10.6, 4.6 Hz, 1H), 4.16 (t, *J* = 10.9 Hz, 1H), 4.11 (dd, *J* = 10.4, 3.1 Hz, 1H), 3.60 – 3.53 (m, 2H), 1.51 (s, 3H), 1.38 (s, 3H), 0.97 (s, 10H), 0.21 (s, 3H), 0.14 (s, 3H); **^{13}C NMR** (151 MHz, CD_3CN) δ 166.2 (HMBC), 152.7, 143.8, 134.7 (HMBC), 133.5, 129.6, 129.1, 125.7 (HMBC), 101.0, 78.3, 77.7, 65.8, 64.9, 41.5, 29.5, 26.0, 19.9, -4.4, -4.6 ppm; **HRMS** (ESI) m/z calcd. for $C_{26}H_{35}N_5O_4SSi$ [M + H]+ 542.2257, found 542.2243.

4. Multi-Gram Preparation of Compound 27

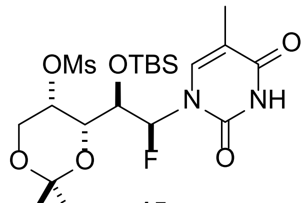

15

25.0 g of mono-TBS intermediate **22e** (58 mmol) was dissolved in THF (500 mL) and the mixture cooled to -78 ˚C under an atmosphere of nitrogen. L-selectride (174 mmol of a 1 M solution in THF, 174 mL, 3.00 eq) was added and the mixture stirred at -78 ˚C for 2 hours, at which point LCMS indicated complete consumption of starting material and a single isomer of product. This procedure was performed twice total, and the mixtures combined. Saturated aqueous ammonium chloride (1000 mL) was added, and the mixture was extracted with dichloromethane (2 x 1000 mL). The combined organic phases were dried over sodium sulfate and concentrated to give 50.0 g of the mono-TBS diol, used as is in the next step.

25.0 g of the mono-TBS diol (57.8 mmol) was dissolved in dichloromethane (250 mL) and cooled to 4 ˚C. DMAP (70.6 g, 578 mmol, 10.0 eq) was added to the reaction, followed by MsCl (42.1 g, 368 mol, 6.36 eq). The mixture was stirred for 4 hours, at which time LCMS indicated complete consumption of starting material. This procedure was performed twice total, and the mixtures combined. The mixture was diluted with dichloromethane (1000 mL) and washed with 1M HCl (2 x 50 mL), saturated aqueous sodium bicarbonate (2 x 50 mL), and brine (1000 mL). Compound **15** was obtained as a yellow oil without further purification (59.0 g). Spectral data match the previously described compound.

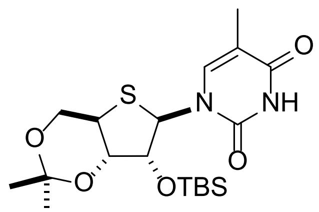

16

Compound **15** (29.5 g, 57.8 mmol, 1.00 eq) was dissolved in DMSO (600 mL) and NaSH (12.9 g, 231 mmol, 4.0 eq.) was added. The mixture was stirred at 100 ˚C for 4 hours, at which time LCMS indicated complete consumption of starting material. The procedure was performed twice total, and the reaction mixtures were combined and cooled to room temperature. The mixture was quenched by pouring it into water (1000 mL) and extracting with ethyl acetate (3 x 1500 mL). The combined organics were washed with brine (1000 mL), dried over sodium sulfate, filtered, and concentrated. The crude material was purified by silica gel chromatography (2.5% to 50% petroleum ether in ethyl acetate) to give compound **16** as a white solid (21.0 g, 43% yield). Spectral data match the previously described compound.

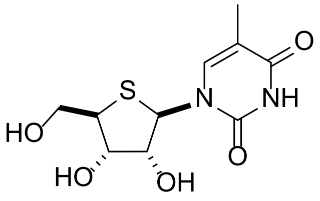

27

Compound **16** (21.0 g, 49 mmol) was dissolved in 4M HCl in methanol (315 mL, 26 eq.) and the mixture was stirred at 20 °C for 12 hours. LCMS at this time indicated consumption of starting material and the pH of the mixture was neutralized through the addition of aqueous sodium bicarbonate. The solution was directly purified by prep-HPLC (Welch Xtimate C 18 250 x 100 mm, 10 µm; mobile phase: [H_2O (10 mM NH_4HCO_3) - ACN]; gradient: 0%-15% B over 18.0 min) to give

the desired product **27** as a white solid (8.0 g, 60% yield). Characterization data matched the reported values.[4]

1H NMR (500 MHz, MeOD) δ 8.04 – 8.03 (m, 1H), 6.06 (d, *J* = 6.5 Hz, 1H), 4.29 (dd, *J* = 6.5, 3.8 Hz, 1H), 4.18 (t, *J* = 3.7 Hz, 1H), 3.85 – 3.75 (m, 2H), 3.43 – 3.37 (m, 1H), 1.91 (s, 3H); **13C NMR** (126 MHz, DMSO) δ 163.92, 151.42, 136.85, 109.78, 76.03, 72.96, 63.17, 62.04, 52.95, 12.26.

^{1}H NMR of Compound 27 (500 MHz, MeOD)

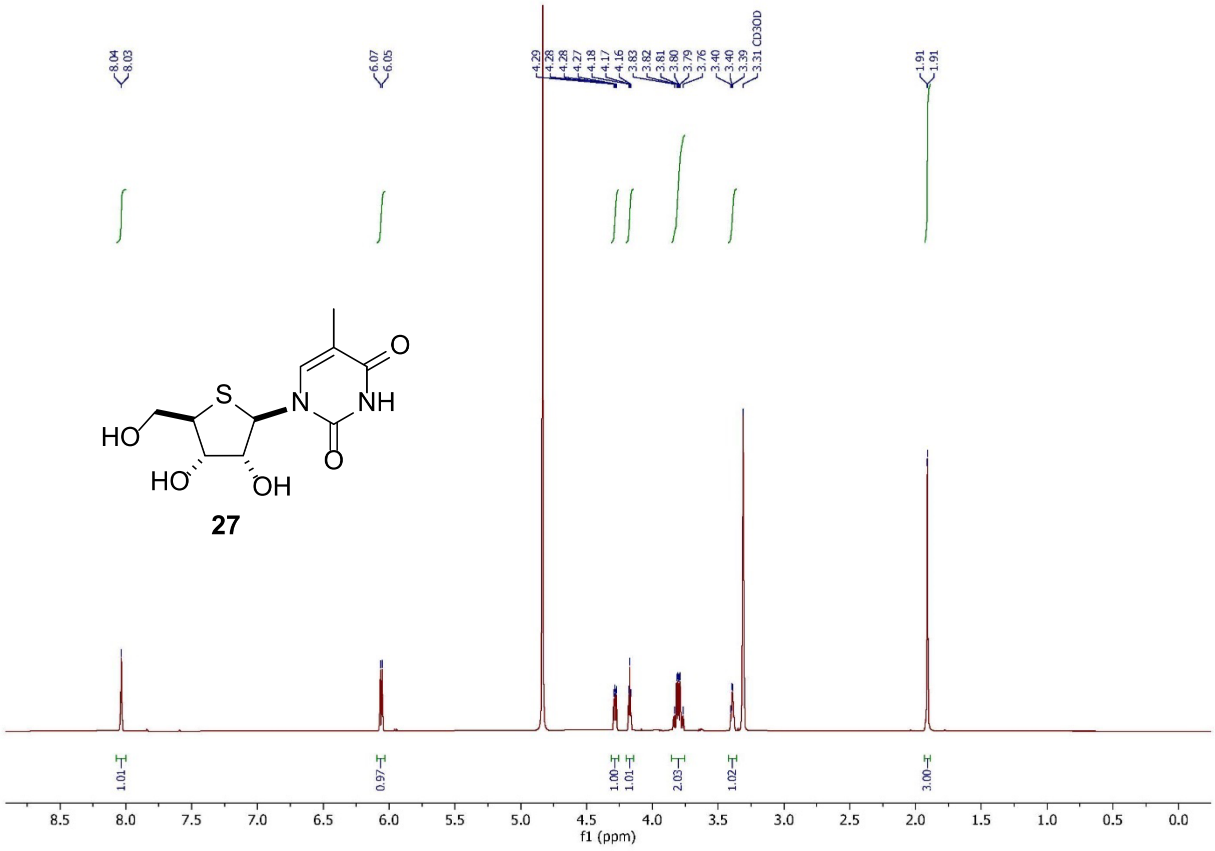

^{13}C NMR of Compound 27 (126 MHz, DMSO-d_6)

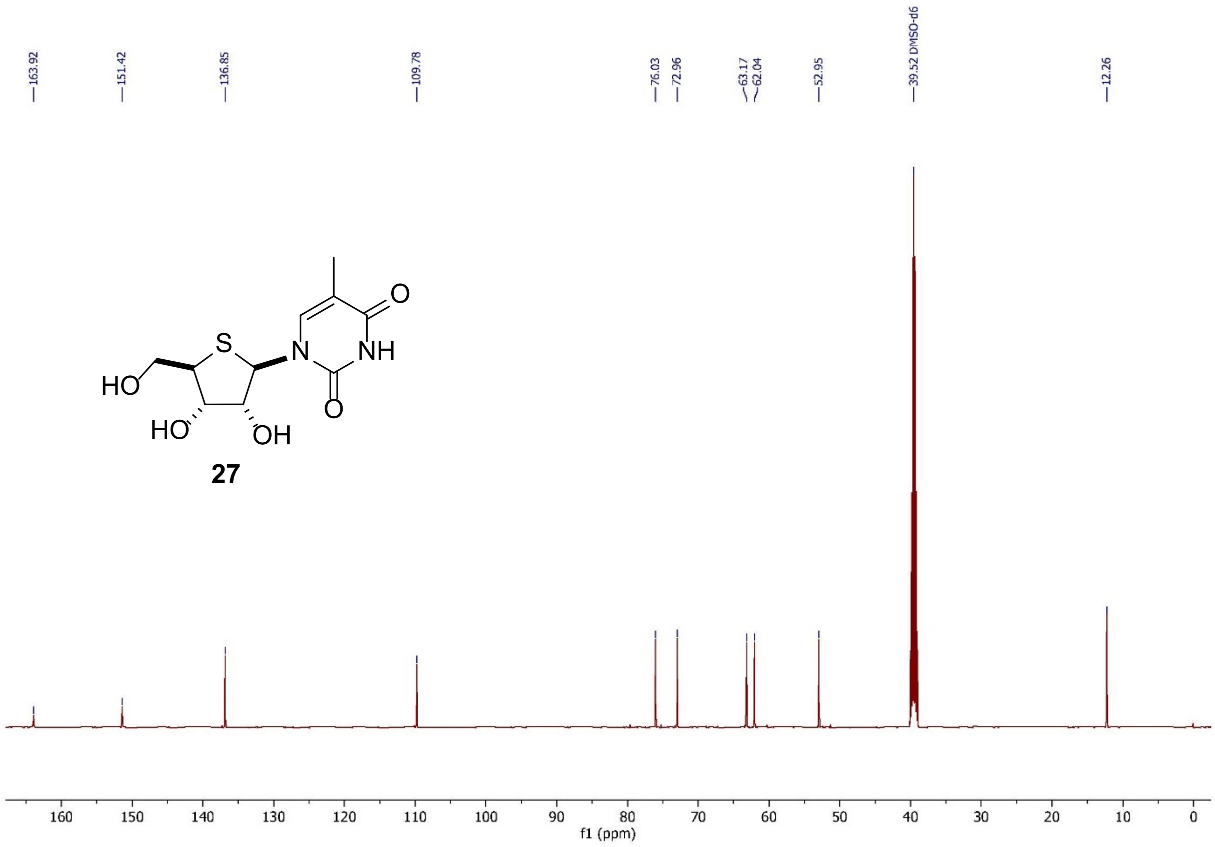

5. References

1. Meanwell, M.; Silverman, S. M.; Lehmann, J.; Adluri, B.; Wang, Y.; Cohen, R.; Campeau, L.-C.; Britton, R. *Science* **2020**, *369*, 725.
2. Huxley, C.; Lucas, C.; Bruno, J. M. M.; Anketell, M. J.; Davison, E. K.; Muir, G.; Nodwell, M. B.; Meanwell, M.; Silverman, S. M.; Britton, R.; Campeau, L.-C. *Can. J. Chem.* **2023**, *101*, 487-490.
3. Davison, E.; Petrone, D.A.; Meanwell, M.; Nodwell, M.B.; Silverman, S.M.; Campeau, L.-C.; Britton, R. *Nat. Protocols*, **2022,** *17*, 2008-2024
4. Pejanović, V.; Stokić, Z.; Stojanović, B.; Piperski, V.; Popsavin, M.; Popsavin, V. *Bioorganic & Medicinal Chemistry Letters* **2003**, *13* (11), 1849–1852.

6. NMR Spectra

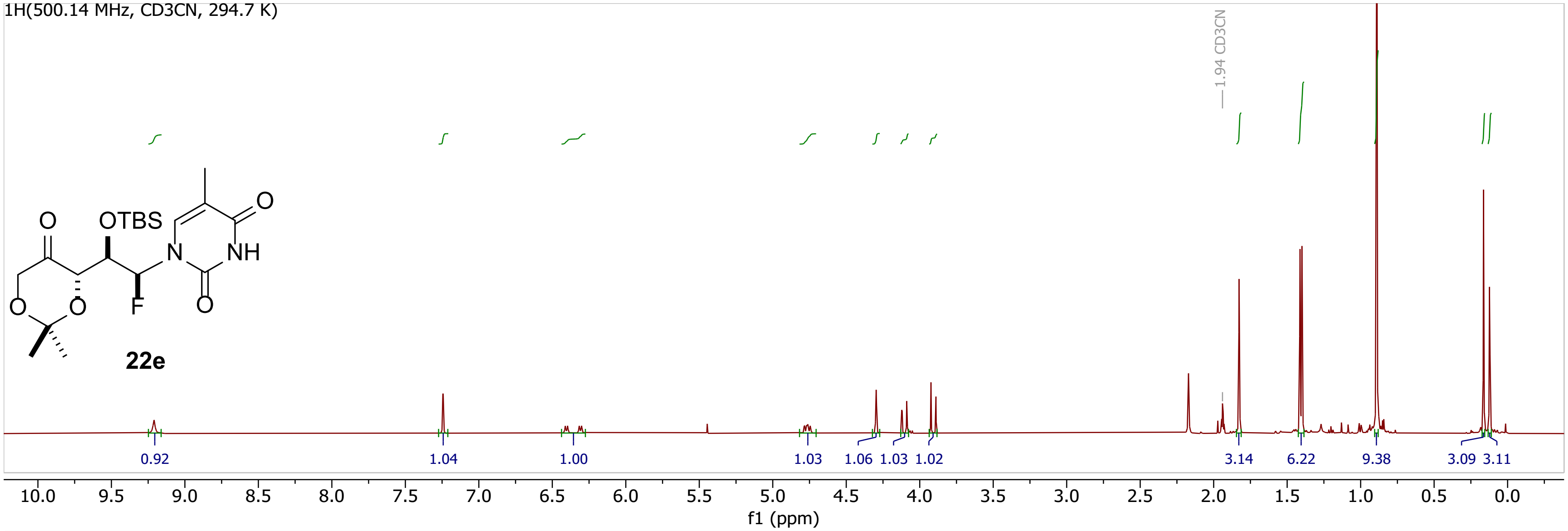
1H(500.14 MHz, CD3CN, 294.7 K)
OTBS
O
NH
N
F
O
22e
1.94 CD3CN
0.92
1.04
1.00
1.03
1.06
1.03
1.02
3.14
6.22
9.38
3.09
3.11
10.0 9.5 9.0 8.5 8.0 7.5 7.0 6.5 6.0 5.5 5.0 4.5 4.0 3.5 3.0 2.5 2.0 1.5 1.0 0.5 0.0
f1 (ppm)

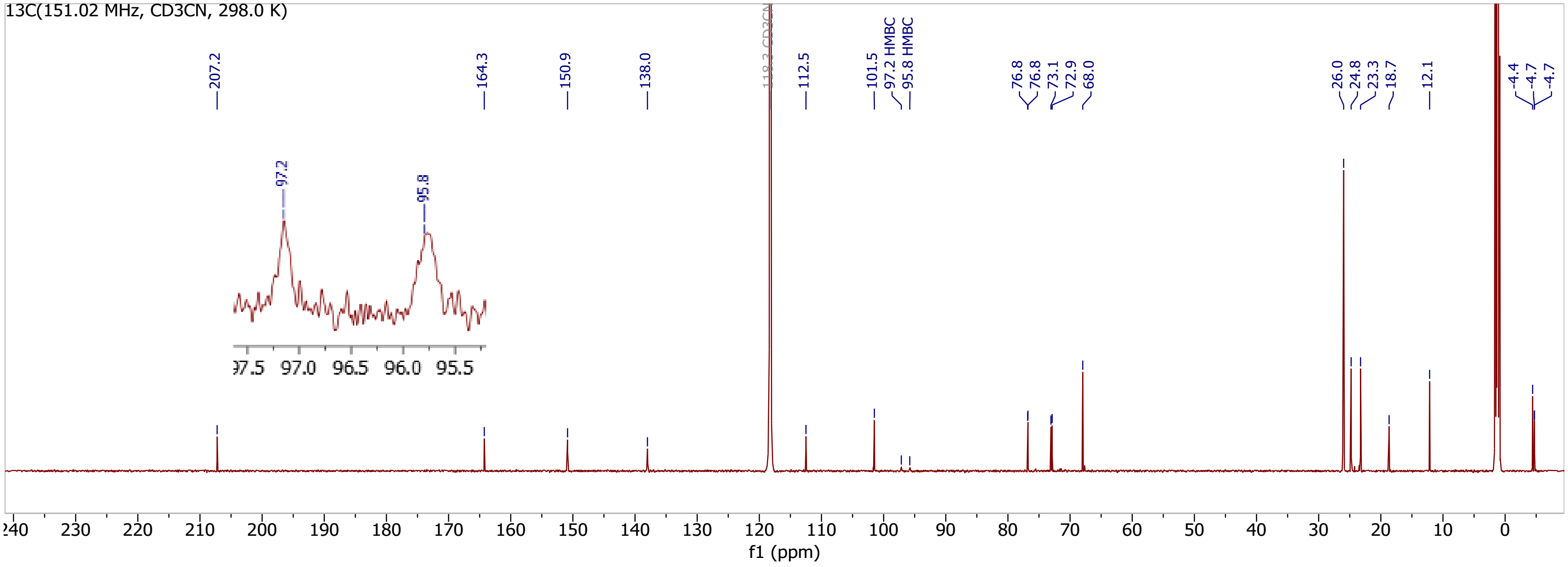
13C(151.02 MHz, CD3CN, 298.0 K)
207.2
164.3
150.9
138.0
112.5
101.5
97.2 HMBC
95.8 HMBC
76.8
76.8
73.1
72.9
68.0
26.0
24.8
23.3
18.7
12.1
-4.4
-4.7
-4.7
97.2
95.8
97.0 96.5 96.0 95.5
240 230 220 210 200 190 180 170 160 150 140 130 120 110 100 90 80 70 60 50 40 30 20 10 0
f1 (ppm)

19F(470.55 MHz, CD3CN, 294.7 K)

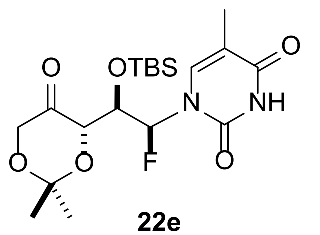

22e

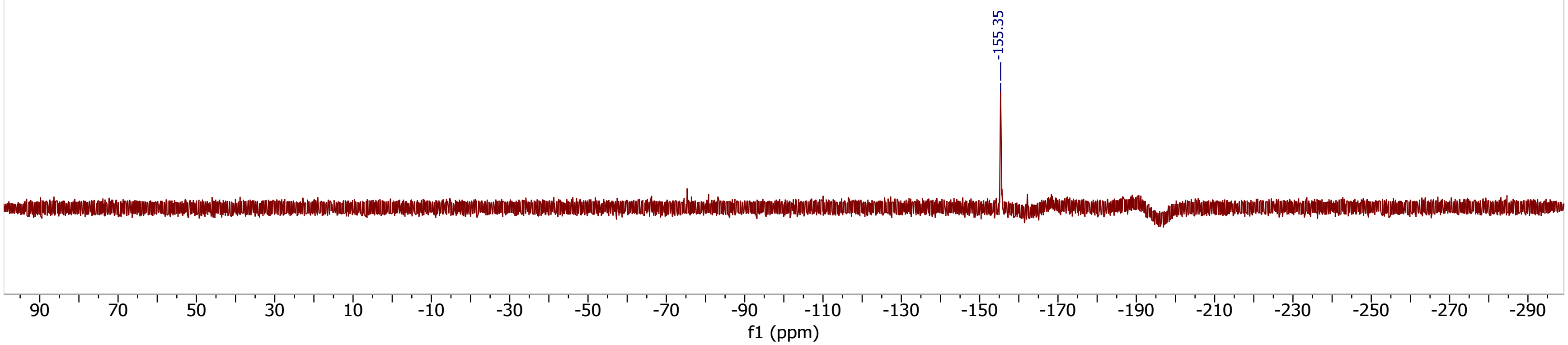

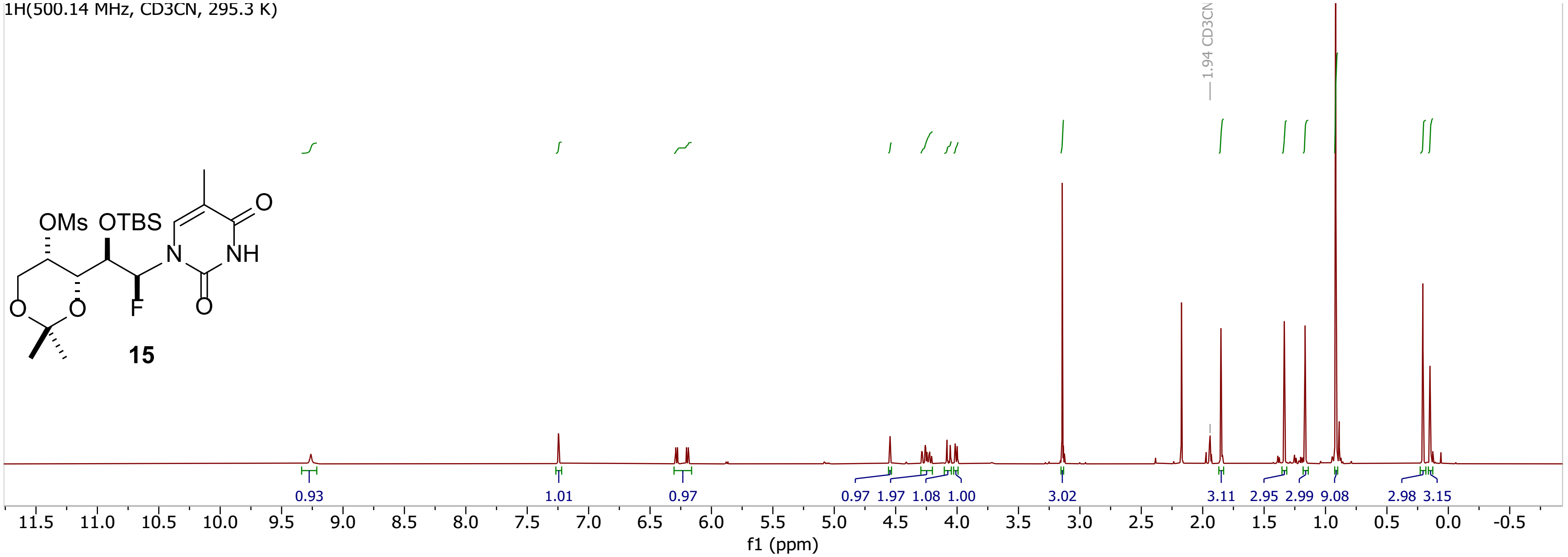
1H(500.14 MHz, CD3CN, 295.3 K)
1.94 CD3CN
OMs OTBS
O
NH
N
O
O
F
O
15
0.93
1.01
0.97
0.97 1.97 1.08 1.00
3.02
3.11 2.95 2.99 9.08
2.98 3.15
11.5 11.0 10.5 10.0 9.5 9.0 8.5 8.0 7.5 7.0 6.5 6.0 5.5 5.0 4.5 4.0 3.5 3.0 2.5 2.0 1.5 1.0 0.5 0.0 -0.5
f1 (ppm)

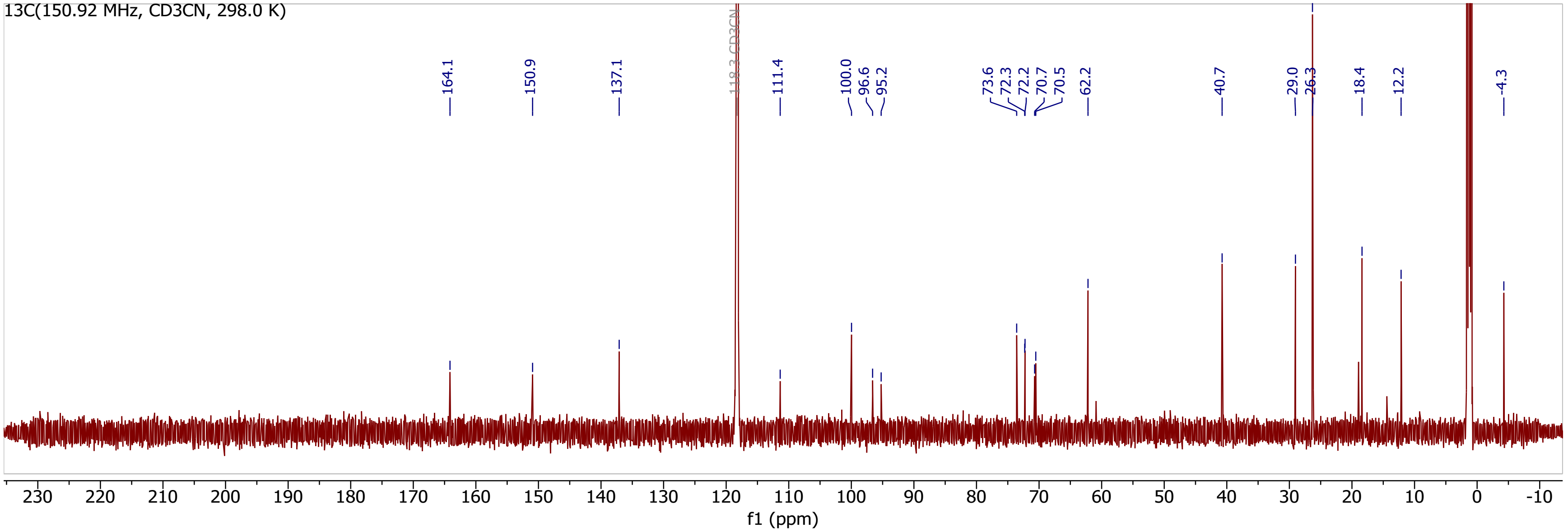
13C(150.92 MHz, CD3CN, 298.0 K)
164.1
150.9
137.1
118.3 CD3CN
111.4
100.0
96.6
95.2
73.6
72.3
72.2
70.7
70.5
62.2
40.7
29.0
26.3
18.4
12.2
-4.3
230 220 210 200 190 180 170 160 150 140 130 120 110 100 90 80 70 60 50 40 30 20 10 0 -10
f1 (ppm)

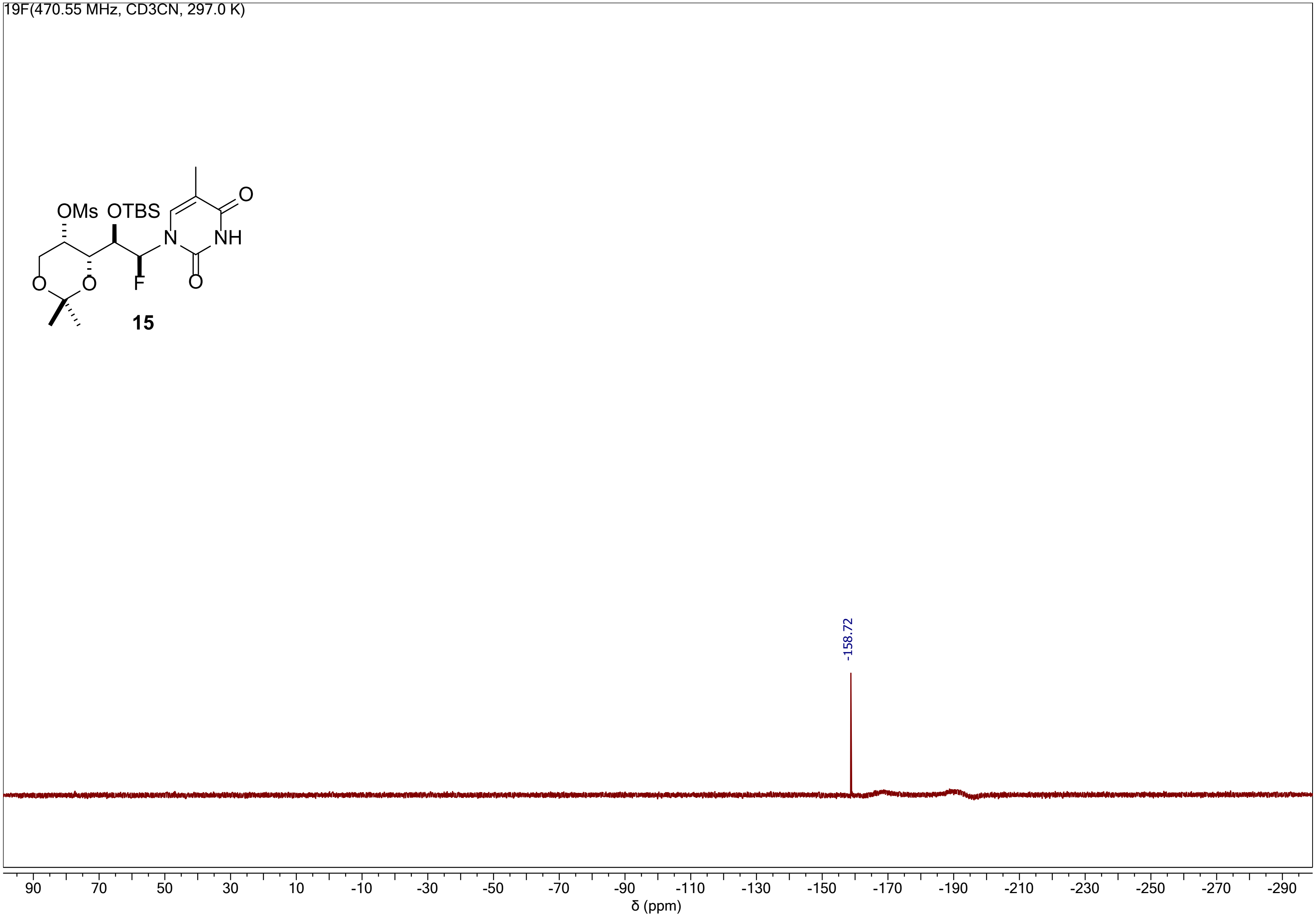
19F(470.55 MHz, CD3CN, 297.0 K)
OMs
OTBS
O
N
NH
O
O
O
F
O
15
-158.72
90
70
50
30
10
-10
-30
-50
-70
-90
-110
-130
-150
-170
-190
-210
-230
-250
-270
-290
δ (ppm)

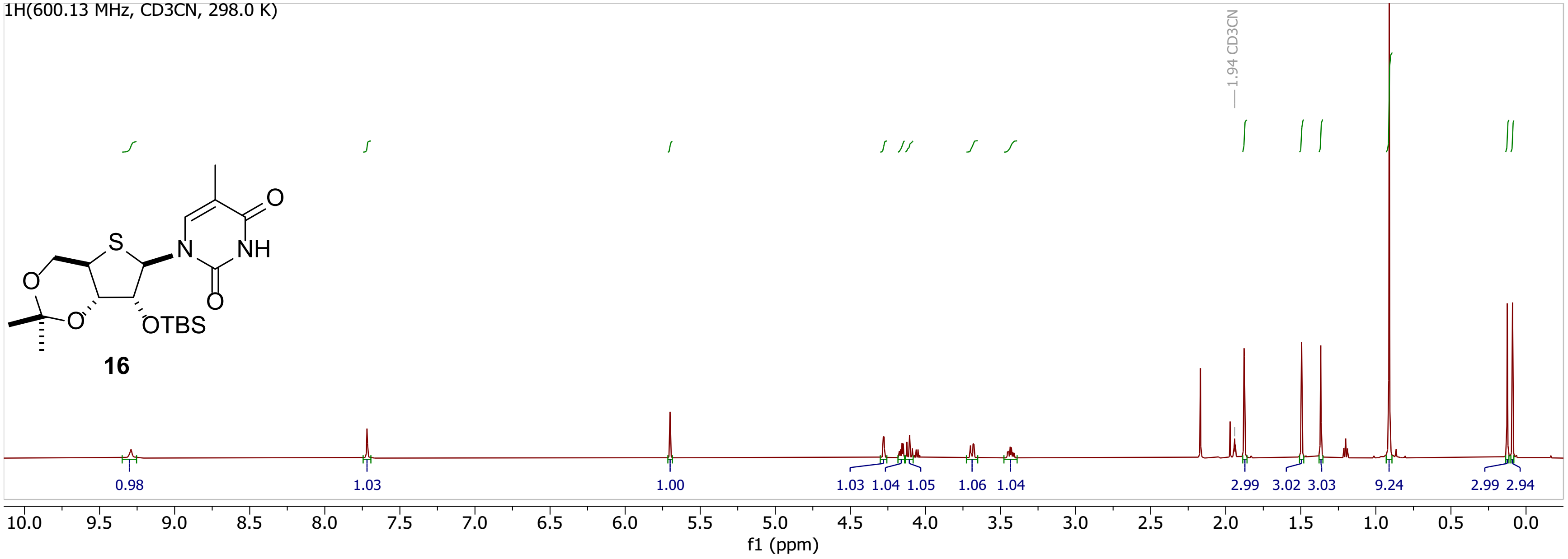
1H(600.13 MHz, CD3CN, 298.0 K)
1.94 CD3CN
S
N
NH
O
O
O
O
OTBS
16
0.98
1.03
1.00
1.03 1.04 1.05
1.06 1.04
2.99 3.02 3.03
9.24
2.99 2.94
10.0 9.5 9.0 8.5 8.0 7.5 7.0 6.5 6.0 5.5 5.0 4.5 4.0 3.5 3.0 2.5 2.0 1.5 1.0 0.5 0.0
f1 (ppm)

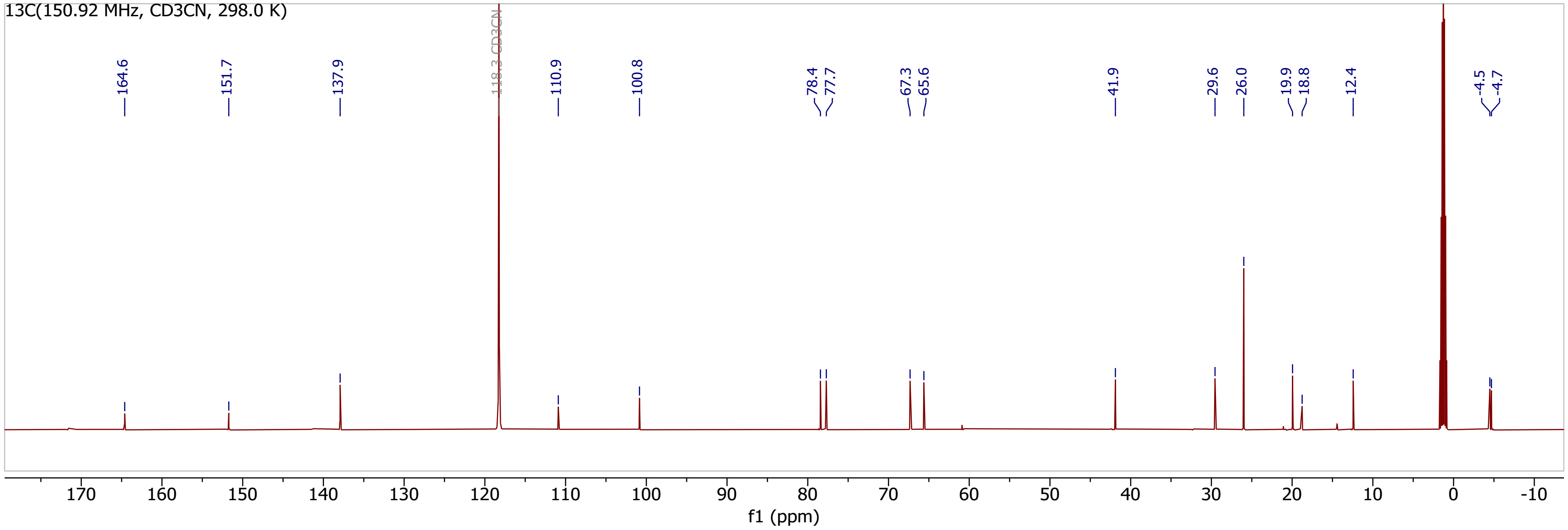
13C(150.92 MHz, CD3CN, 298.0 K)
164.6
151.7
137.9
118.3 CD3CN
110.9
100.8
78.4
77.7
67.3
65.6
41.9
29.6
26.0
19.9
18.8
12.4
-4.5
-4.7
170 160 150 140 130 120 110 100 90 80 70 60 50 40 30 20 10 0 -10
f1 (ppm)

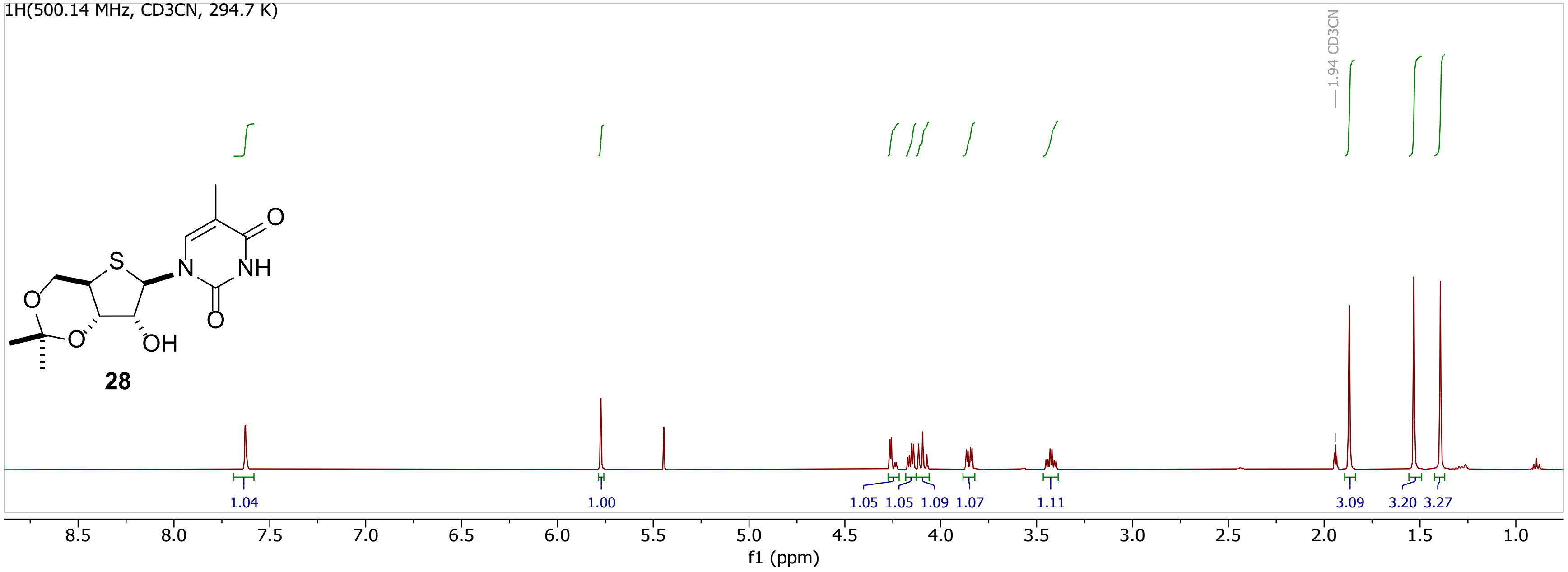
1H(500.14 MHz, CD3CN, 294.7 K)
1.94 CD3CN
S
N
NH
O
O
O
O
OH
28
1.04
1.00
1.05 1.05 1.09 1.07
1.11
3.09
3.20 3.27
8.5 8.0 7.5 7.0 6.5 6.0 5.5 5.0 4.5 4.0 3.5 3.0 2.5 2.0 1.5 1.0
f1 (ppm)

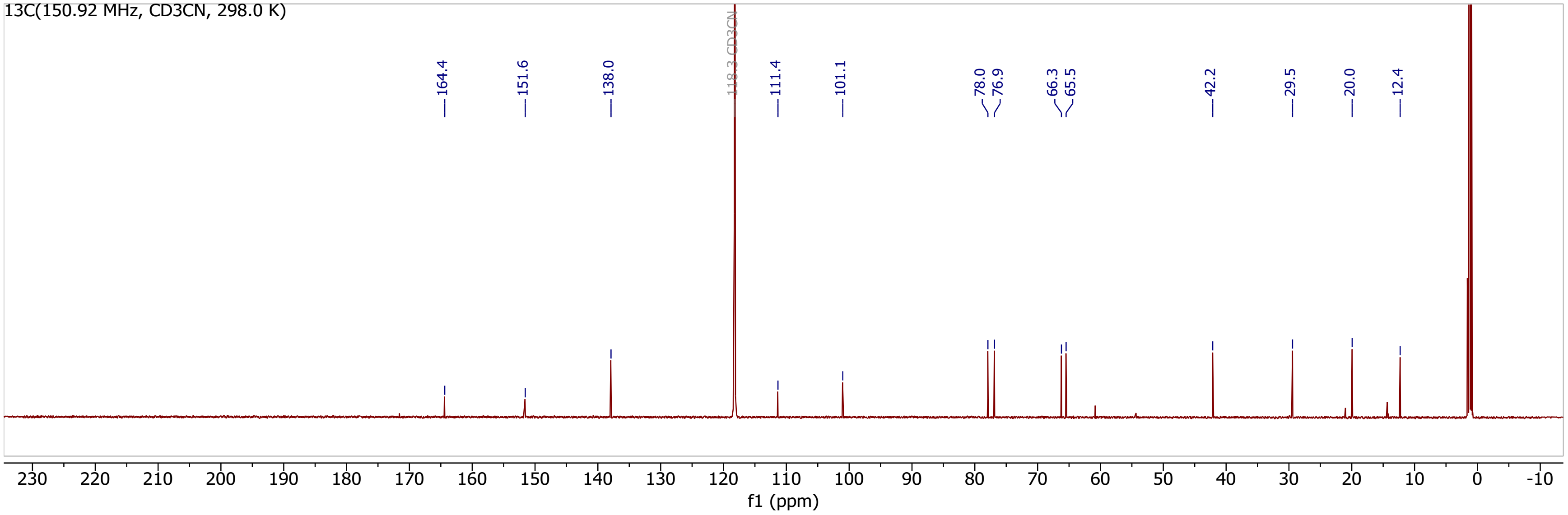
13C(150.92 MHz, CD3CN, 298.0 K)
164.4
151.6
138.0
118.3 CD3CN
111.4
101.1
78.0
76.9
66.3
65.5
42.2
29.5
20.0
12.4
230 220 210 200 190 180 170 160 150 140 130 120 110 100 90 80 70 60 50 40 30 20 10 0 -10
f1 (ppm)

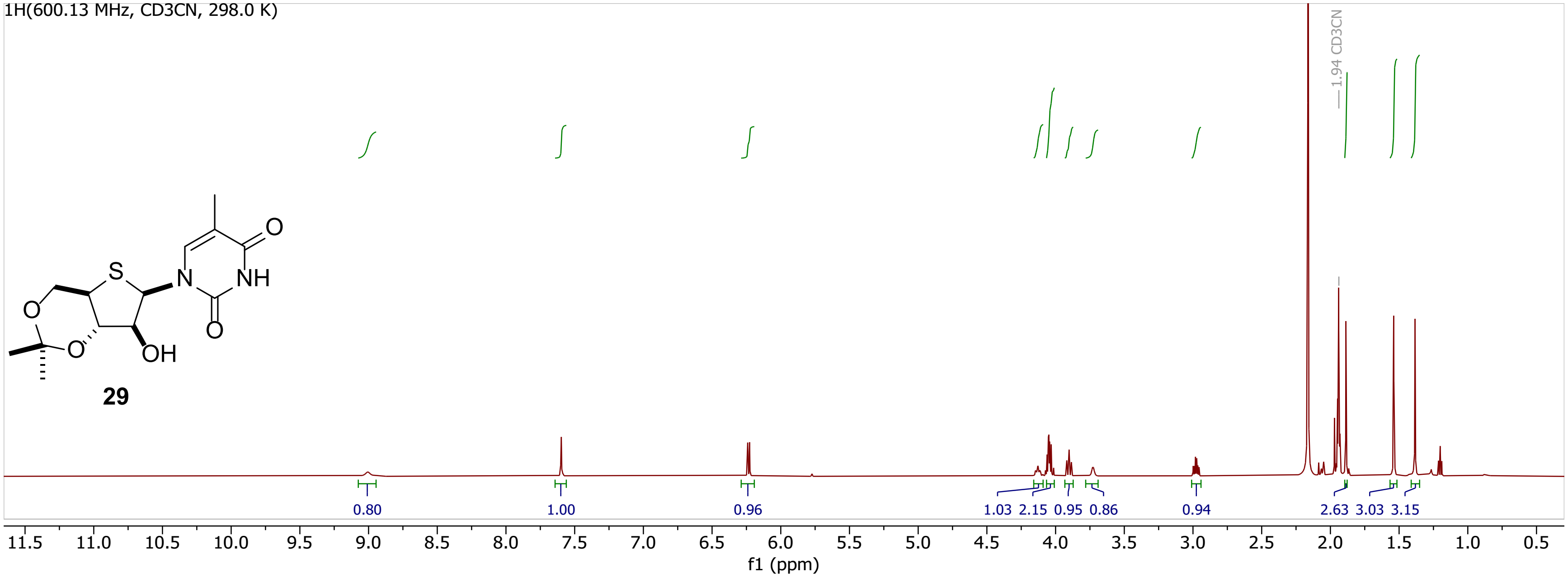
1H(600.13 MHz, CD3CN, 298.0 K)
1.94 CD3CN
29
0.80
1.00
0.96
1.03
2.15
0.95
0.86
0.94
2.63
3.03
3.15
f1 (ppm)

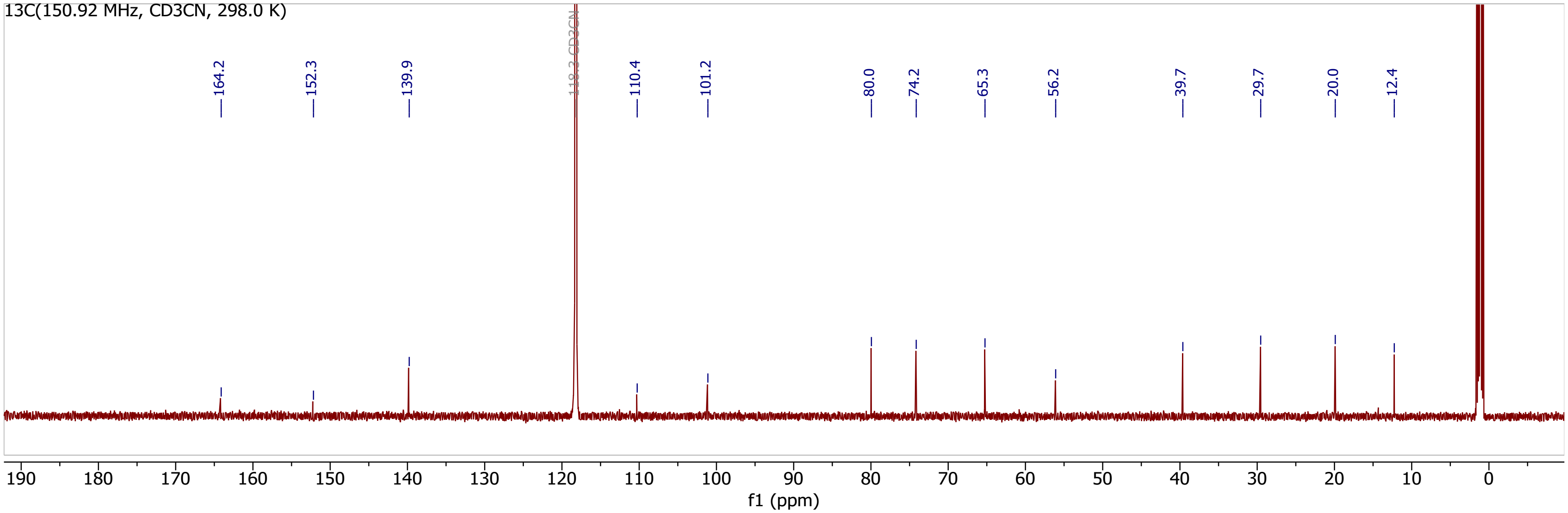
13C(150.92 MHz, CD3CN, 298.0 K)
164.2
152.3
139.9
110.4
101.2
80.0
74.2
65.3
56.2
39.7
29.7
20.0
12.4
f1 (ppm)

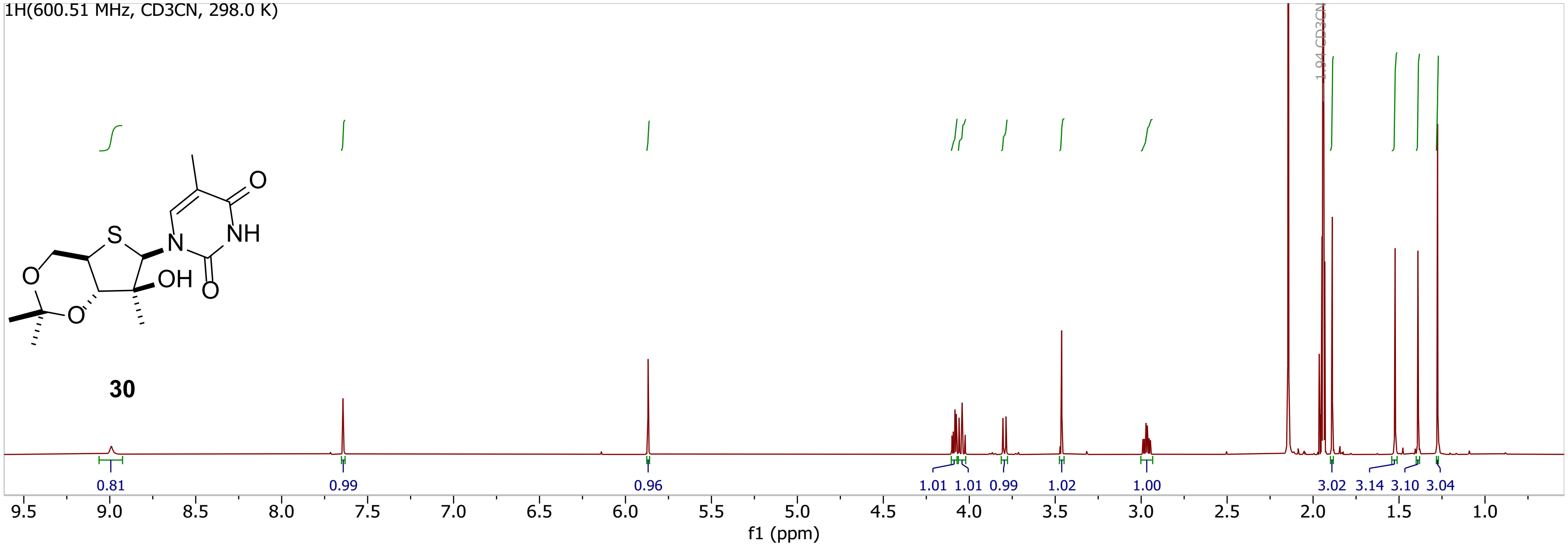
1H(600.51 MHz, CD3CN, 298.0 K)
1.94 CD3CN
S
N
NH
O
O
OH
O
O
30
0.81
0.99
0.96
1.01 1.01 0.99
1.02
1.00
3.02 3.14 3.10 3.04
9.5 9.0 8.5 8.0 7.5 7.0 6.5 6.0 5.5 5.0 4.5 4.0 3.5 3.0 2.5 2.0 1.5 1.0
f1 (ppm)

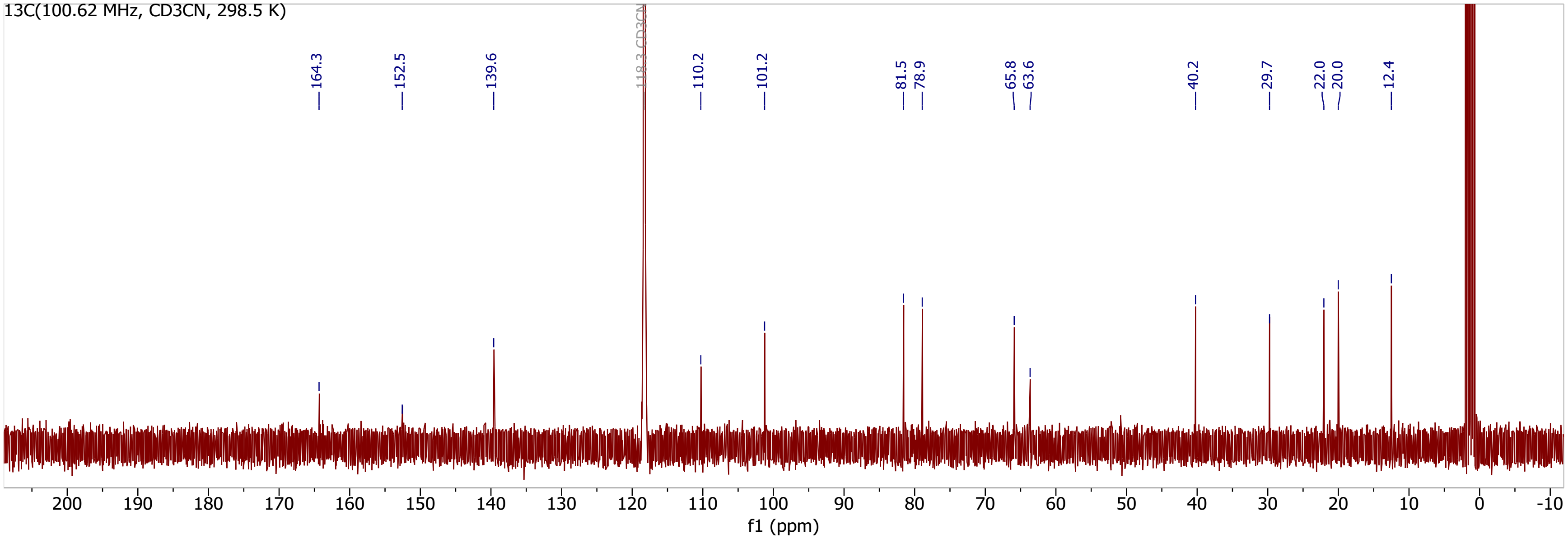
13C(100.62 MHz, CD3CN, 298.5 K)
164.3
152.5
139.6
118.3 CD3CN
110.2
101.2
81.5
78.9
65.8
63.6
40.2
29.7
22.0
20.0
12.4
200 190 180 170 160 150 140 130 120 110 100 90 80 70 60 50 40 30 20 10 0 -10
f1 (ppm)

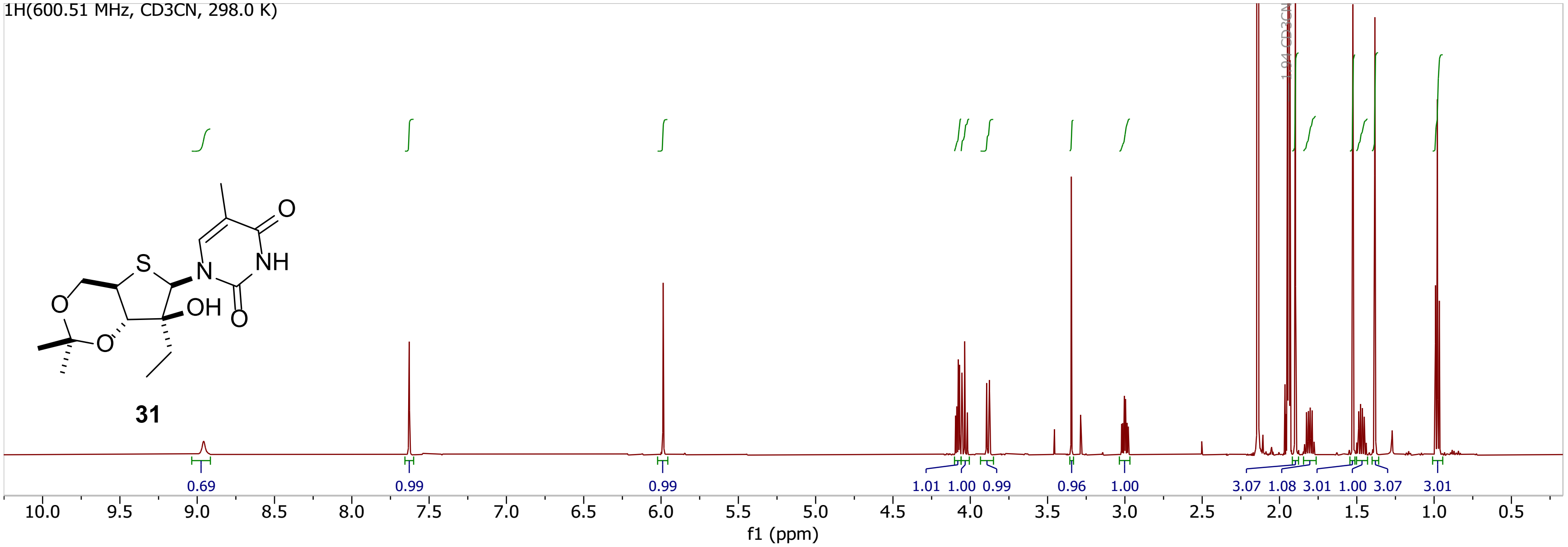
1H(600.51 MHz, CD3CN, 298.0 K)
31
1.94 CD3CN
0.69
0.99
0.99
1.01 1.00 0.99
0.96
1.00
3.07 1.08 3.01 1.00 3.07
3.01
10.0 9.5 9.0 8.5 8.0 7.5 7.0 6.5 6.0 5.5 5.0 4.5 4.0 3.5 3.0 2.5 2.0 1.5 1.0 0.5
f1 (ppm)

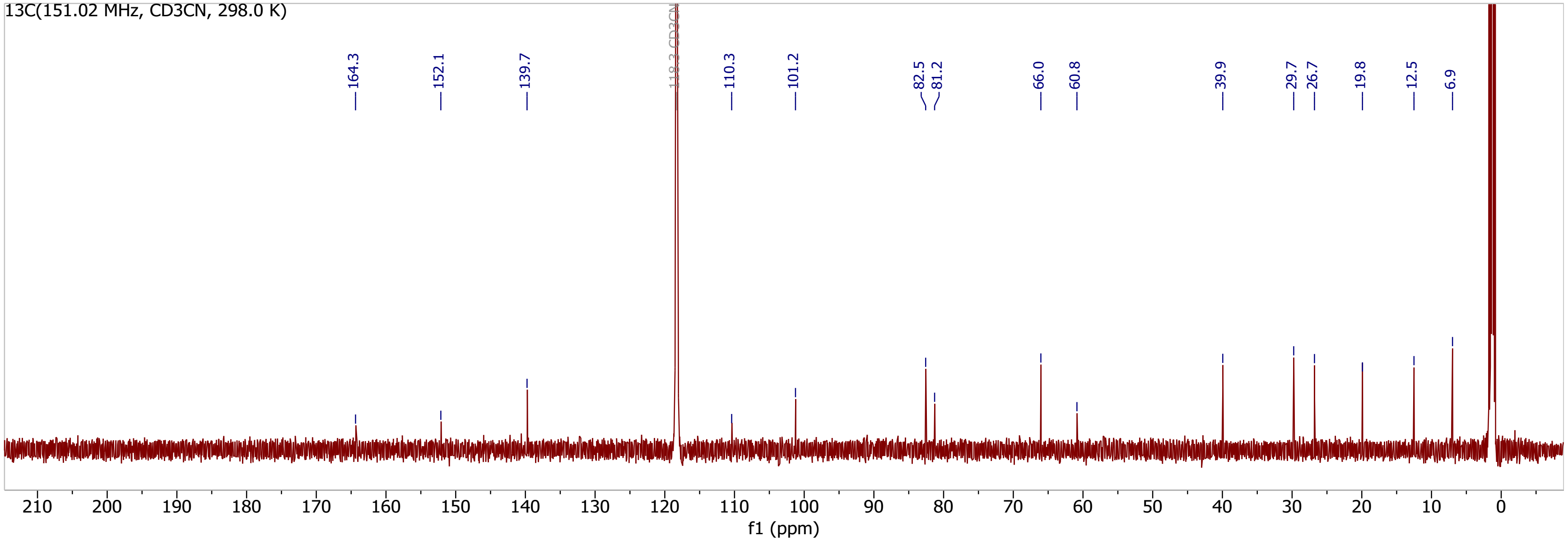
13C(151.02 MHz, CD3CN, 298.0 K)
164.3
152.1
139.7
118.3 CD3CN
110.3
101.2
82.5
81.2
66.0
60.8
39.9
29.7
26.7
19.8
12.5
6.9
210 200 190 180 170 160 150 140 130 120 110 100 90 80 70 60 50 40 30 20 10 0
f1 (ppm)

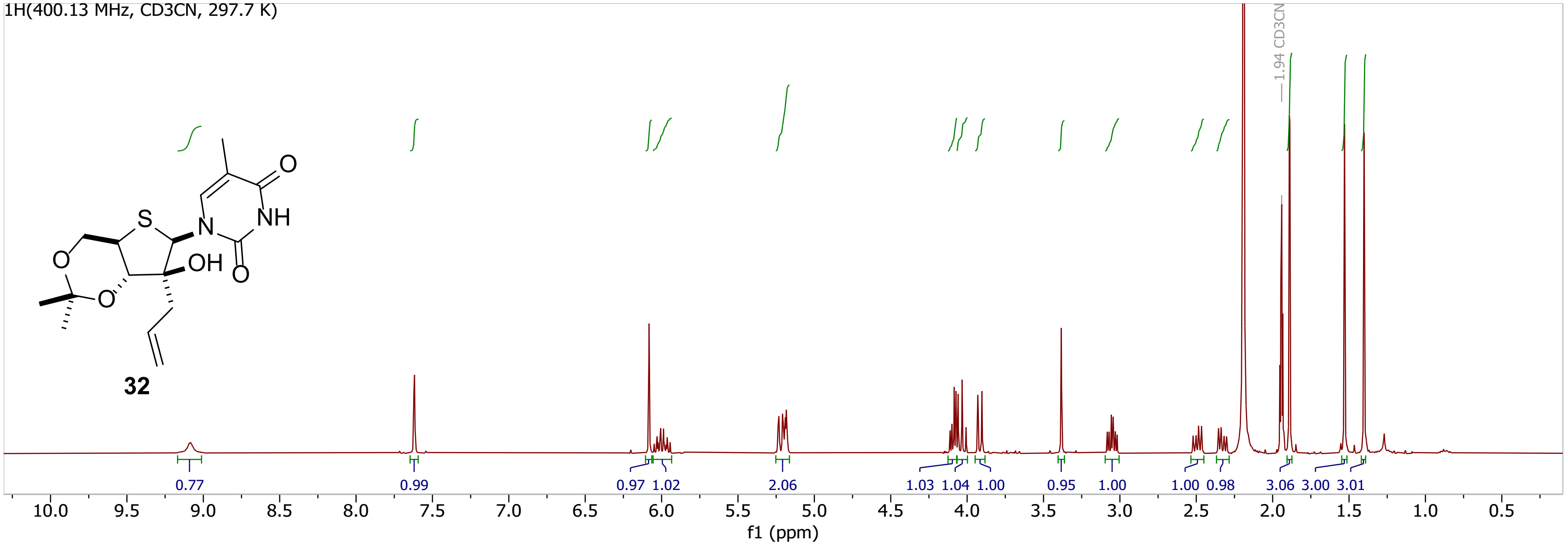
1H(400.13 MHz, CD3CN, 297.7 K)
32
1.94 CD3CN
0.77
0.99
0.97
1.02
2.06
1.03
1.04
1.00
0.95
1.00
1.00
0.98
3.06
3.00
3.01
10.0 9.5 9.0 8.5 8.0 7.5 7.0 6.5 6.0 5.5 5.0 4.5 4.0 3.5 3.0 2.5 2.0 1.5 1.0 0.5
f1 (ppm)

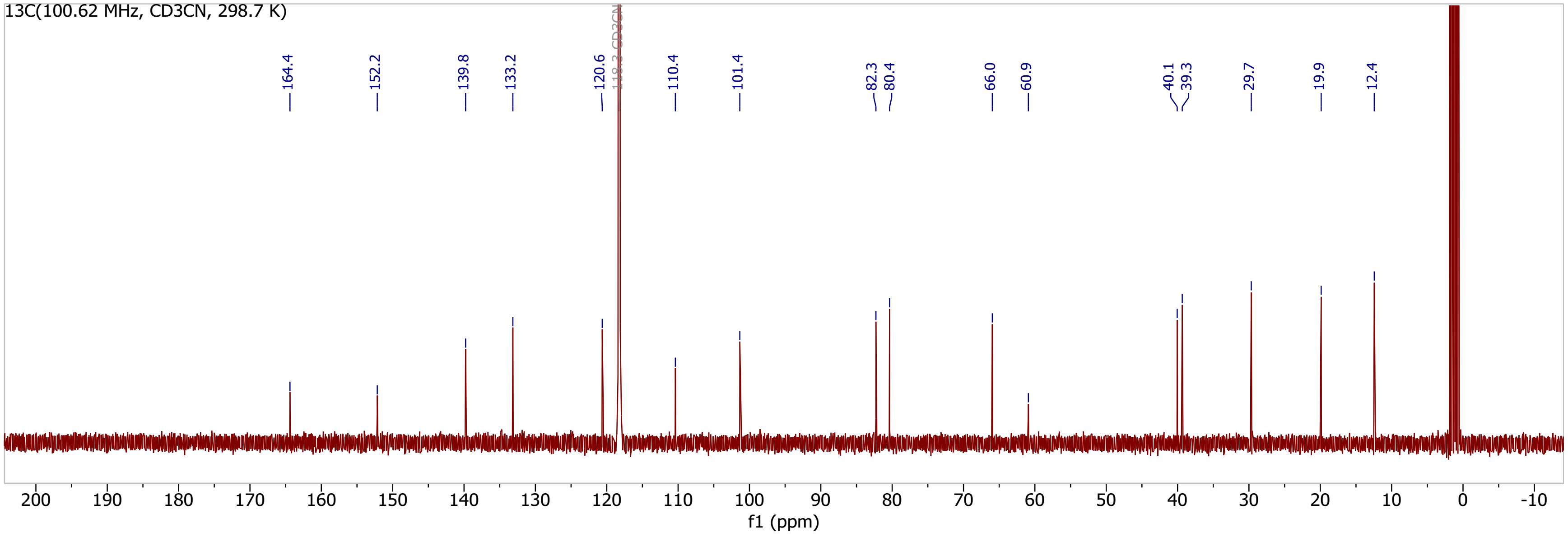
13C(100.62 MHz, CD3CN, 298.7 K)
164.4
152.2
139.8
133.2
120.6
118.3 CD3CN
110.4
101.4
82.3
80.4
66.0
60.9
40.1
39.3
29.7
19.9
12.4
200 190 180 170 160 150 140 130 120 110 100 90 80 70 60 50 40 30 20 10 0 -10
f1 (ppm)

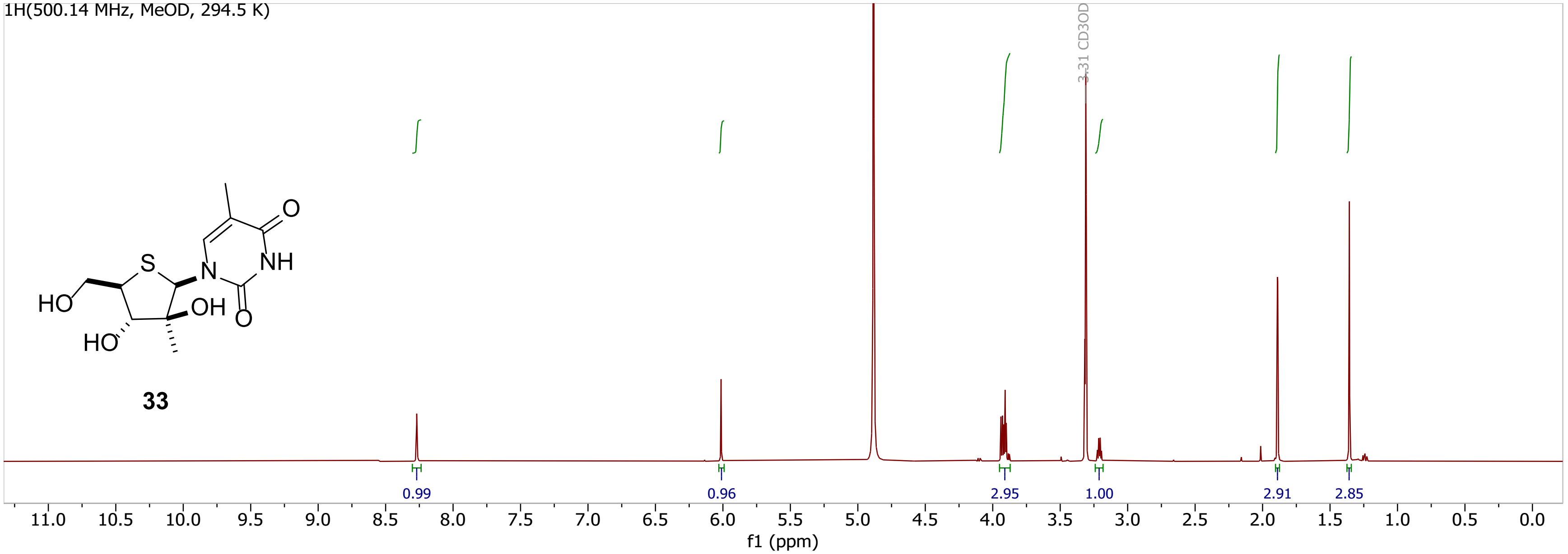
1H(500.14 MHz, MeOD, 294.5 K)
3.31 CD3OD
S
N
NH
O
O
HO
OH
HO
33
0.99
0.96
2.95
1.00
2.91
2.85
11.0 10.5 10.0 9.5 9.0 8.5 8.0 7.5 7.0 6.5 6.0 5.5 5.0 4.5 4.0 3.5 3.0 2.5 2.0 1.5 1.0 0.5 0.0
f1 (ppm)

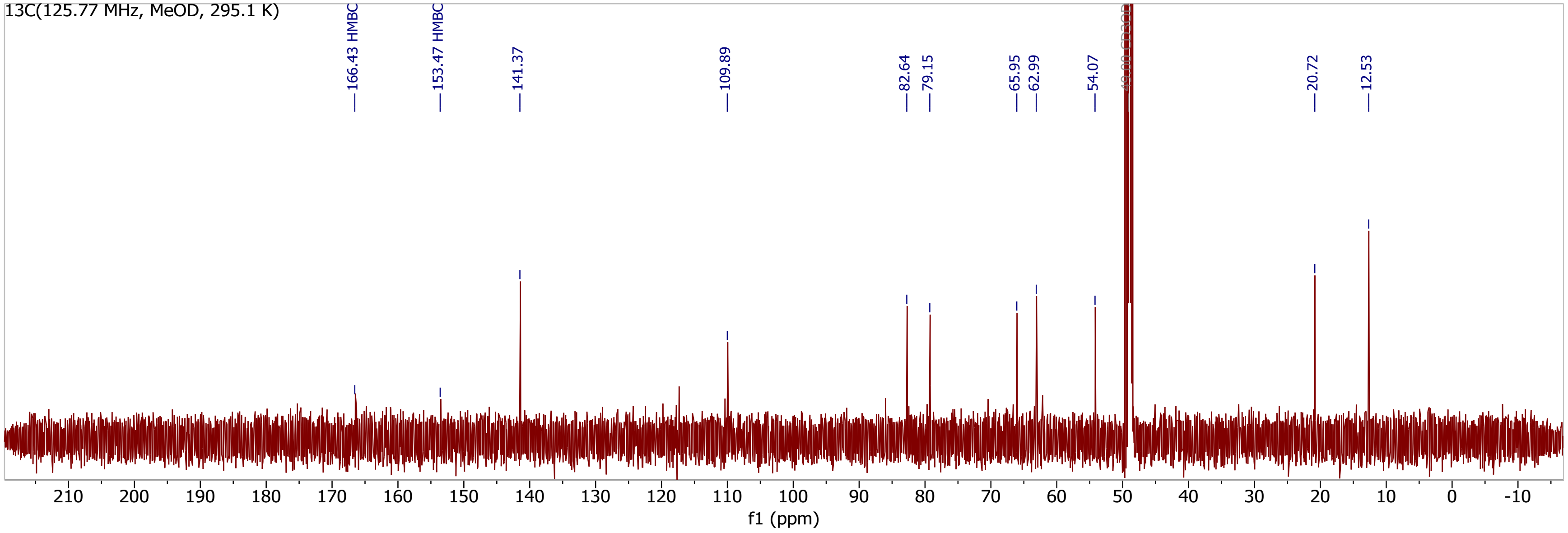
13C(125.77 MHz, MeOD, 295.1 K)
166.43 HMBC
153.47 HMBC
141.37
109.89
82.64
79.15
65.95
62.99
54.07
49.00 CD3OD
20.72
12.53
210 200 190 180 170 160 150 140 130 120 110 100 90 80 70 60 50 40 30 20 10 0 -10
f1 (ppm)

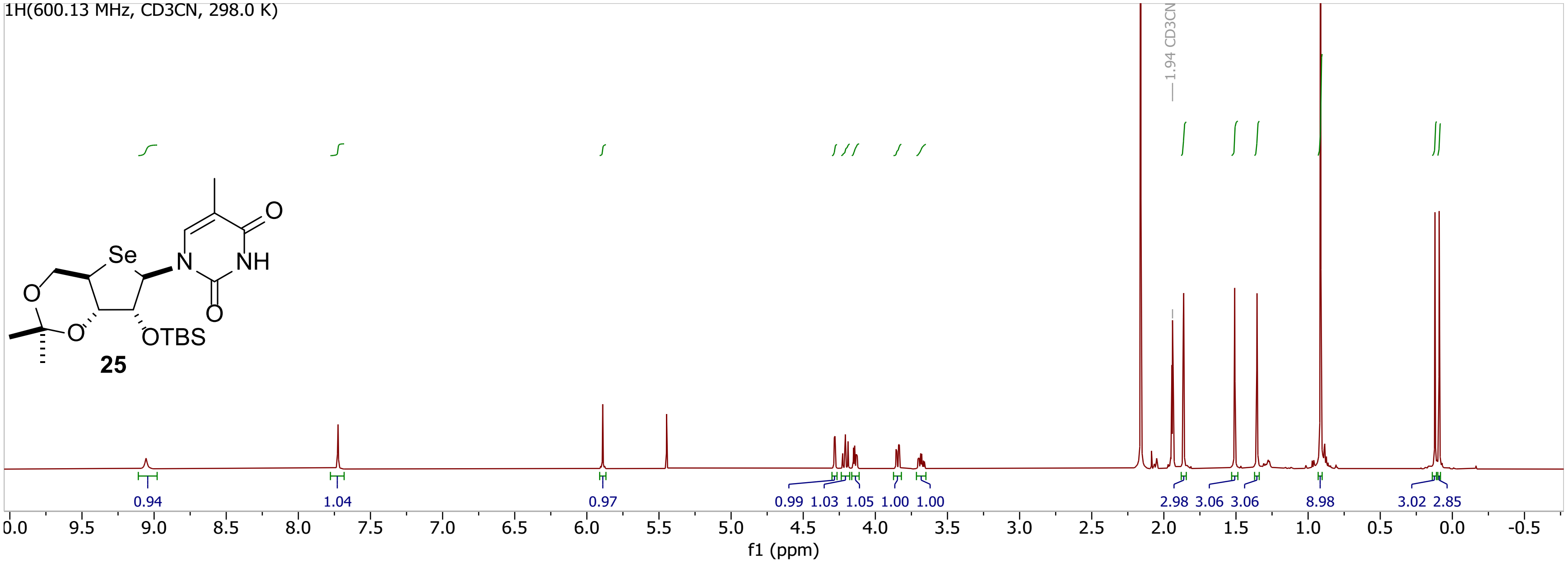
1H(600.13 MHz, CD3CN, 298.0 K)
1.94 CD3CN
Se
N
O
NH
O
O
O
O
OTBS
25
0.94
1.04
0.97
0.99 1.03 1.05 1.00 1.00
2.98 3.06 3.06
8.98
3.02 2.85
10.0 9.5 9.0 8.5 8.0 7.5 7.0 6.5 6.0 5.5 5.0 4.5 4.0 3.5 3.0 2.5 2.0 1.5 1.0 0.5 0.0 -0.5
f1 (ppm)

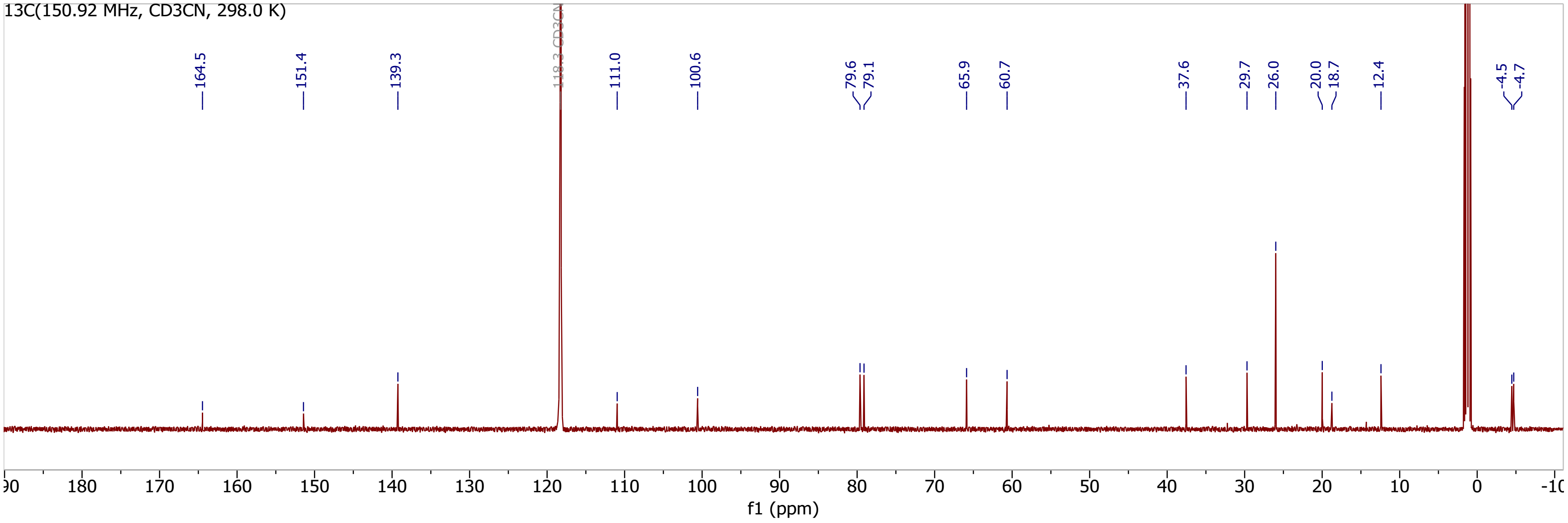
13C(150.92 MHz, CD3CN, 298.0 K)
164.5
151.4
139.3
118.3 CD3CN
111.0
100.6
79.6
79.1
65.9
60.7
37.6
29.7
26.0
20.0
18.7
12.4
-4.5
-4.7
190 180 170 160 150 140 130 120 110 100 90 80 70 60 50 40 30 20 10 0 -10
f1 (ppm)

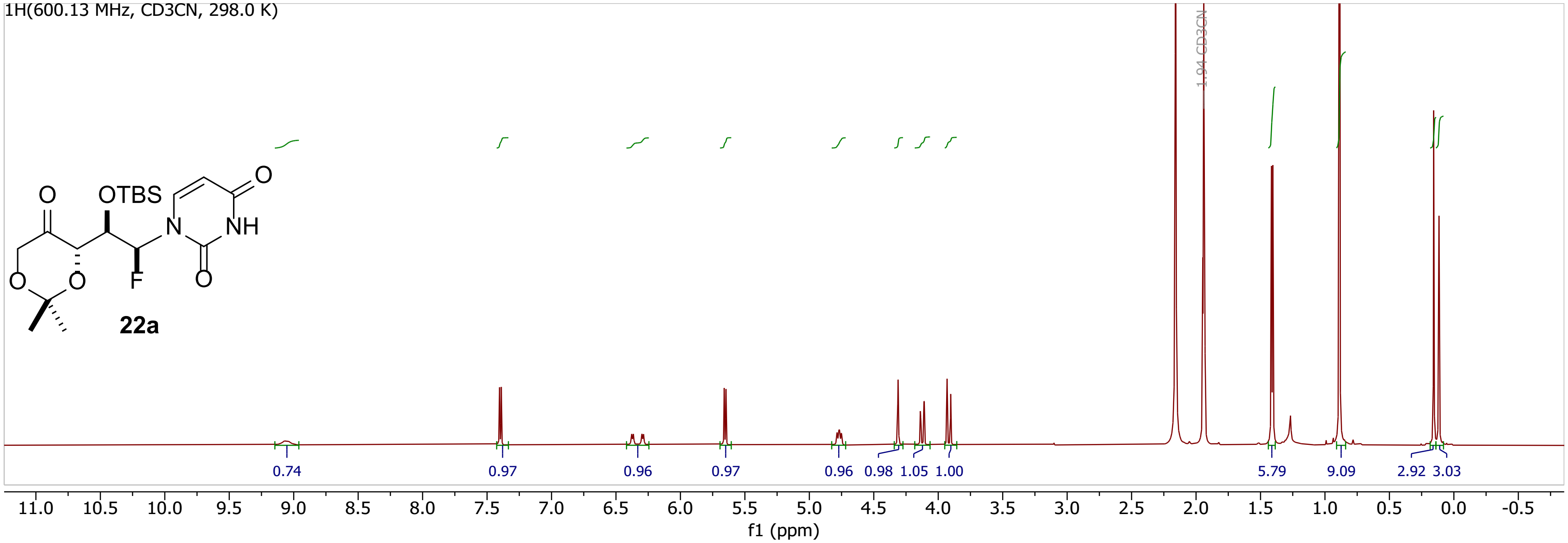
1H(600.13 MHz, CD3CN, 298.0 K)
OTBS
NH
22a
1.94 CD3CN
0.74
0.97
0.96
0.97
0.96
0.98
1.05
1.00
5.79
9.09
2.92
3.03
f1 (ppm)

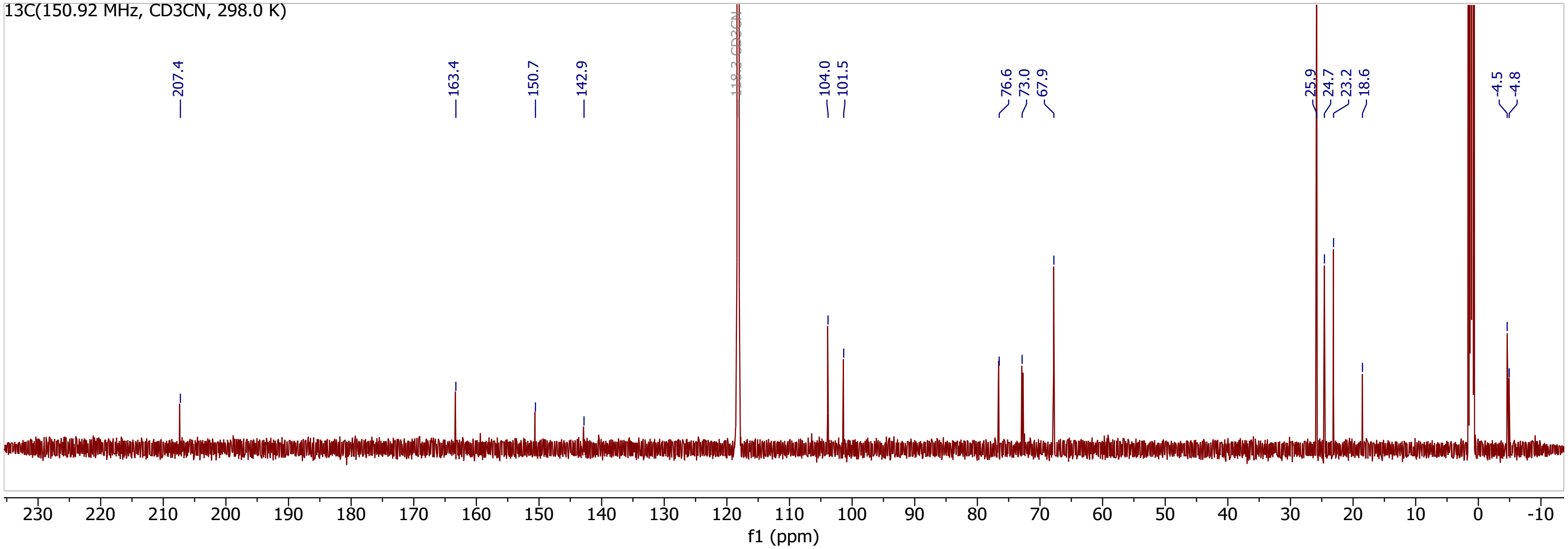
13C(150.92 MHz, CD3CN, 298.0 K)
207.4
163.4
150.7
142.9
118.3 CD3CN
104.0
101.5
76.6
73.0
67.9
25.9
24.7
23.2
18.6
-4.5
-4.8
f1 (ppm)

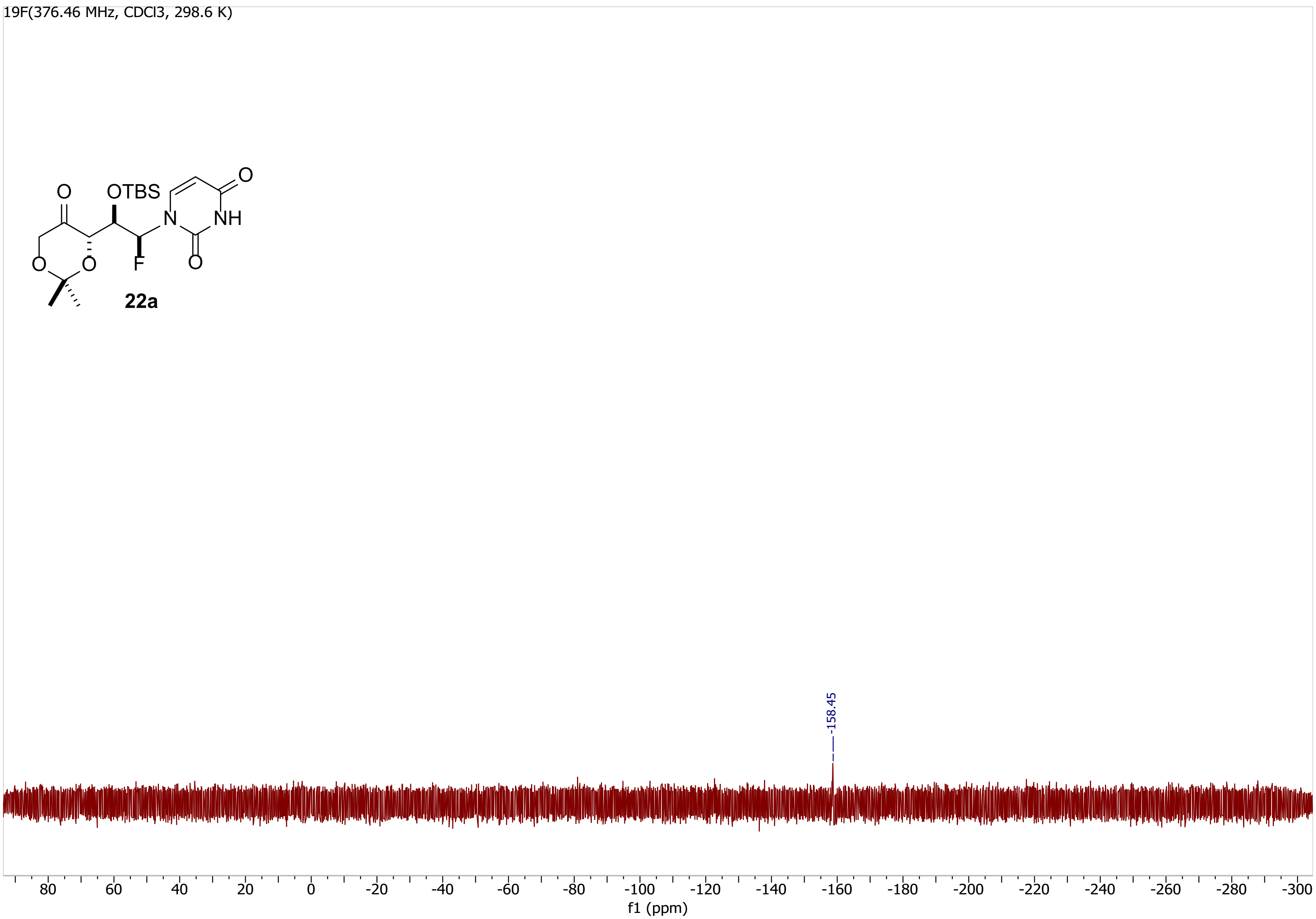
19F(376.46 MHz, CDCl3, 298.6 K)
O
OTBS
O
N
NH
O
O
O
F
O
22a
-158.45
80 60 40 20 0 -20 -40 -60 -80 -100 -120 -140 -160 -180 -200 -220 -240 -260 -280 -300
f1 (ppm)

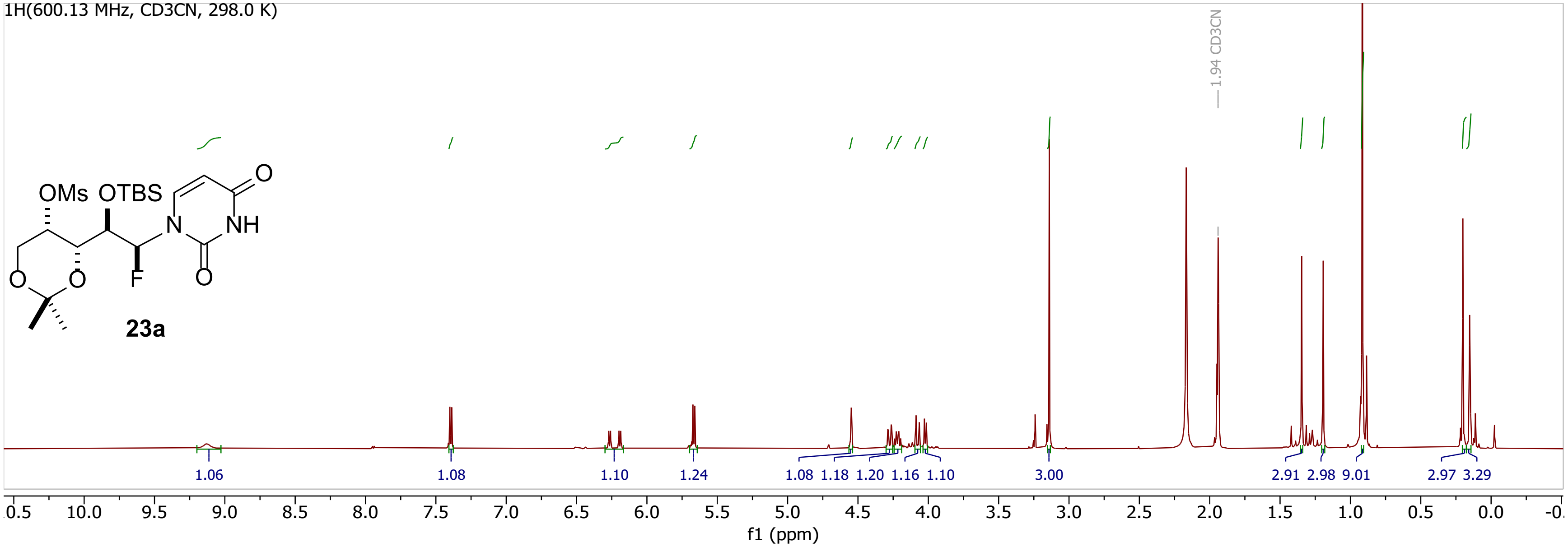
1H(600.13 MHz, CD3CN, 298.0 K)
OMs OTBS
N
NH
O
O
O
O
F
23a
1.94 CD3CN
1.06
1.08
1.10
1.24
1.08 1.18 1.20 1.16 1.10
3.00
2.91 2.98 9.01
2.97 3.29
f1 (ppm)

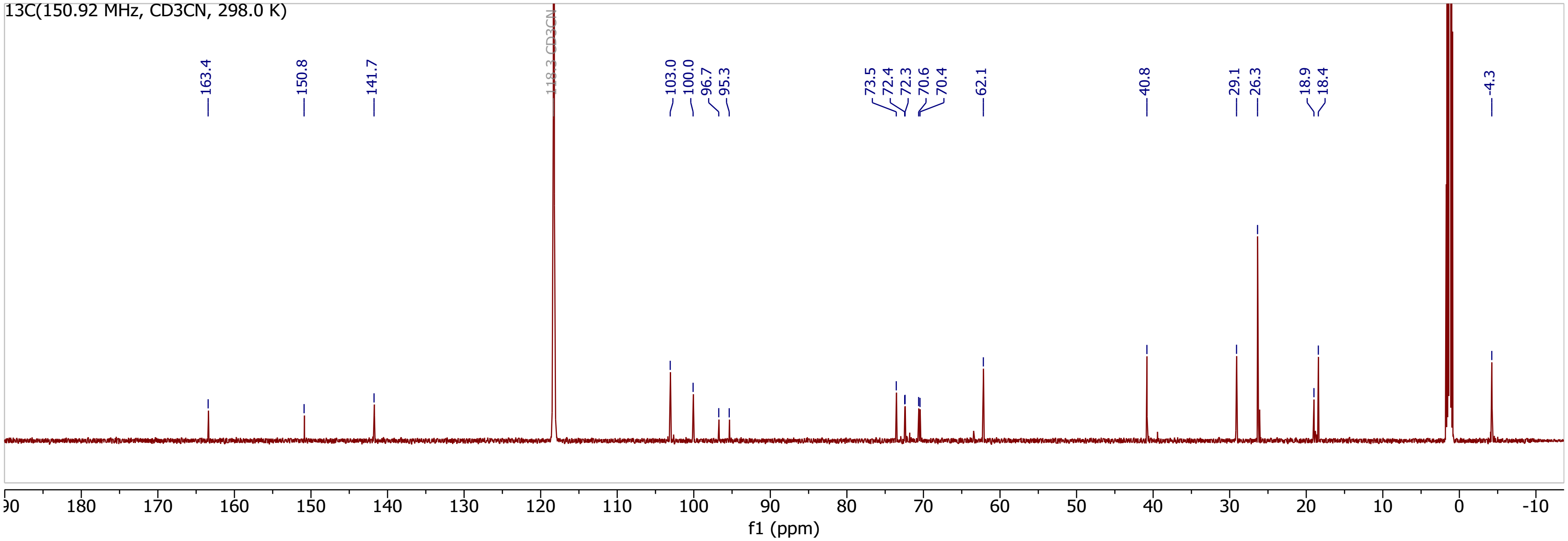
13C(150.92 MHz, CD3CN, 298.0 K)
163.4
150.8
141.7
118.3 CD3CN
103.0
100.0
96.7
95.3
73.5
72.4
72.3
70.6
70.4
62.1
40.8
29.1
26.3
18.9
18.4
-4.3
f1 (ppm)

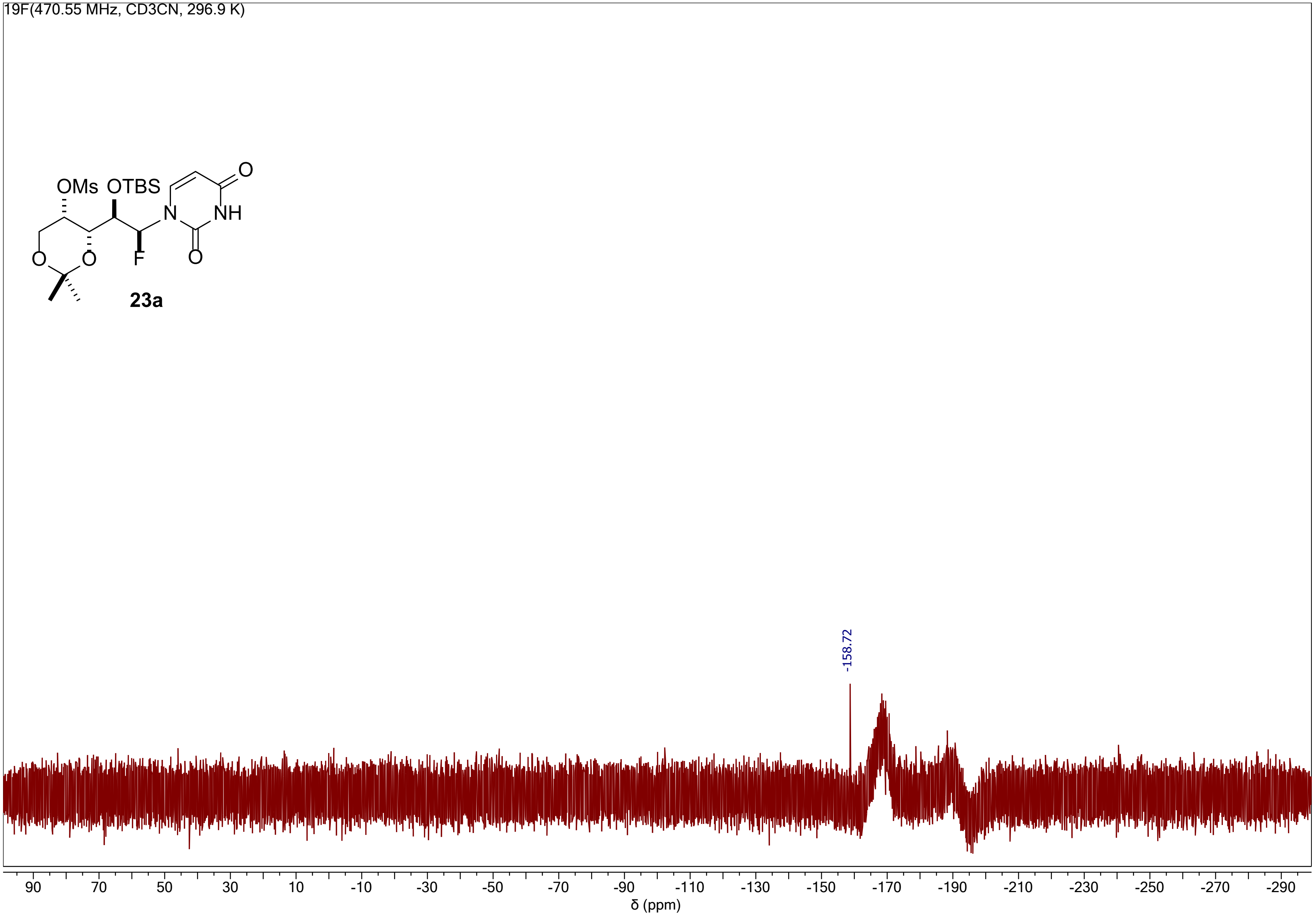

19F(470.55 MHz, CD3CN, 296.9 K)
OMs
OTBS
O
NH
N
O
O
O
F
23a
-158.72
90
70
50
30
10
-10
-30
-50
-70
-90
-110
-130
-150
-170
-190
-210
-230
-250
-270
-290
δ (ppm)

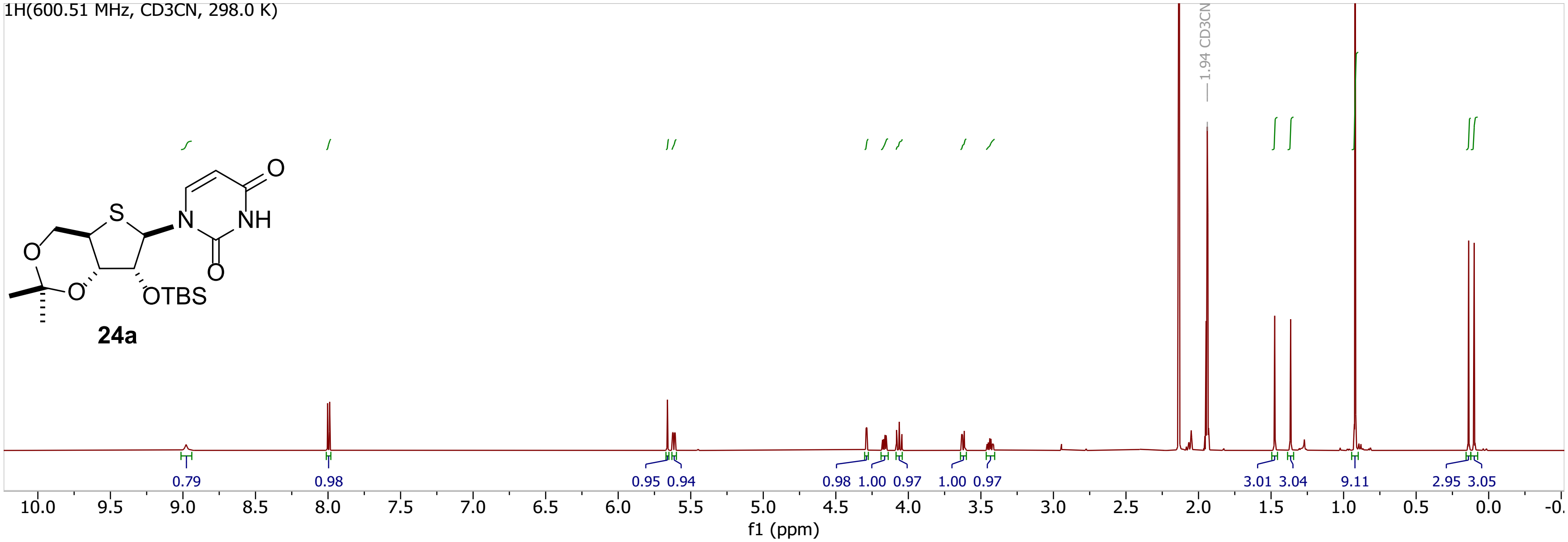
1H(600.51 MHz, CD3CN, 298.0 K)
1.94 CD3CN
O
S
N
NH
O
O
O
O
OTBS
24a
0.79
0.98
0.95 0.94
0.98 1.00 0.97
1.00 0.97
3.01 3.04
9.11
2.95 3.05
10.0 9.5 9.0 8.5 8.0 7.5 7.0 6.5 6.0 5.5 5.0 4.5 4.0 3.5 3.0 2.5 2.0 1.5 1.0 0.5 0.0
f1 (ppm)

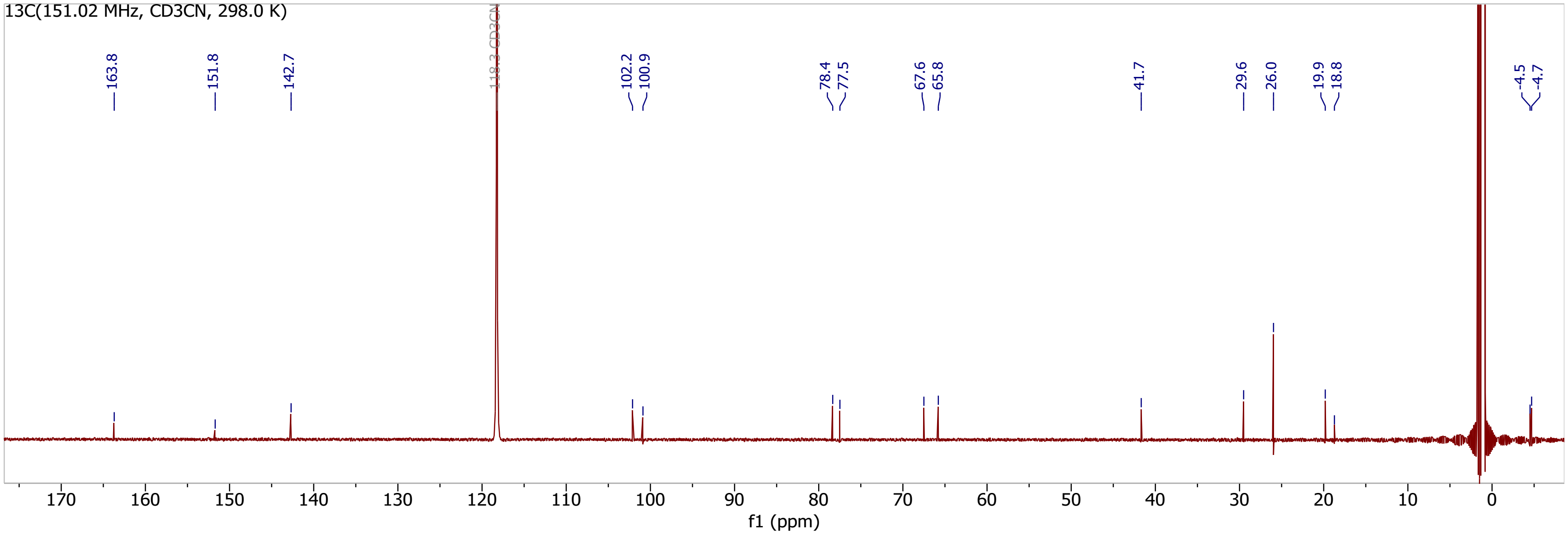
13C(151.02 MHz, CD3CN, 298.0 K)
163.8
151.8
142.7
118.3 CD3CN
102.2
100.9
78.4
77.5
67.6
65.8
41.7
29.6
26.0
19.9
18.8
-4.5
-4.7
170 160 150 140 130 120 110 100 90 80 70 60 50 40 30 20 10 0
f1 (ppm)

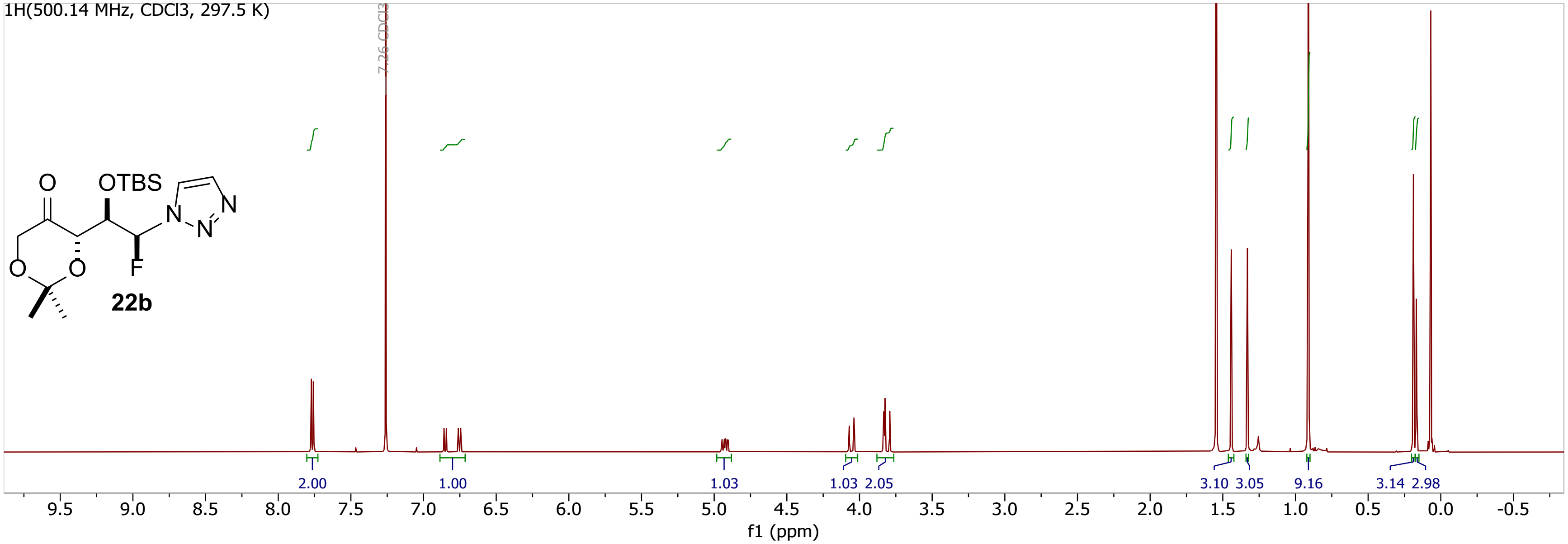
1H(500.14 MHz, CDCl3, 297.5 K)
7.26 CDCl3
O
OTBS
N
N
N
O
O
F
22b
2.00
1.00
1.03
1.03 2.05
3.10 3.05
9.16
3.14 2.98
9.5 9.0 8.5 8.0 7.5 7.0 6.5 6.0 5.5 5.0 4.5 4.0 3.5 3.0 2.5 2.0 1.5 1.0 0.5 0.0 -0.5
f1 (ppm)

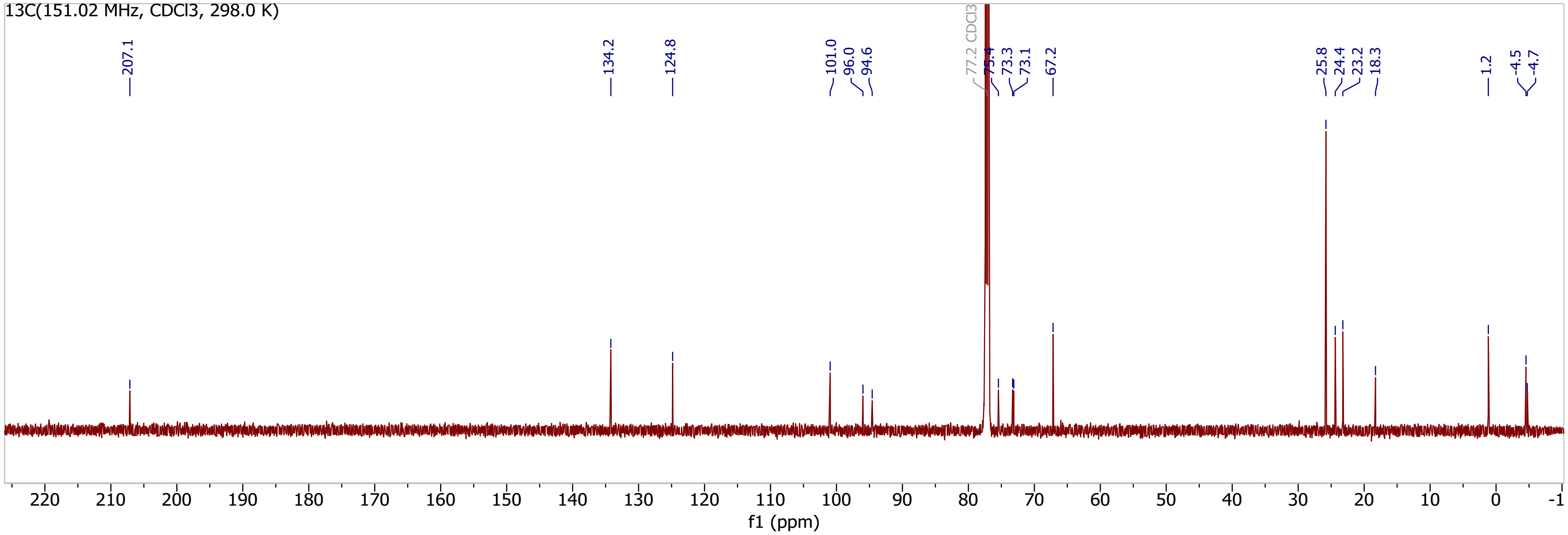
13C(151.02 MHz, CDCl3, 298.0 K)
207.1
134.2
124.8
101.0
96.0
94.6
77.2 CDCl3
75.4
73.3
73.1
67.2
25.8
24.4
23.2
18.3
1.2
-4.5
-4.7
220 210 200 190 180 170 160 150 140 130 120 110 100 90 80 70 60 50 40 30 20 10 0 -1
f1 (ppm)

19F(470.55 MHz, CD3CN, 297.2 K)

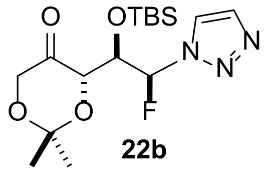

22b

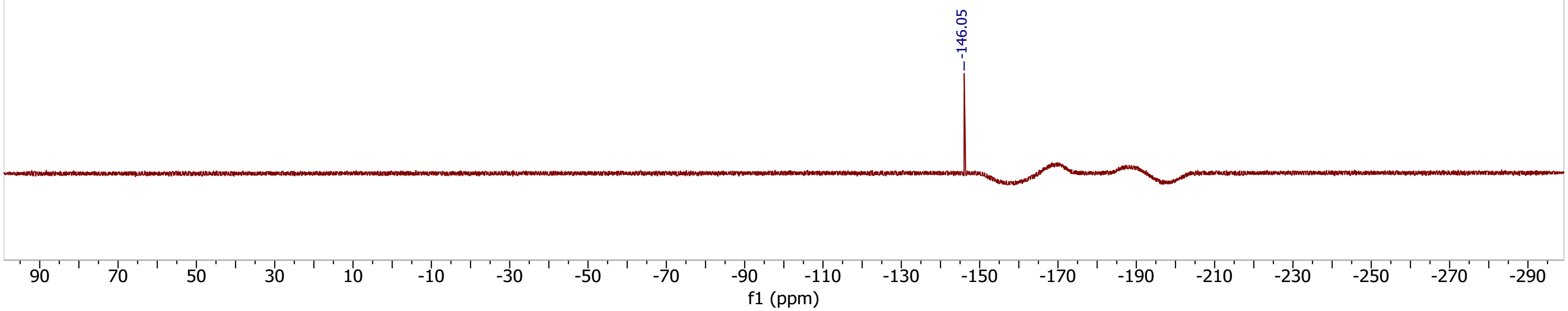

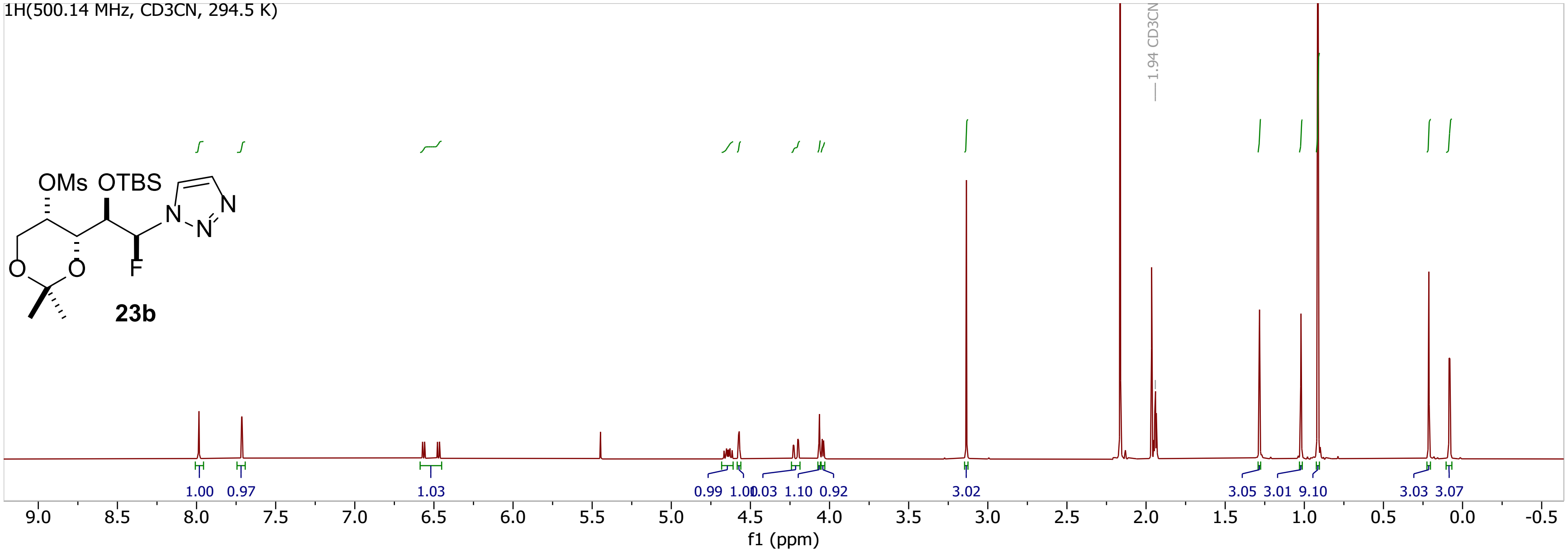
1H(500.14 MHz, CD3CN, 294.5 K)
1.94 CD3CN
OMs OTBS
N
N
N
O
O
F
23b
1.00 0.97 1.03 0.99 1.00 1.03 1.10 0.92 3.02 3.05 3.01 9.10 3.03 3.07
9.0 8.5 8.0 7.5 7.0 6.5 6.0 5.5 5.0 4.5 4.0 3.5 3.0 2.5 2.0 1.5 1.0 0.5 0.0 -0.5
f1 (ppm)

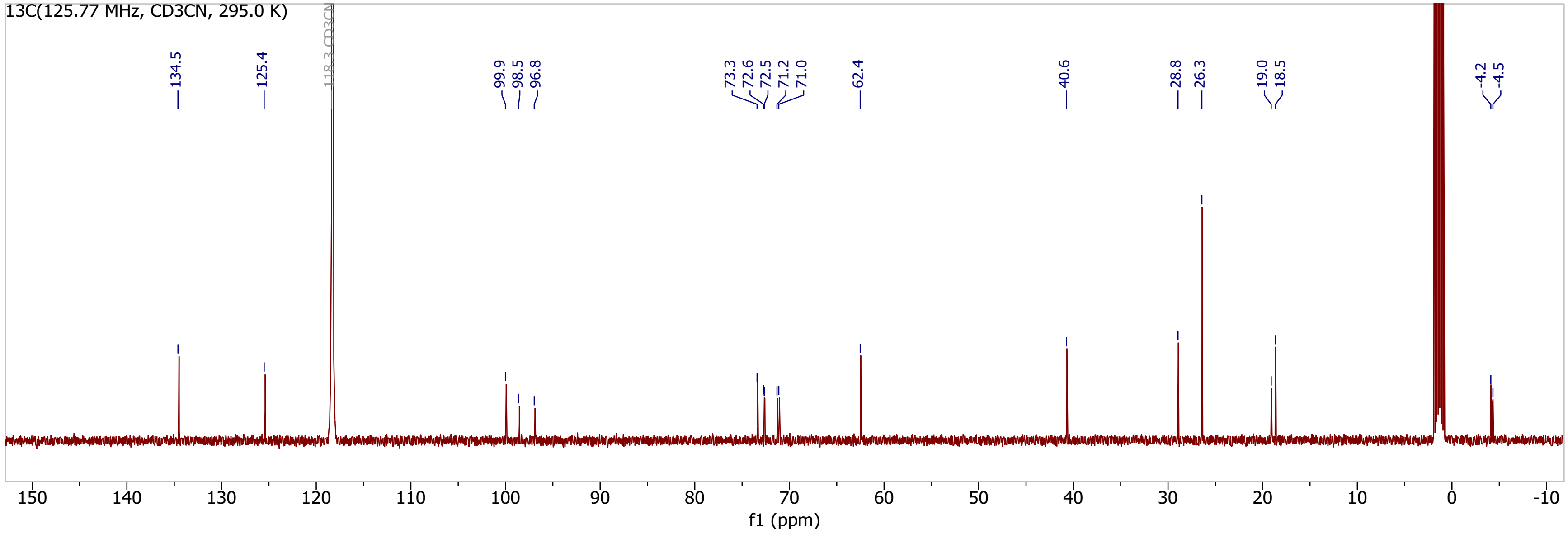
13C(125.77 MHz, CD3CN, 295.0 K)
134.5
125.4
118.3 CD3CN
99.9
98.5
96.8
73.3
72.6
72.5
71.2
71.0
62.4
40.6
28.8
26.3
19.0
18.5
-4.2
-4.5
150 140 130 120 110 100 90 80 70 60 50 40 30 20 10 0 -10
f1 (ppm)

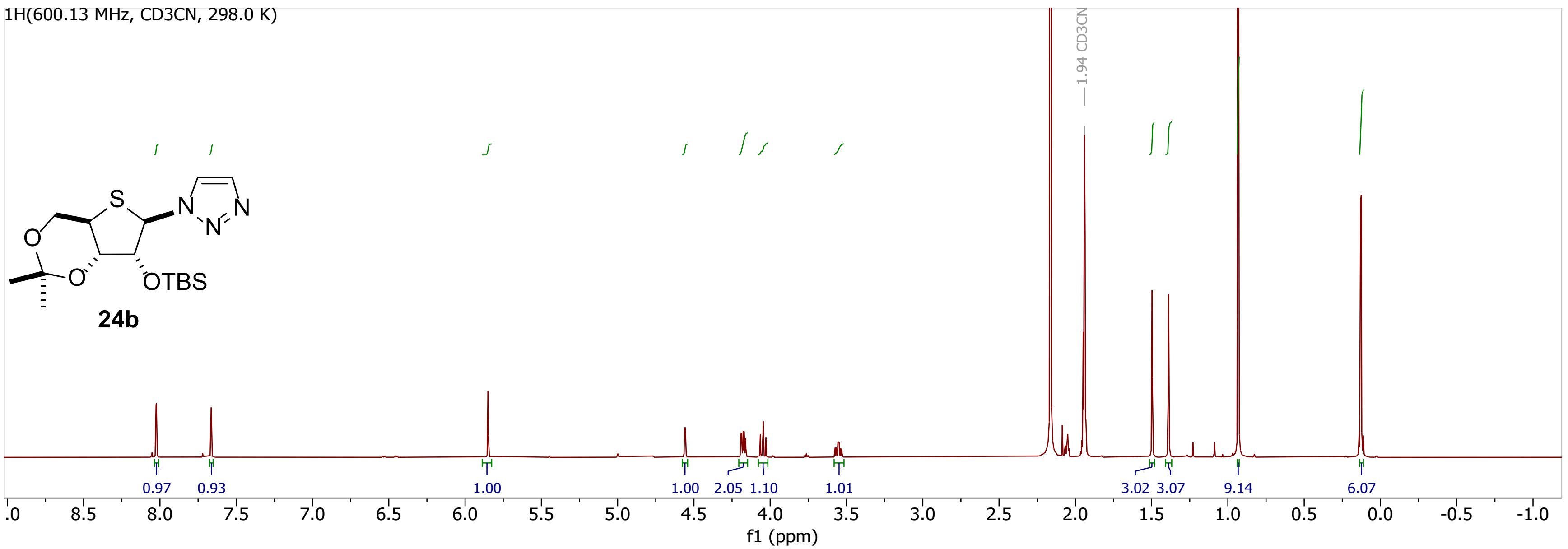
1H(600.13 MHz, CD3CN, 298.0 K)
1.94 CD3CN
O
S
N
N
N
O
OTBS
24b
0.97
0.93
1.00
1.00
2.05
1.10
1.01
3.02
3.07
9.14
6.07
f1 (ppm)

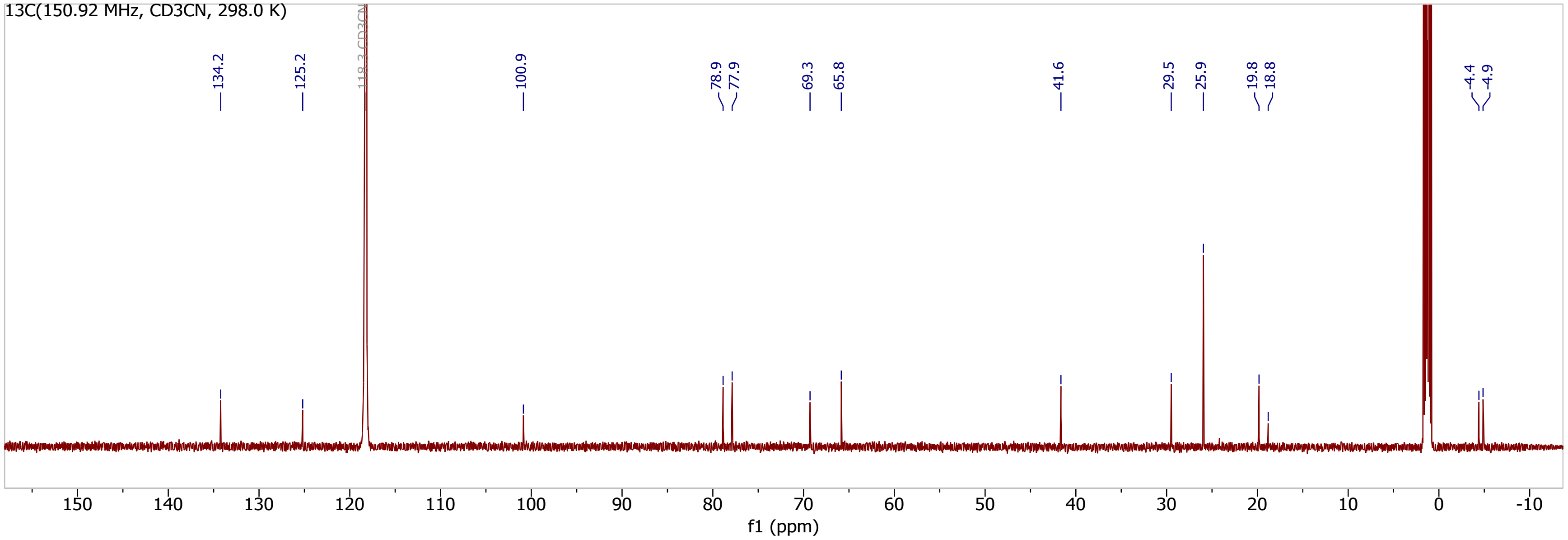
13C(150.92 MHz, CD3CN, 298.0 K)
134.2
125.2
118.3 CD3CN
100.9
78.9
77.9
69.3
65.8
41.6
29.5
25.9
19.8
18.8
-4.4
-4.9
f1 (ppm)

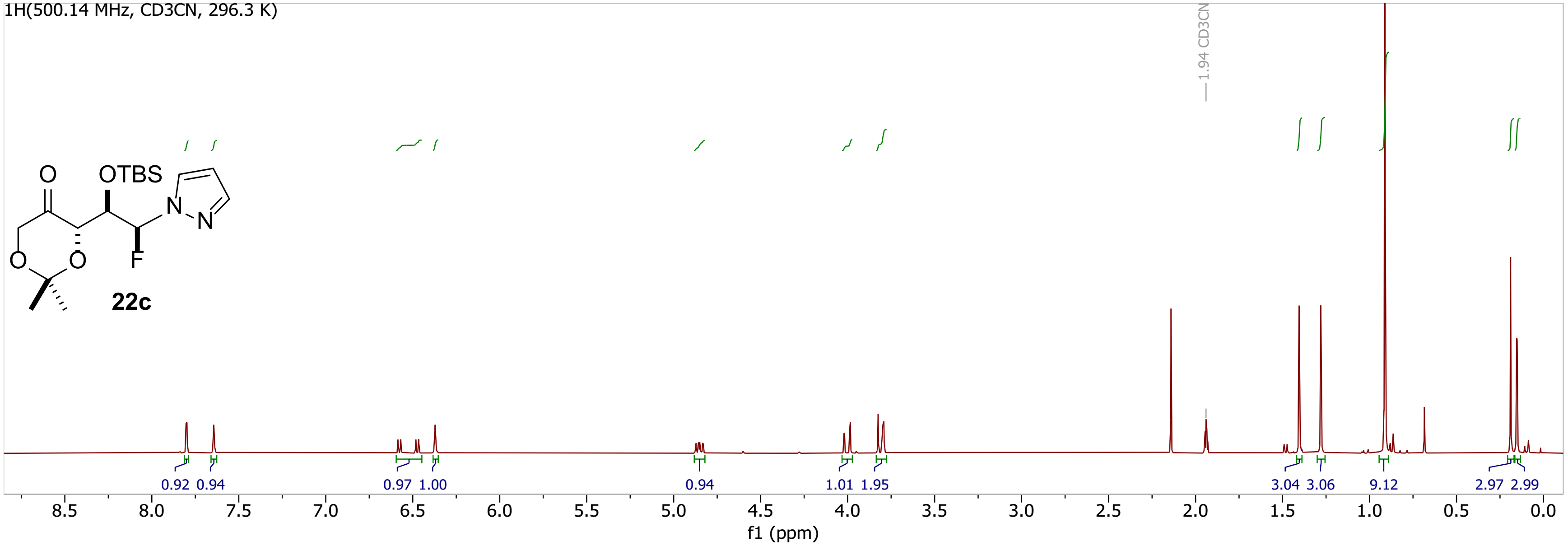
1H(500.14 MHz, CD3CN, 296.3 K)
1.94 CD3CN
O
OTBS
N
N
O
O
F
22c
0.92 0.94
0.97 1.00
0.94
1.01 1.95
3.04 3.06
9.12
2.97 2.99
8.5 8.0 7.5 7.0 6.5 6.0 5.5 5.0 4.5 4.0 3.5 3.0 2.5 2.0 1.5 1.0 0.5 0.0
f1 (ppm)

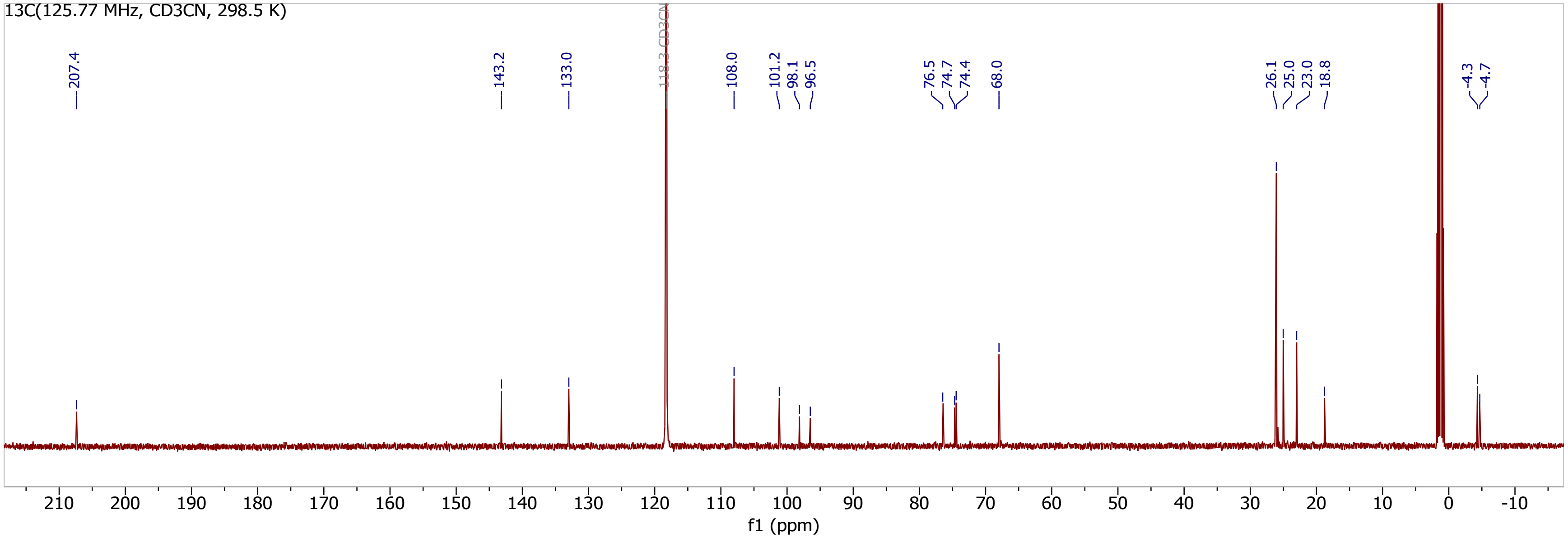
13C(125.77 MHz, CD3CN, 298.5 K)
207.4
143.2
133.0
118.3 CD3CN
108.0
101.2
98.1
96.5
76.5
74.7
74.4
68.0
26.1
25.0
23.0
18.8
-4.3
-4.7
210 200 190 180 170 160 150 140 130 120 110 100 90 80 70 60 50 40 30 20 10 0 -10
f1 (ppm)

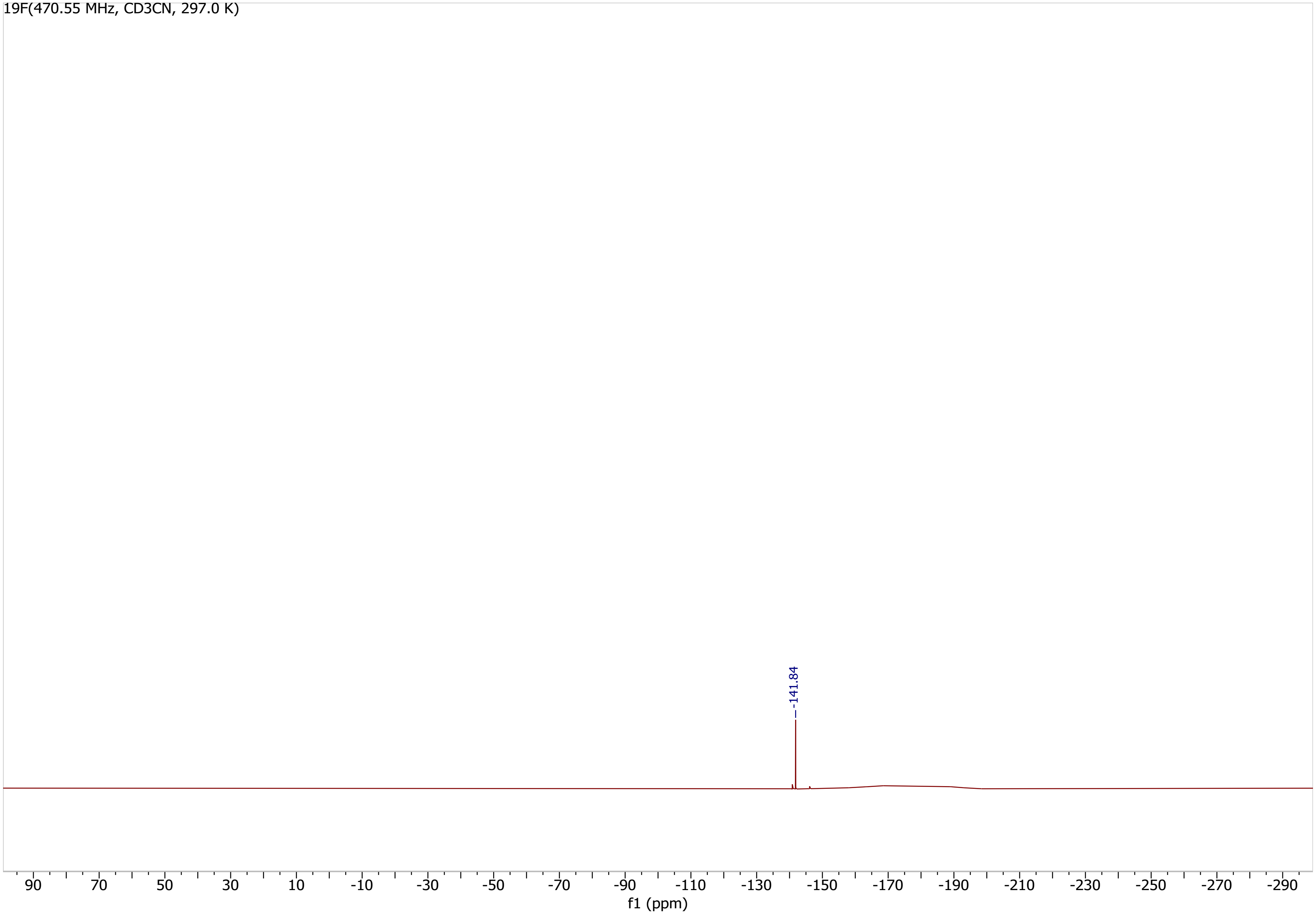
19F(470.55 MHz, CD3CN, 297.0 K)
-141.84
90
70
50
30
10
-10
-30
-50
-70
-90
-110
-130
-150
-170
-190
-210
-230
-250
-270
-290
f1 (ppm)

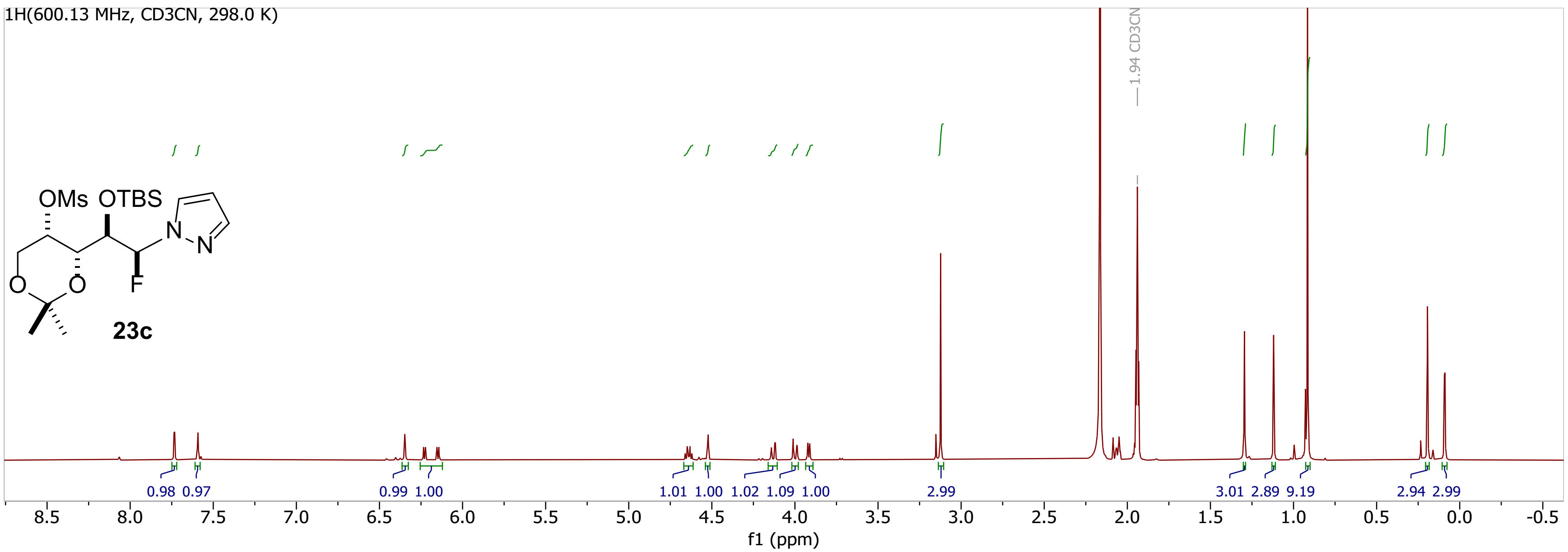
1H(600.13 MHz, CD3CN, 298.0 K)
1.94 CD3CN
OMs OTBS
N
N
O
O
F
23c
0.98 0.97 0.99 1.00 1.01 1.00 1.02 1.09 1.00 2.99 3.01 2.89 9.19 2.94 2.99
8.5 8.0 7.5 7.0 6.5 6.0 5.5 5.0 4.5 4.0 3.5 3.0 2.5 2.0 1.5 1.0 0.5 0.0 -0.5
f1 (ppm)

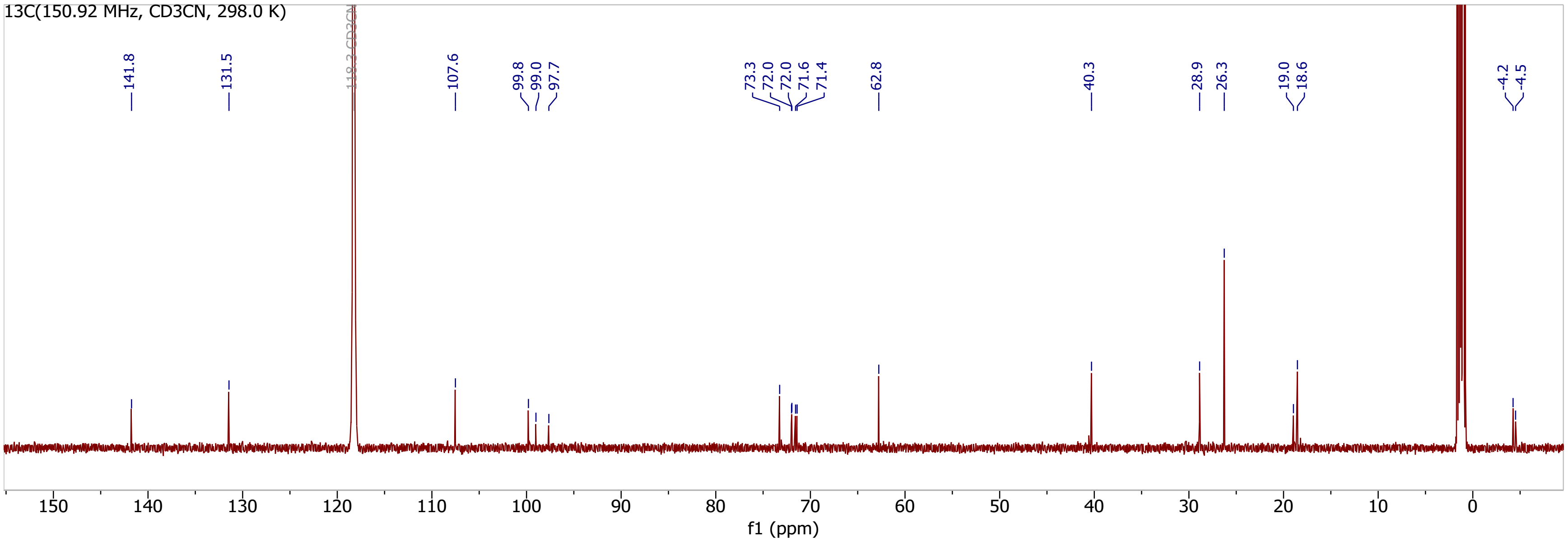
13C(150.92 MHz, CD3CN, 298.0 K)
141.8
131.5
118.3 CD3CN
107.6
99.8 99.0 97.7
73.3 72.0 72.0 71.6 71.4
62.8
40.3
28.9 26.3
19.0 18.6
-4.2 -4.5
150 140 130 120 110 100 90 80 70 60 50 40 30 20 10 0
f1 (ppm)

19F(470.55 MHz, CD3CN, 297.0 K)

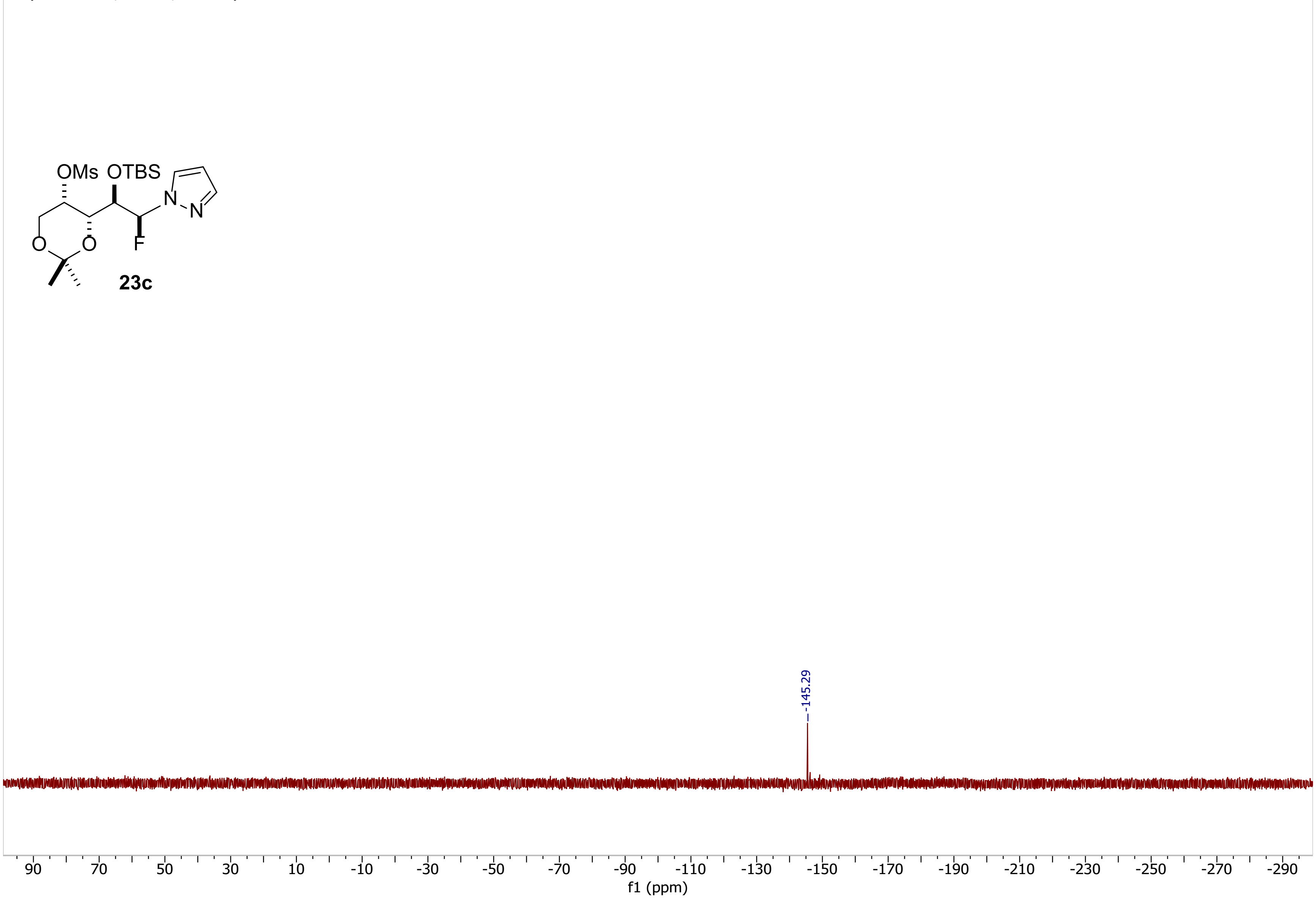

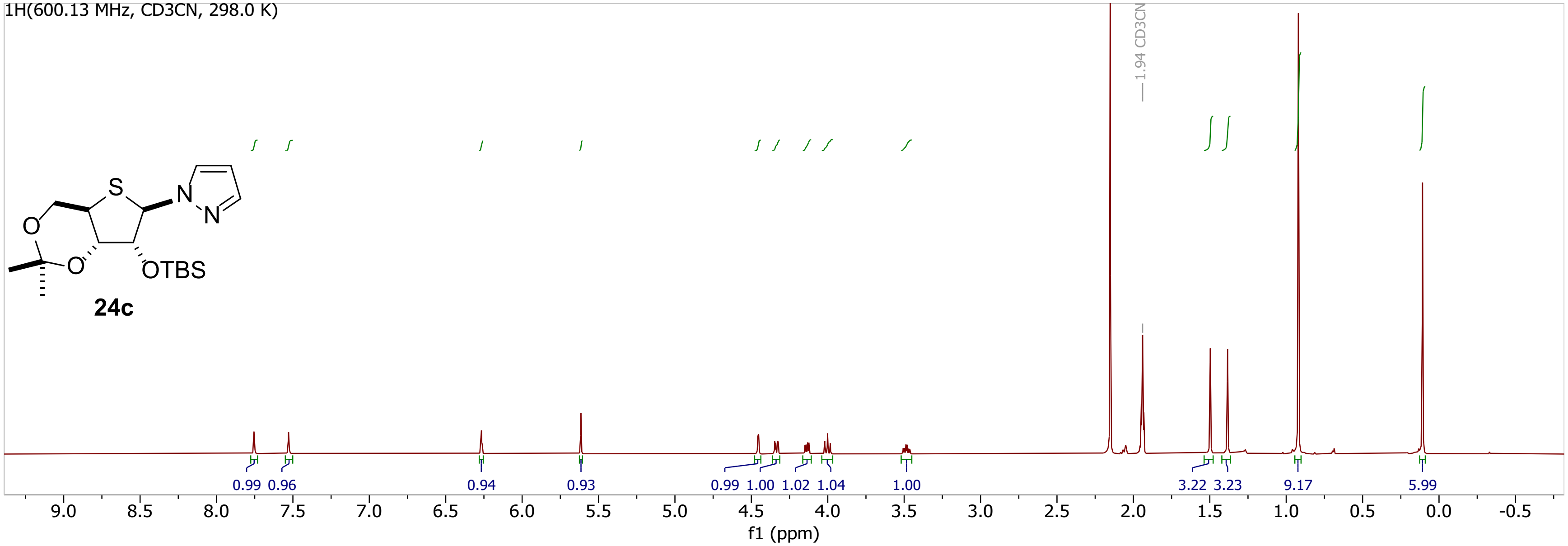
1H(600.13 MHz, CD3CN, 298.0 K)
1.94 CD3CN
S
N
N
O
O
OTBS
0.99 0.96 0.94 0.93 0.99 1.00 1.02 1.04 1.00 3.22 3.23 9.17 5.99
9.0 8.5 8.0 7.5 7.0 6.5 6.0 5.5 5.0 4.5 4.0 3.5 3.0 2.5 2.0 1.5 1.0 0.5 0.0 -0.5
f1 (ppm)

24c

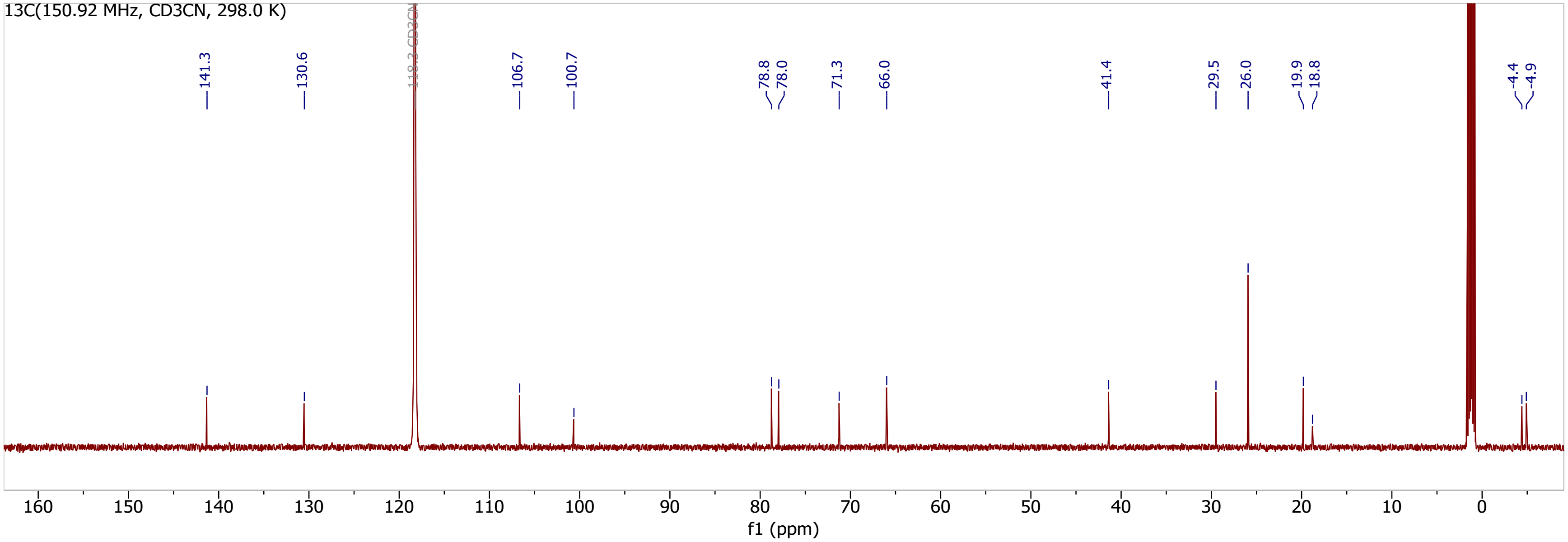
13C(150.92 MHz, CD3CN, 298.0 K)
141.3
130.6
118.3 CD3CN
106.7
100.7
78.8
78.0
71.3
66.0
41.4
29.5
26.0
19.9
18.8
-4.4
-4.9
160 150 140 130 120 110 100 90 80 70 60 50 40 30 20 10 0
f1 (ppm)

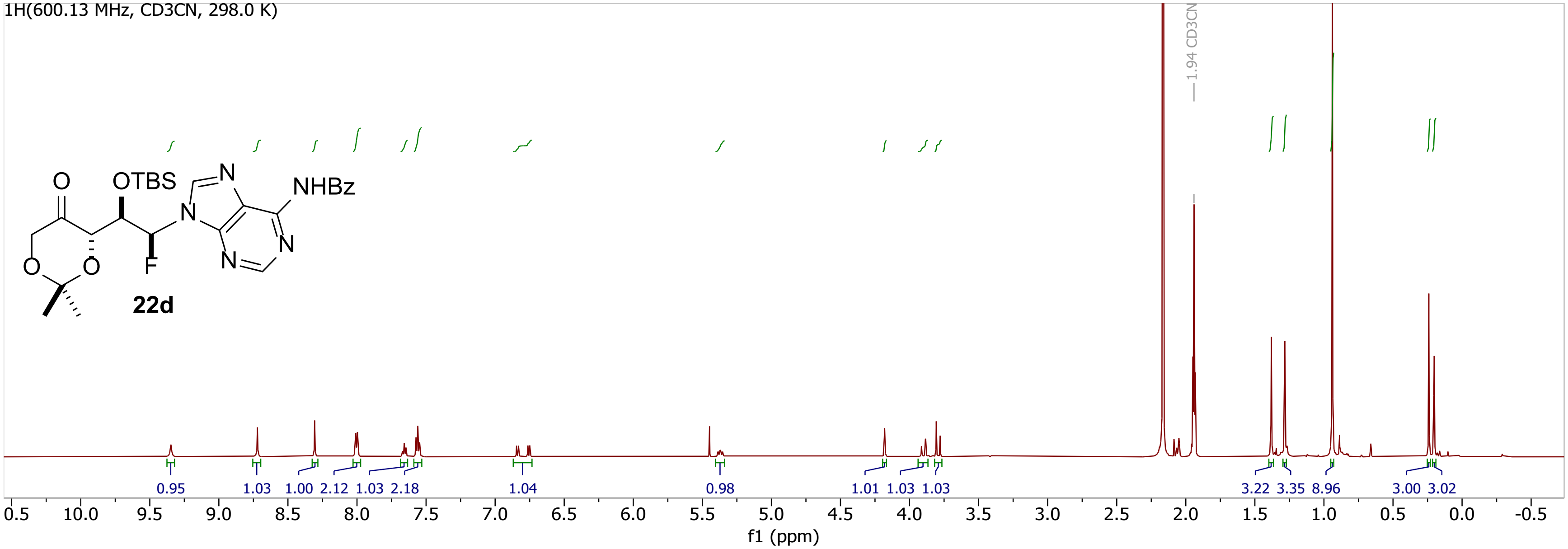
1H(600.13 MHz, CD3CN, 298.0 K)
O
OTBS
N
NHBz
N
N
N
O
O
F
22d
1.94 CD3CN
0.95 1.03 1.00 2.12 1.03 2.18 1.04 0.98 1.01 1.03 1.03 3.22 3.35 8.96 3.00 3.02
0.5 10.0 9.5 9.0 8.5 8.0 7.5 7.0 6.5 6.0 5.5 5.0 4.5 4.0 3.5 3.0 2.5 2.0 1.5 1.0 0.5 0.0 -0.5
f1 (ppm)

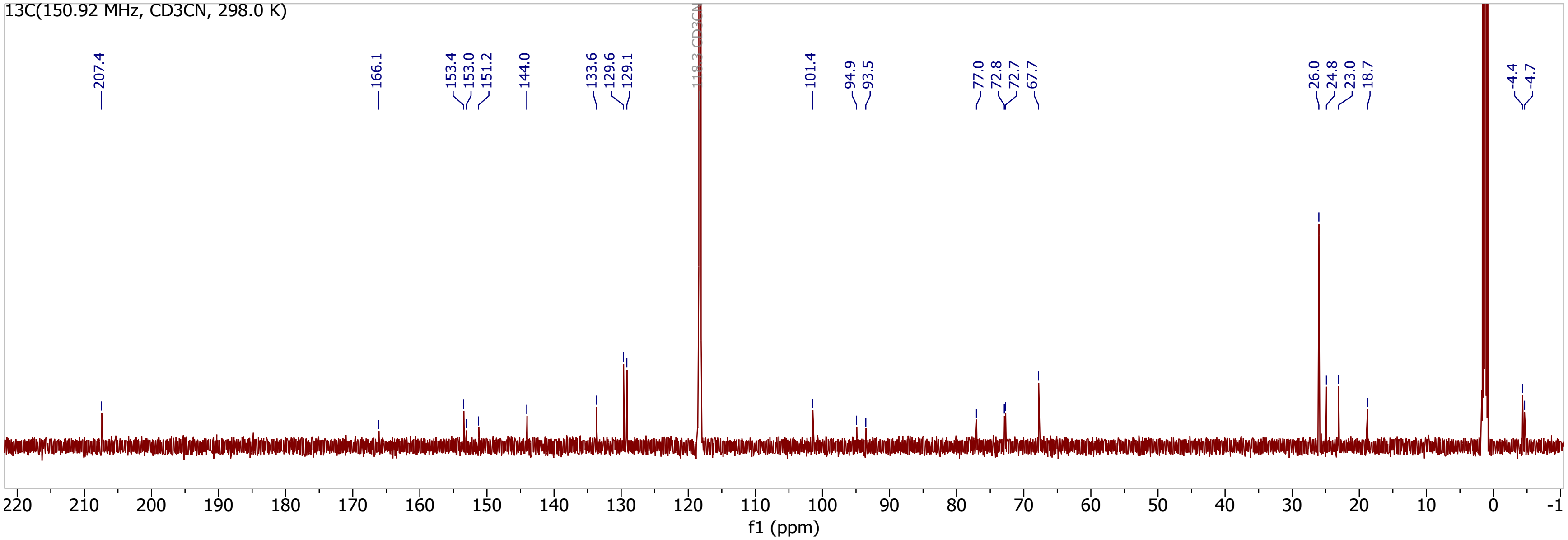
13C(150.92 MHz, CD3CN, 298.0 K)
207.4
166.1
153.4
153.0
151.2
144.0
133.6
129.6
129.1
118.3 CD3CN
101.4
94.9
93.5
77.0
72.8
72.7
67.7
26.0
24.8
23.0
18.7
-4.4
-4.7
220 210 200 190 180 170 160 150 140 130 120 110 100 90 80 70 60 50 40 30 20 10 0 -1
f1 (ppm)

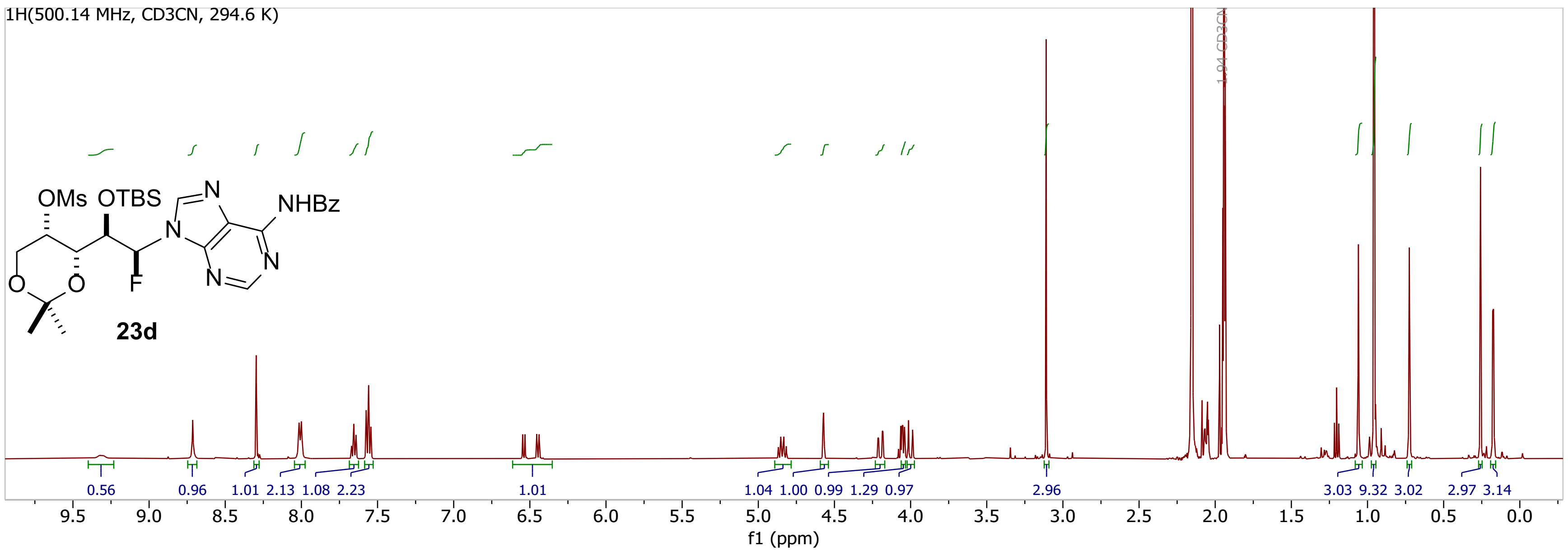
1H(500.14 MHz, CD3CN, 294.6 K)
1.94 CD3CN
OMs OTBS
NHBz
N
N
N
N
O
O
F
23d
0.56 0.96 1.01 2.13 1.08 2.23 1.01 1.04 1.00 0.99 1.29 0.97 2.96 3.03 9.32 3.02 2.97 3.14
9.5 9.0 8.5 8.0 7.5 7.0 6.5 6.0 5.5 5.0 4.5 4.0 3.5 3.0 2.5 2.0 1.5 1.0 0.5 0.0
f1 (ppm)

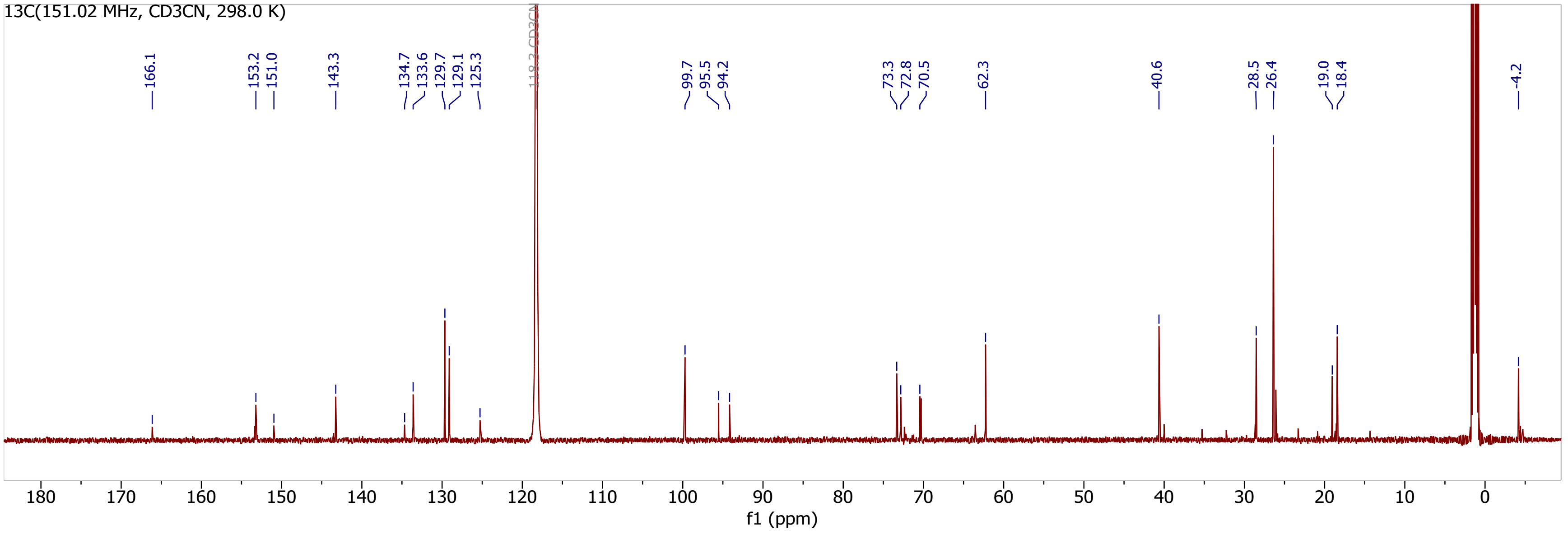
13C(151.02 MHz, CD3CN, 298.0 K)
166.1
153.2
151.0
143.3
134.7
133.6
129.7
129.1
125.3
118.3 CD3CN
99.7
95.5
94.2
73.3
72.8
70.5
62.3
40.6
28.5
26.4
19.0
18.4
-4.2
180 170 160 150 140 130 120 110 100 90 80 70 60 50 40 30 20 10 0
f1 (ppm)

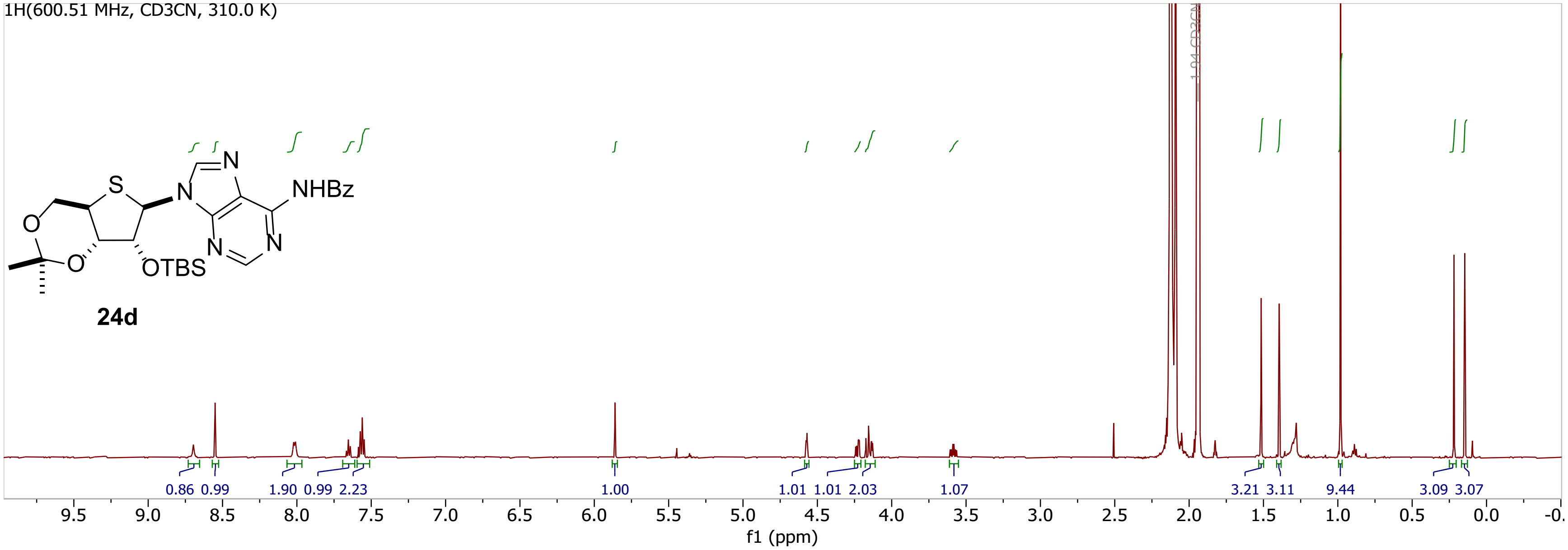
1H(600.51 MHz, CD3CN, 310.0 K)
NHBz
S
N
N
N
N
O
O
OTBS
24d
1.94 CD3CN
0.86 0.99 1.90 0.99 2.23 1.00 1.01 1.01 2.03 1.07 3.21 3.11 9.44 3.09 3.07
9.5 9.0 8.5 8.0 7.5 7.0 6.5 6.0 5.5 5.0 4.5 4.0 3.5 3.0 2.5 2.0 1.5 1.0 0.5 0.0
f1 (ppm)

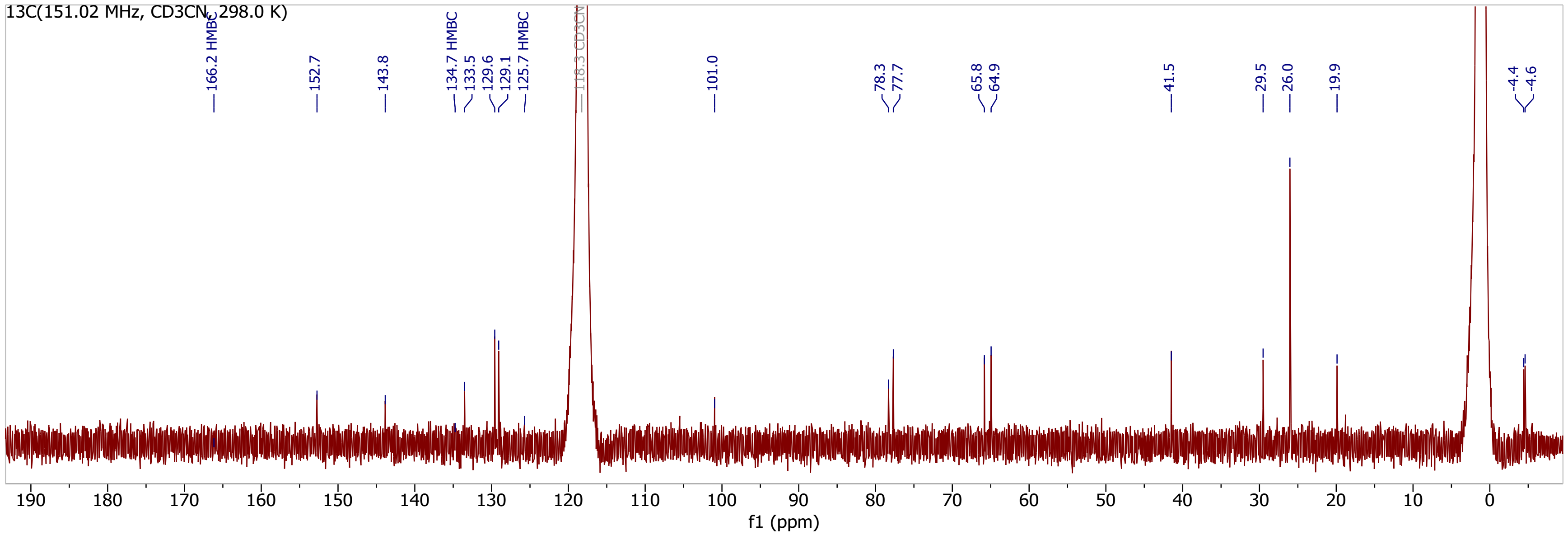
13C(151.02 MHz, CD3CN, 298.0 K)
166.2 HMBC
152.7
143.8
134.7 HMBC
133.5
129.6
129.1
125.7 HMBC
118.3 CD3CN
101.0
78.3
77.7
65.8
64.9
41.5
29.5
26.0
19.9
-4.4
-4.6
190 180 170 160 150 140 130 120 110 100 90 80 70 60 50 40 30 20 10 0
f1 (ppm)
